# Macrophage-secreted interleukin-35 regulates cancer cell plasticity to facilitate metastatic colonization

**DOI:** 10.1038/s41467-018-06268-0

**Published:** 2018-09-14

**Authors:** Chih-Chan Lee, Jiunn-Chang Lin, Wei-Lun Hwang, Ying-Ju Kuo, Hung-Kai Chen, Shyh-Kuan Tai, Chun-Chi Lin, Muh-Hwa Yang

**Affiliations:** 1 Taiwan International Graduate Program in Molecular Medicine, National Yang-Ming University and Academia Sinica, Taipei, Taiwan; 2 Department of Surgery, MacKay Memorial Hospital and MacKay Medical College, Taipei, Taiwan; 3 MacKay Junior College of Medicine, Nursing, and Management, Taipei, Taiwan; 4 Institute of Biotechnology and Laboratory Science in Medicine, National Yang-Ming University, Taipei, Taiwan; 5 Department of Pathology, Taipei Veterans General Hospital, Taipei, Taiwan; 6 Elixiron Immunotherapeutics Inc., Taipei, Taiwan; 7 Department of Otolaryngology, Taipei Veterans General Hospital, Taipei, Taiwan; 8 Division of Colorectal Surgery, Department of Surgery, Taipei Veterans General Hospital, Taipei, Taiwan; 9 Institute of Clinical Medicine, National Yang-Ming University, Taipei, Taiwan; 10 Cancer Progression Research Center, National Yang-Ming University, Taipei, Taiwan; 11 Division of Medical Oncology, Department of Oncology, Taipei Veterans General Hospital, Taipei, Taiwan

## Abstract

A favorable interplay between cancer cells and the tumor microenvironment (TME) facilitates the outgrowth of metastatic tumors. Because of the distinct initiating processes between primary and metastatic tumors, we investigate the differences in tumor-associated macrophages (TAMs) from primary and metastatic cancers. Here we show that dual expression of M1 and M2 markers is noted in TAMs from primary tumors, whereas predominant expression of M2 markers is shown in metastatic TAMs. At metastatic sites, TAMs secrete interleukin-35 (IL-35) to facilitate metastatic colonization through activation of JAK2–STAT6-GATA3 signaling to reverse epithelial–mesenchymal transition (EMT) in cancer cells. In primary tumors, inflammation-induced EMT upregulates IL12Rβ2, a subunit of the IL-35 receptor, in cancer cells to help them respond to IL-35 during metastasis. Neutralization of IL-35 or knockout of IL-35 in macrophages reduces metastatic colonization. These results indicate the distinct TMEs of primary and metastatic tumors and provide potential targets for intercepting metastasis.

## Abstract (web summary)

Tumor microenvironment varies between primary and metastatic sites. Here they report tumor-associated macrophages from metastatic sites to be predominantly M2 type, and secrete IL-35 to promote metastasis through activation of JAK2–STAT6–GATA3 signaling.

## Introduction

The reversible changes between epithelial and mesenchymal phenotypes, i.e., epithelial plasticity, optimally control cellular motility, and regulate cellular differentiation during development as well as cancer metastasis^1^. During the early steps of metastasis, epithelial cancer cells acquire migratory and invasive capabilities through epithelial–mesenchymal transition (EMT). EMT also facilitates the intravasation and extravasation of cancer cells to enable them to reach metastatic sites^2^, and the mesenchymal properties of cancer cells enhances stemness to induce metastatic outgrowth in distant organs^3,4^. In contrast to the involvement of EMT in the early steps of metastasis, several lines of evidence indicate the crucial role of reversal of EMT, i.e., mesenchymal–epithelial transition (MET), in metastatic colonization. First, circulating tumor cells exhibit mesenchymal phenotypes^5^, and platelet-derived TGFβ and direct contacts between platelets and tumor cell facilitate EMT of cancer cells^6^. However, metastatic tumors display an epithelial phenotype similar to that of primary tumors, indicating the occurrence of MET at metastatic sites. Second, MET is crucial during the reprogramming of induced pluripotent stem cells^7,8^. Third, persistent EMT inhibits the formation of metastatic tumors^9^. Furthermore, increasing evidence suggests the occurrence of MET in metastatic tumors^10^, and the interplay between cancer cells and the microenvironment facilitates this process^11,12^. Since the colonization of cancer cells is the most complex and rate-limiting process of metastasis^13^, the mechanism controlling MET occurs in distant organs, and the signals from the tumor microenvironment (TME) that trigger MET are crucial for metastatic establishment. However, in comparison to the extensive studies on the early steps of metastasis, understanding of the mechanism responsible for colonization and supportive niches is limited.

Tumor-associated macrophages (TAMs) are one of the most abundant types of host immune cells in the TME that expedite tumor growth, angiogenesis, immune evasion, and remodeling of the extracellular matrix to facilitate cancer metastasis^14^. Under physiological conditions, macrophages are polarized into M1 (classically activated) macrophages, which are pro-inflammatory with antitumor activity, and M2 (alternatively activated) macrophages, which are antiinflammatory with angiogenic and tissue-remodeling activity^15^. Although TAMs are generally thought to harbor an M2-like phenotype to enhance cancer progression^14^, the phenotype and exact role of TAMs remain debatable. M1-like TAMs with pro-inflammatory and antitumor activities have been observed^16,17^, indicating the phenotypic and functional heterogeneity of TAMs.

Since the outgrowth of primary and metastatic tumors is very different, we hypothesize that the impact of TAMs on primary and metastatic tumors on cancer cells should be different. A limited number of reports have demonstrated the distinct populations of TAMs from primary and metastatic tumors^18,19^. However, the unique role and underlying mechanism of metastatic TAMs remain elusive. In this study, we confirm the distinct features of primary TAMs (pTAMs) and metastatic TAMs (mTAMs). We further show that in metastatic tumors, mTAMs secrete interleukin-35 (IL-35) to activate the JAK2–STAT6–GATA3 signaling axis in cancer cells, which reverses EMT and facilitates the colonization of cancer cells.

## Results

### Characterizing macrophages in primary and metastatic tumors

**Fig. 1:**
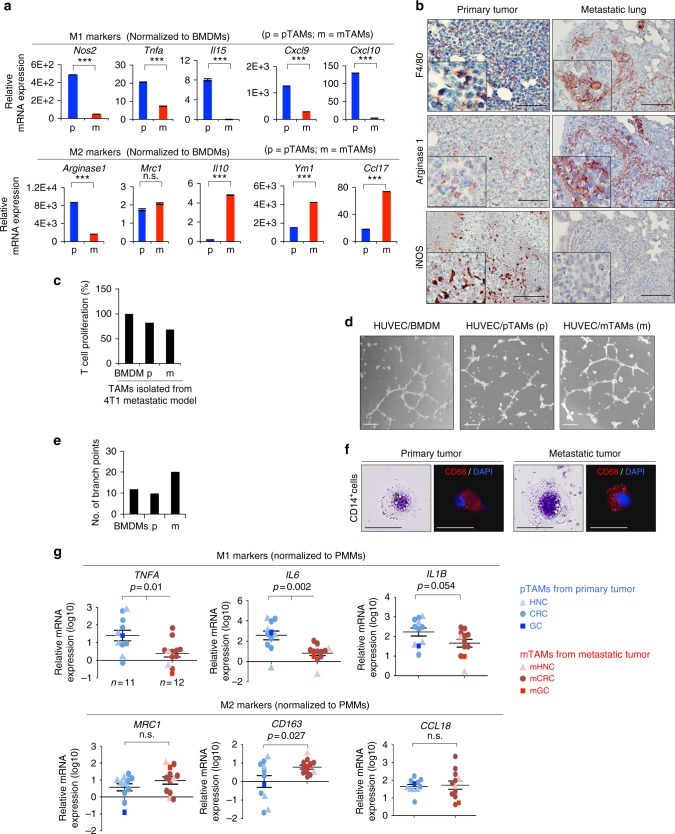
Distinct macrophages population in primary and metastatic tumor. **a** RT-qPCR for analyzing the expression of M1 (*Nos2, Tnfa, Il15, Cxcl9*, and *Cxcl10*) and M2 markers (*Arginase 1*, *Mrc1*, *Il10, Ym1*, and *Ccl17*) of CD11b^+^F4/80^+^ TAMs in the primary tumors (pTAMs; p) and metastatic lungs (mTAMs; m) 5 weeks after inoculation of 4T1 cells. The data were normalized to bone marrow-derived macrophages (BMDM) from healthy mice. *n* = 3 (triplicated RT-qPCR from the same mouse). **b** Representative result of immunohistochemistry of F4/80, Arg-1, and iNOS in matched primary-metastatic tumor pairs for showing the tumor-associated macrophages in 4T1-BALB/c orthotopic syngeneic model. Scale bar, 100 μm. **c** T cell proliferation assay. The CD4^+^ T cells were co-cultured with BMDMs, pTAMs (p), or mTAMs (m) from 4T1 orthotopic model. *n* = 2 independent experiments (each experiment contains three technical replicates). **d** Representative images of HUVEC organization. Scale bar, 50 μm. **e** Quantification of HUVEC tube formation cultivated in different conditioned media as measured by their branching number. *n* = 2 independent experiments (the data of each experiment was the mean value of quantification of four randomly selected fields) **f** Liu’s stain and immunofluorescent stain of CD68 in CD14^+^ cells from primary and metastatic tumor. Scale bar, 20 μm. **g** RT-qPCR for analyzing the expression of M1 (*TNFA*, *IL6*, *IL1B*) and M2 markers (*MRC1*, *CD163*, *CCL18*) of CD14^+^ TAMs in primary (*n* = 11) and metastatic human cancers (*n* = 12). The data were normalized to peripheral blood monocytes-derived macrophages (PMMs) (*n* = 5). Data represent mean ± S.E.M. The *p*-value is show in each panel. n.s. non-significance. HNC head and neck cancer, CRC colon rectal cancer, GC gastric cancer, mHNC metastatic head and neck cancer; mCRC metastatic colon rectal cancer, mGC metastatic gastric cancer. Data represent mean ± S.E.M. *p < 0.05, **p < 0.01, ***p < 0.001. Statistical analysis: Student’s *t*-test (**a**, **g**). See also Supplementary Figure 1

We applied the murine orthotopic breast cancer model by inoculating syngeneic 4T1 mammary cancer cells into BALB/c mice to investigate primary and metastatic TME. Pulmonary metastases developed four to five weeks after inoculation of tumor cells (Supplementary Figure 1a). To understand the composition of immune cells in primary and metastatic tumors, we identified the populations of innate immune cells, including CD11b^+^F4/80^+^ macrophages, CD11b^+^Gr1^+^ myeloid-derived suppressor cells (MDSC), and CD11c^+^ MHCII^+^ dendritic cells (DC); and adaptive immune cells including CD4^+^ T cells, CD8^+^ T cells, and CD4^+^Foxp3^+^ regulatory T cells (Treg), in primary and metastatic tumors. Among innate immune cells, macrophages were the main population, comprising ~20% and 10% CD45^+^ cells in primary and metastatic tumors, respectively. Granulocytic MDSC was also a major population, especially in metastatic tumors. Regarding adaptive immune cells, CD8^+^ T cells were the main population. Metastatic tumors harbored a higher proportion of CD4^+^ T cells as well as Treg (Supplementary Figure 1b, c). Herein, we focused on investigating TAMs due to their abundance in primary as well as metastatic tumors. CD11b^+^F4/80^+^ macrophages were isolated from primary tumors and metastatic lung tissues for further analyses (Supplementary Figure 1d). Next, we examined the expression of relevant markers in pTAMs and mTAMs. The results showed that the pTAMs primarily expressed M1-associated markers. Interestingly, they also expressed certain M2-associated markers (e.g., *Arg1* and *Mrc1*). In contrast, a predominant M2 pattern was noted in the mTAMs (Fig. 1a). Immunohistochemical staining for M1/M2 markers in the harvested primary and metastatic tumors confirmed the findings (Fig. 1b). Functional assays showed that the mTAMs had a higher capability to suppress the proliferation of T cells (Fig. 1c) and promote angiogenesis (Fig. 1d, e). To further address whether the M2-like components of pTAMs/mTAMs harbored different characteristics, we sorted M2 marker-expressing macrophages (F4/80^+^Mrc1^+^) from primary and metastatic tumors (Supplementary Figure 1e). Intriguingly, higher expression of M2 markers was noted in the macrophages from metastatic compared with primary tumors (Supplementary Figure 1f), indicating the different characteristics of TAMs in primary and metastatic tumors. We next examined the expression of immunologic markers in TAMs from primary-metastatic human cancer samples and collected consistent results: pTAMs expressed both M1 and M2 markers, whereas mTAMs predominantly expressed M2 makers (Fig. 1f, g). Collectively, these results suggest that pTAMs and mTAMs express distinct markers and harbor differential functions.

### Metastatic TAMs facilitate the metastatic colonization

**Fig. 2:**
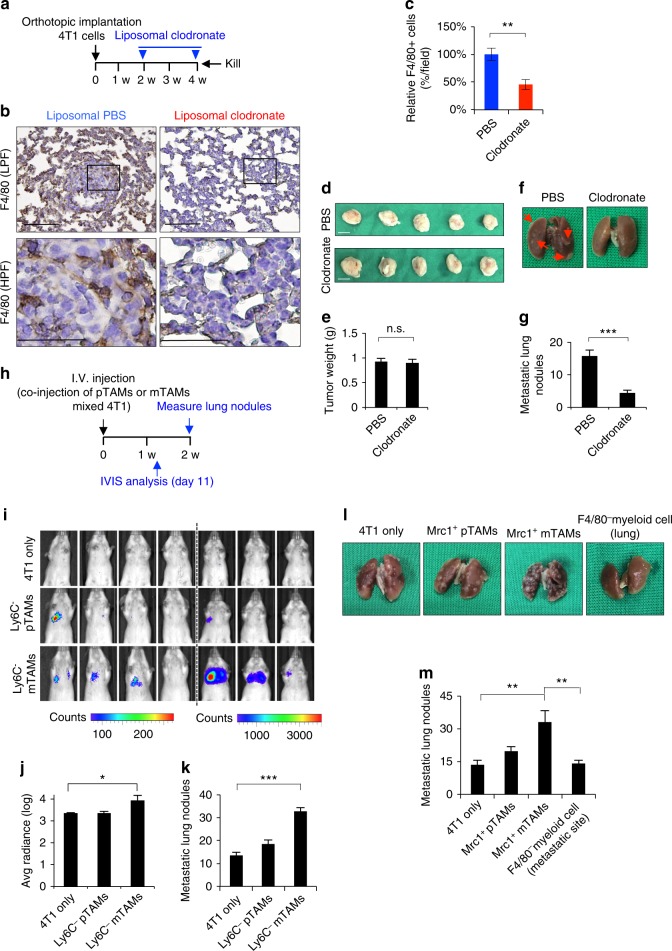
mTAMs facilitates metastatic colonization. **a** Schema of 4T1-BALB/c orthotopic syngeneic model in mice receiving depletion of pulmonary macrophages through intratracheal injection of liposomal clodronate. **b** Representative result for confirming the effect of macrophages depletion. IHC for staining F4/80 was performed in lungs of mice receiving intratracheal liposomal clodronate or vehicle control (PBS). LPF low power field; Scale bar, 100 μm. HPF high power field; Scale bar, 50 μm. **c** Quantification of F4/80^+^ macrophages in lungs of mice receiving intratracheal liposomal clodronate or vehicle control (PBS). The result is present as the fold change of the percentage of F4/80^+^ macrophages in six representative fields. *n* = 5 for each group. **d**, **e** Photos (**d**) and quantification (**e**) of primary tumors. n.s. non-significance. **f**, **g** Photos (**f**) and quantification (**g**) of lung nodules of mice receiving intratracheal liposomal clodronate or control PBS. Red arrows indicate the metastatic tumors in lung. Scale bar, 1 cm. *n* = 5 for each group. **h** Schema of metastatic colonization experiments. 4T1 cells co-injected with CD11b^+^F4/80^+^Ly6C^−^ pTAMs or mTAMs through tail vein of BALB/C mice. **i** IVIS for visualizing tumor dissemination 11 days after tumor cells injection. *n* = 7 for each group. **j** Quantification of bioluminescent imaging of **i** experiment 2 weeks after tumor cells injection. **k** Quantification of metastatic lung nodules of **i** experiment 2 weeks after tumor cells injection. *n* = 6 for each group. **l** Representative photos of lungs of mice 2 weeks after injection of the 4T1 cells with/without Mrc1^+^ TAMs or F4/80^−^ myeloid cell from metastatic tumors. **m** Quantification of metastatic lung nodules. *n* = 6 for each group. Data represent mean ± S.E.M. *p < 0.05, **p < 0.01, ***p < 0.001. Statistical analysis: Student’s *t*-test (**c**, **e**, **g**, **j**, **k**, **m**). See also Supplementary Figure 2

We next investigated the roles of pTAMs and mTAMs in metastasis. We applied liposomal clodronate to deplete macrophages^18,20^ in an orthotopic murine breast cancer model (Supplementary Figure 2a–c). Liposomal clodronate alone did not have a significant impact on the proliferation of 4T1 cells (Supplementary Figure 2d), and injection of clodronate did not affect the number of circulating monocytes (Supplementary Figure 2e–f). Macrophage depletion significantly suppressed both primary tumor growth and pulmonary metastasis (Supplementary Figure 2g–j). Here, we focused on the role of macrophages at metastatic sites. We depleted pulmonary macrophages by intratracheal injection of liposomal clodronate (Supplementary Figure 2k–l), which also did not influence the level of circulating monocytes (Supplementary Figure 2m–n). Next, we observed the impact of pulmonary macrophage depletion on metastatic colonization. Reduction of pulmonary macrophages significantly suppressed pulmonary metastasis without affecting primary tumor weights (Fig. 2a–g). We further investigated the pro-colonization effect of pTAMs and mTAMs. Co-injection of CD11b^+^F4/80^+^Ly6c^−^mTAMs with 4T1 cells via the tail vein increased the colonization of lung tumors compared with CD11b^+^F4/80^+^Ly6c^−^pTAMs (Fig. 2h–k, Supplementary Figure 2o). Consistently, F4/80^+^Mrc1^+^ mTAMs also had a greater ability to promote pulmonary colonization of 4T1 cells than pTAMs or F4/80^−^ myeloid cells (Fig. 2l, m). Taken together, these results suggest that mTAMs harbor a greater ability to facilitate metastatic colonization.

### Macrophages regulates epithelial plasticity of cancer cells

**Fig. 3:**
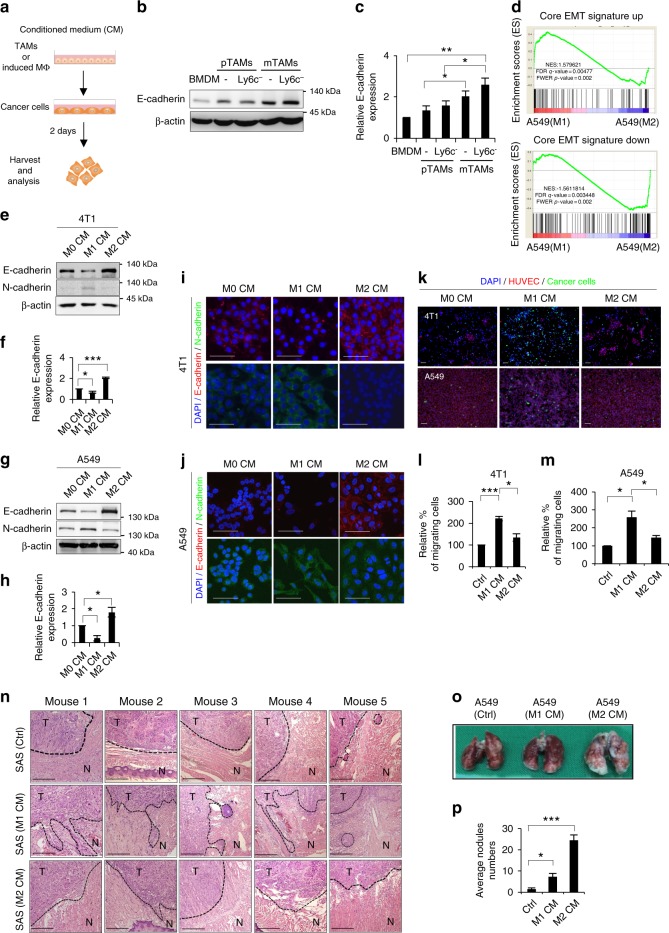
The effect of macrophages on the epithelial plasticity of cancer cells. **a** Schema for the experiments. **b**, **c** The representative result (**b**) and quantification (**c**) of western blots of E-cadherin in 4T1 cells treated with the indicated conditioned media (CM) for 48 h. *n* = 3. BMDM bone marrow-derived macrophages; pTAM, CD11b^+^F4/80^+^ primary tumor-associated macrophages; Ly6C^−^pTAM, Ly6C^−^ CD11b^+^F4/80^+^ primary tumor-associated macrophages; mTAM, CD11b^+^F4/80^+^ metastatic tumor-associated macrophages; Ly6C^−^ mTAMs: Ly6C^−^ CD11b^+^F4/80^+^ metastatic tumor-associated macrophages. **d** GSEA plot for showing the association between M1/M2 CM-regulated signature in A549 cells and core signature in EMT. NES normalized enrichment score, FDR false discovery rate, FWER family-wise error rate. **e**, **f** The representative result (**e**) and quantification (**f**) of western blots for E-cadherin in 4T1 cells upon treatment of the indicated CM for 48 h. *n* = 3. **g**, **h** The representative result (**g**) and quantification (**h**) of western blots of E-cadherin in A549 cells upon treatment of the indicated CM for 48 h. *n* = 3. **i**, **j** Immunofluorescent staining of E-cadherin/N-cadherin in 4T1 and A549 cells upon indicated CM treatment for 48 h. Scale bar, 100 μm. **k** Transendothelial migration assay of 4T1 and A549 cells upon indicated CM treatment. Scale bar, 100 μm. **l**, **m** Quantification of cancer cells of the result (**k**). *n* = 3 independent experiments (the data of each experiment was the mean value of quantification of five randomly selected fields). **n** Hematoxylin & eosin stain of the tumor samples harvested from the orthotopic SAS xenograft. The SAS cells were treated with the indicated CM for 48 h before inoculation. T tumor, N normal tissue. Scale bar, 200 μm. (*n* = 5 for each group). **o** Representative photos of the lungs from mice receiving tail vein injection of A549 cells pretreated with M1 or M2 CM or control media. **p** Quantification of metastatic lung nodules 8 weeks after tumor cells injection (*n* = 6 for each group). Data represent mean ± S.E.M. *p < 0.05, **p < 0.01, ***p < 0.001. Statistical analysis: Student’s *t*-test (**c**, **f**, **h**, **l**, **m**, **p**) and Kolmogorov–Smirnov test for GSEA (**d**). See also Supplementary Figure 3, Supplementary Table 1, Supplementary Data 1

Next, we investigated whether mTAMs could directly influence the colonization of cancer cells by regulating epithelial plasticity since accumulated evidence supports the role of MET in metastatic colonization^11^. We first characterized the EMT phenotype of the cell lines to be used in the study (Supplementary Figure 3a, Supplementary Table 1) and then harvested the conditioned medium (CM) of pTAMs and mTAMs from the 4T1 orthotopic model to treat cancer cells (Fig. 3a). Compared with pTAMs, treatment of 4T1 cells with CM from mTAMs upregulated E-cadherin (Fig. 3b, c). The mTAM CM also reduced the migration of cancer cells (Supplementary Figure 3b, c). Because the mTAMs predominantly showed a M2-like phenotype (Fig. 1), we performed in vitro polarization of human CD14^+^ monocytes into M1-like/M2-like macrophages for subsequent experiments (Supplementary Figure 3d–g). A Gene Set Enrichment Analysis (GSEA) showed that the gene expression signature of the M1 CM-treated A549 cells was significantly correlated with the core EMT signature. In contrast, an inverse correlation between the M2 CM-induced signature and EMT signature was noted (Fig. 3d, Supplementary Data 1). Compared with the CM from the M1 macrophages, the M2 CM induced MET in different cancer cell lines (Fig. 3e–j, Supplementary Figure 3h), suggesting that the M2 secretome influenced the cancer cells to undergo MET to acquire the epithelial phenotype. Regarding the ability to penetrate endothelial cells, M1 CM-treated cancer cells had an enhanced endothelial penetration ability compared with M2 CM-treated cancer cells (Fig. 3k–m, Supplementary Figure 3i–j). Since the epithelial phenotype is disadvantageous for passage through vessel walls, we examined whether the macrophage CM-treated cancer cells could produce factors to facilitate the penetration process. Considering the effect of macrophage CM on the upregulation of factors to increase vascular permeability^21,22^, both M1 and M2 CM-treated cancer cells showed increased expression of angiopoietin-like 4 (ANGPTL-4), and M2-CM-treated cells expressed higher levels of IL-18 (Supplementary Figure 3k–l). Additionally, the supernatant from M1/M2-conditioned cancer cells increased the permeability of HUVECs (Supplementary Figure 3m–n). Furthermore, M2-conditioned cancer cells acquired greater adhesive ability to normal lung epithelial cells (Supplementary Figure 3o–p).

We validated the effect of the M2-like macrophages/mTAMs-regulated epithelial plasticity in vivo and in human samples. In the SAS oral cancer cell orthotopic model, an increase in local invasion was noted in M1 CM-treated SAS cells, and the M2 CM-treated cells formed a localized tumor without peripheral invasion (Fig. 3n). In the A549 pulmonary colonization assay, a significant increase in numbers of metastatic nodules were noted in the group of mice that received the M2 CM-treated cancer cells compared with the M1 CM-treated ones (Fig. 3o, p). In samples from the 4T1-BALB/c syngeneic mice model, increased expression of E-cadherin was noted in metastatic tumors (Supplementary Figure 3q). In matched pairs of primary-metastatic head and neck cancers, pTAMs expressed both M1 and M2 markers, and mTAMs showed a M2-like phenotype as expected. Higher E-cadherin expression was detected in metastatic compared with primary tumors (Supplementary Figure 3r). Taken together, these results suggest that the secretome of M2-like macrophages/mTAMs suppresses EMT, increases vascular permeability, and promotes metastatic colonization.

### JAK–STAT6 pathway of mTAMs regulate metastatic colonization

**Fig. 4:**
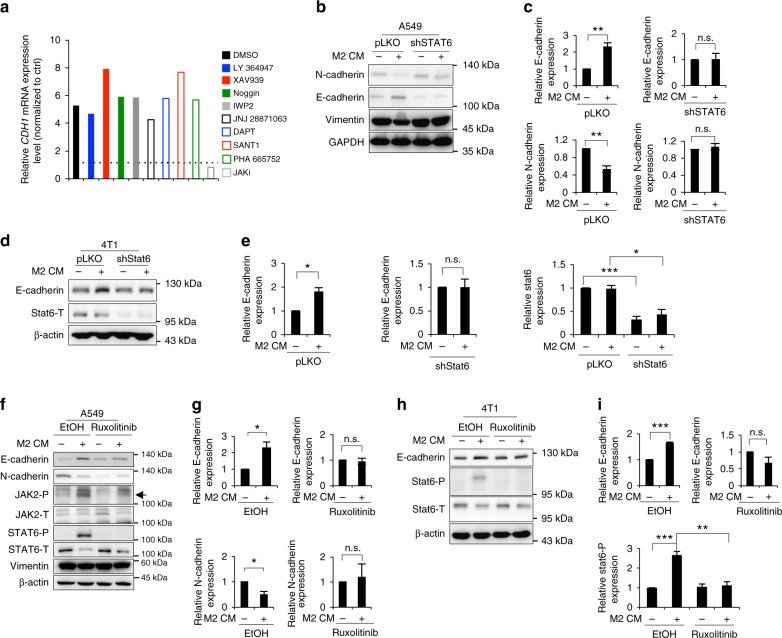
JAK–STAT6 axis reverses EMT. **a** RT-qPCR for *CDH1* in M2-conditioned media (CM)-treated A549 cells under different inhibitors for 48 h. *n* = 2 independent experiments (each experiment contains two technical replicates). **b**, **c** The representative result (**b**) and quantification (**c**) of the EMT markers in A549 infected with a shRNA against STAT6 (shSTAT6) or a control vector (pLKO) upon M2 CM treatment for 48 h. *n* = 3. **d**, **e** The representative result (**d**) and quantification (**e**) of E-cadherin and Stat6 in 4T1 cells infected with shStat6 or a control vector upon M2 CM treatment for 48 h. *n* = 3. **f**, **g** The representative result (**f**) and quantification (**g**) of the EMT markers in M2 CM-treated A549 cells with/without 10 μM ruxolitinib treatment for 48 h. The arrow indicates phosphorylated JAK2. *n* = 3. **h**, **i** The representative result (**h**) and quantification (**i**) of E-cadherin and phosphorylated Stat6 in M2 CM-treated 4T1 cells with/without 10 μM ruxolitinib treatment for 48 h. *n* = 3. Data represent mean ± S.E.M. *p < 0.05, **p < 0.01, ***p < 0.001. Statistical analysis: Student’s *t*-test (**c**, **e**, **g**, **i**). See also Supplementary Figure 4, Supplementary Data 2, Supplementary Data 3

Since M2-like macrophages promote MET and M1-like macrophages potentiate cancer cell EMT and epithelial plasticity is crucial for cancer cell dissemination and colonization^23^, we investigated the mechanism underlying TAM-regulated epithelial plasticity of cancer cells. We analyzed the gene expression profiles of A549 cells treated with M1/M2 CM (Supplementary Data 2 and Supplementary Data 3). By analyzing the M1-induced changes in gene expression, activation of inflammatory signals such as iNOS, IL-6, interferon, and Toll-like receptor signaling was observed in cancer cells, as expected (Supplementary Figure 4a), which is consistent with previous findings showing that inflammation is one of the major triggers of EMT^24–26^. To confirm the role of inflammatory signals in M1-induced EMT, we neutralized IL-6, IL-1β, and TNFα in M1-treated A549 cancer cells and examined the expression of EMT markers. Restoration of E-cadherin and downregulation of N-cadherin were noted after TNFα neutralization (Supplementary Figure 4b–d), suggesting that TNFα plays a predominant role in M1-induced EMT of cancer cells. We next investigated the M2-induced gene expression changes in cancer cells and identified the JAK-STAT signaling pathway as the major activated pathway (Supplementary Figure 4e). To confirm this finding, we screened for the impact of different inhibitors on the expression of E-cadherin, a hallmark of epithelial phenotype, in cancer cells treated with M2 CM. Inhibition of JAK activity downregulated the expression of E-cadherin most significantly (Fig. 4a), supporting the role of the JAK pathway in M2 CM-induced MET.

**Fig. 5:**
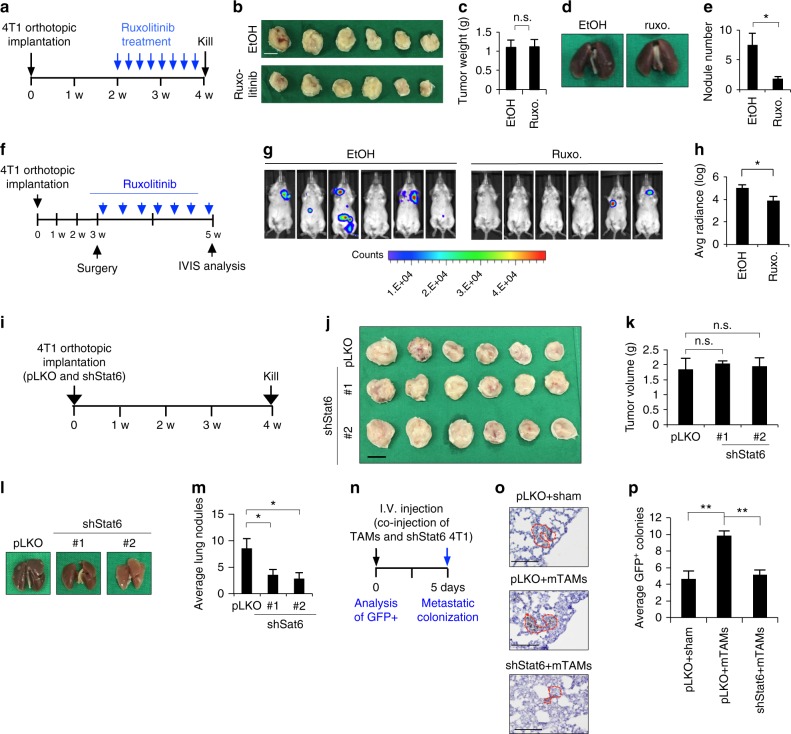
Activation of JAK–STAT6 pathway promotes metastasis. **a** Schema of animal experiment. 4T1-BALB/c syngeneic orthotopic mice receiving 30 mg/kg ruxolitinib or normal saline through intraperitoneal injection every 2 days for 2 weeks started from the end of 2nd week after 4T1 cells inoculation. **b**, **c** Photos (**b**) and quantification (**c**) of primary tumors in (**a**). Scale bar, 1 cm. **d**, **e** Photos (**d**) and quantification (**e**) of lung nodules in (**a**). *n* = 6. **f** Schema of animal experiment. 4T1-BALB/c syngeneic orthotopic mice receiving surgery for removing primary tumor at the end of 3rd week after tumor inoculation. 30 mg/kg ruxolitinib or normal saline was delivered through intraperitoneal injection every 2 days after surgery for 2 weeks, and IVIS images were taken at the end of 5th week. **g** Bioluminescence signals of the mice 2 weeks after surgery. **h** Quantification of bioluminescent imaging. **i** Schema for animal experiment. 4T1 cells with/without Stat6 knockdown were inoculated orthotopically and mice were killed at the end of 4th week. **j**, **k** Photos (**j**) and quantification (**k**) of primary tumors. Scale bar, 1 cm. **l**, **m** Photos (**l**) and quantification (**m**) of lung nodules. *n* = 6. **n** Schema of metastatic colonization experiment. GFP-labeled 4T1 cells with/without Stat6 knockdown were co-injected with Ly6C^−^mTAMs/vehicle control through tail vein, and the mice were killed at 5th days. IHC of GFP was used for analyzing metastatic colonization in lung. **o** Representative images of IHC for GFP of lungs. Scale bar, 100 μm. **p** Quantification of average GPF-positive colonies from 5 paraffin-embedded lung section. *n* = 6. Data represent mean ± S.E.M. *p < 0.05, **p < 0.01, ***p < 0.001. Statistical analysis: Student’s *t*-test (**c**, **e**, **h**, **k**, **m**, **p**). See also Supplementary Figure 4

We next sought to identify the downstream signal that mediates JAK-induced MET. To achieve this goal, we screened the effect of the signal transducer and activator of transcription (STAT) family by individually knocking down each of the STAT members in cancer cells treated with M2 CM and examined the impact on epithelial and mesenchymal markers (Supplementary Figure 4f). Knockdown of STAT6, but not the other STAT members, inhibited M2 CM-induced MET (Fig. 4b–e, Supplementary Figure 4g). Inhibition of JAK activity with the pan-JAK inhibitor ruxolitinib suppressed M2 CM-induced phosphorylation of STAT6 and MET (Fig. 4f–i). Treatment of 4T1 orthotopic model mice with ruxolitinib significantly suppressed metastasis without significant effect on the growth of the primary tumor (Fig. 5a–e). Furthermore, 4T1 tumors were implanted orthotopically and removed 3 weeks after inoculation (Supplementary Figure 4h–i). Ruxolitinib treatment was initiated after tumor removal and continued for 2 weeks. A marked reduction of metastasis was observed in the mice treated with ruxolitinib (Fig. 5f–h).

To further investigate the impact of JAK–STAT6 suppression on metastasis, we orthotopically implanted Stat6-knockdown 4T1 cells in mice and found that the knockdown of Stat6 reduced metastasis without affecting the growth of the primary tumor (Fig. 5i–m, Supplementary Figure 4j-k). Additionally, we confirmed the role of STAT6 in metastatic colonization. Stat6-knockdown 4T1 cells or control cells were co-injected with CD11b^+^F4/80^+^Ly6C^−^ mTAMs via the mouse tail vein. Knockdown of Stat6 reduced mTAM-induced metastatic colonization (Fig. 5n–p). Nevertheless, the sizes of the colonized tumors were not significantly different between the 4T1-control and 4T1-mTAM groups (Supplementary Figure 4l–m), indicating that mTAMs mostly affected metastatic colonization rather than outgrowth. Taken together, these results suggest that JAK–STAT6 is the major pathway responsible for M2-mediated MET and metastatic colonization.

### STAT6 activates the *GATA3* transcription in metastatic tumors

**Fig. 6:**
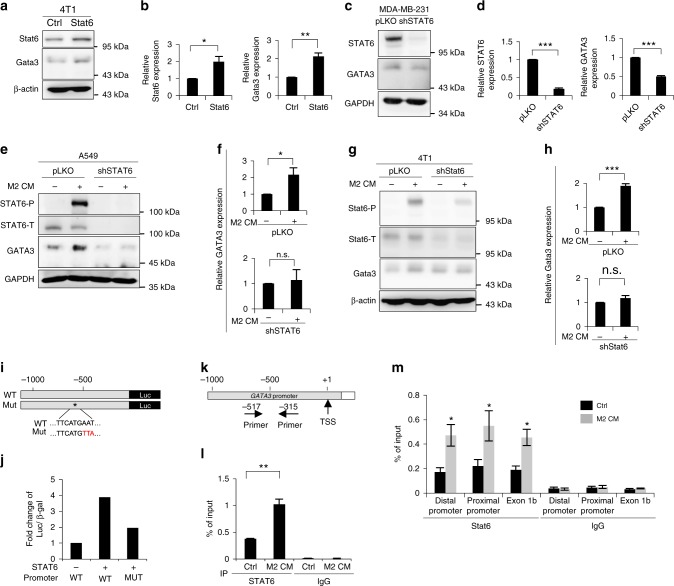
Direct regulation of *GATA3* by STAT6. **a**, **b** The representative result (**a**) and quantification (**b**) of STAT6 and GATA3 in 4T1 cells infected with a STAT6 expression vector or a control vector. *n* = 3. **c**, **d** The representative result (**c**) and quantification (**d**) of STAT6 and GATA3 in MDA-MB-231 cells infected shSTAT6 or a control sequence (pLKO). *n* = 3. **e** The representative result (**e**) and quantification (**f**) of GATA3 in A549 cells infected with shSTAT6 or control with/without M2 CM treatment for 48 h. *n* = 3. **g**, **h** The representative result (**g**) and quantification (**h**) of Gata3 in 4T1 cells infected with shStat6 or control with/without M2 CM for 48 h. *n* = 3. **i** Representation of the reporter constructs. **j** Luciferase reporter assay in HEK-293T transfected with indicated plasmids. *n* = 2 independent experiments (each experiment contains two technical replicates). **k** Chromatin immunoprecipitation (ChIP). Organization of the promoter region of *GATA3*. TSS transcription start site. The primers for amplification of the DNA-binding region are indicated. **l** ChIP assay. A549 cells were treated with M2 CM/control media for 24 h. The enrichment values were normalized to the input of immunoprecipitation. *n* = 3 independent experiments (each experiment contains two technical replicates). **m** ChIP assay. 4T1 were treated with M2 CM/control media for 24 h. *n* = 3 independent experiments (each experiment contains two technical replicates). Data represent mean ± S.E.M. *p < 0.05, **p < 0.01, ***p < 0.001. Statistical analysis: Student’s *t*-test (**b**, **d**, **f**, **h**, **l**, **m**). See also Supplementary Figure 5

**Fig. 7:**
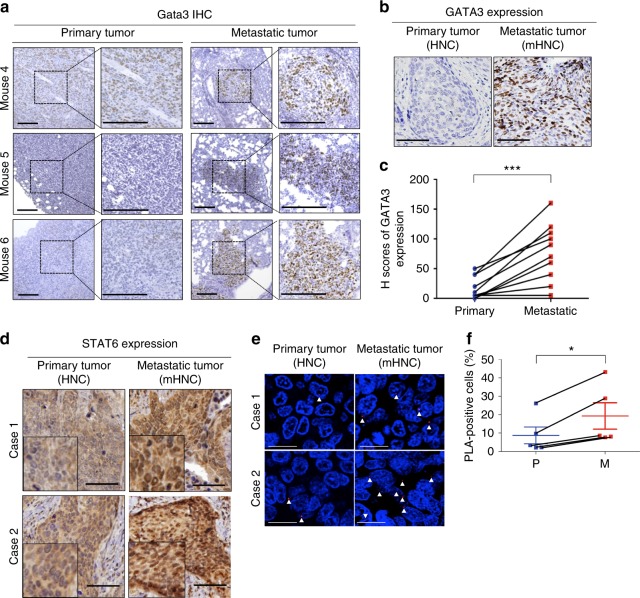
Activation of STAT6–GATA3 axis in metastatic tumors. **a** Immunohistochemistry (IHC) of Gata3 in primary-metastatic paired sample from three mice. Scale bar, 200 μm. **b** Representative images of IHC of GATA3 in paired primary-metastatic tumors from 10 patients. Scale bar, 100 μm. HNC head and neck cancers, mHNC metastatic head and neck cancer. **c** Quantification of IHC by H score. **d** IHC of STAT6 in paired primary-metastatic sample. Scale bar, 100 μm. **e** Proximity ligation assay (PLA) for detecting phosphorylated STAT6 by dual staining of the anti-STAT6 and the anti-pan-phosphorylated tyrosine antibodies in paired head and neck cancer patient sample (*n* = 5). Scale bars, 20 μm. The arrows indicate the representative PLA-positive signals. **f** Quantification of PLA-positive cells. P Primary tumor, M Metastatic tumor. Data represent mean ± S.E.M. *p < 0.05, **p < 0.01, ***p < 0.001. Statistical analysis: Student’s *t*-test (**c**, **f**). See also Supplementary Figure 5

To explore the potential downstream targets of JAK/STAT6-mediated MET, we examined the expression of GATA3 in cancer cells because GATA3 is a transcription factor that inhibits EMT and is expressed in early-stage well-differentiated cancers but decreases in advanced invasive cancers^27–29^, and STAT6 has been shown to activate GATA3 during T cell differentiation^30,31^. GATA3 expression was elevated in M2 CM-treated compared with M1 CM-treated cancer cells (Supplementary Figure 5a, b). To investigate whether STAT6 directly activated GATA3, we overexpressed STAT6 in various cancer cells and observed the upregulation of GATA3 expression (Fig. 6a, b, Supplementary Figure 5c, d). Knockdown of STAT6 suppressed the expression of both endogenous GATA3 (Fig. 6c, d, Supplementary Figure 5e, f) and M2 CM-induced GATA3 (Fig. 6e–h). A reporter assay showed that transfection of wild-type STAT6 increased the promoter activity of *GATA3*. Mutation of the STAT6-binding site attenuated the STAT6-induced activation of the *GATA3* promoter (Fig. 6i, j). The chromatin immunoprecipitation assay confirmed the direct binding of STAT6 to the *GATA3* promoter after treatment with M2 CM (Fig. 6k–m). Given that a reversible EMT program is necessary for the establishment of metastatic tumors with epithelial phenotypes, we further identified the expression of GATA3 in three metastatic mouse models. In the 4T1 orthotopic model, the expression of Gata3 in the metastatic tumors was significantly higher than that in the primary tumor (Fig. 7a, Supplementary Figure 5g–i). Increased levels of Gata3/GATA3 were also noted in another syngeneic model of LLC tumors (Supplementary Figure 5j–k) and the orthotopic xenotransplantation model with SAS cells (Supplementary Figure 5l, m). We further examined the expression of GATA3 in 10 matched primary-metastatic tumor samples from head and neck cancer patients, and the level of GATA3 was higher in the metastatic tumors (Fig. 7b, c and Supplementary Figure 5n). STAT6 was predominantly expressed in the nuclei of the metastatic tumor cells but rarely in the primary tumors (Fig. 7d). In addition, a higher percentage of phosphorylated STAT6 (detected by proximity ligation assay) was found in the metastatic head and neck cancer samples (Fig. 7e, f), suggesting the activation of STAT6 in the metastatic tumors. In summary, the above results indicate that STAT6-induced GATA3 activation is critical for metastatic colonization.

### mTAMs secrete IL-35 to facilitate cancer colonization

**Fig. 8:**
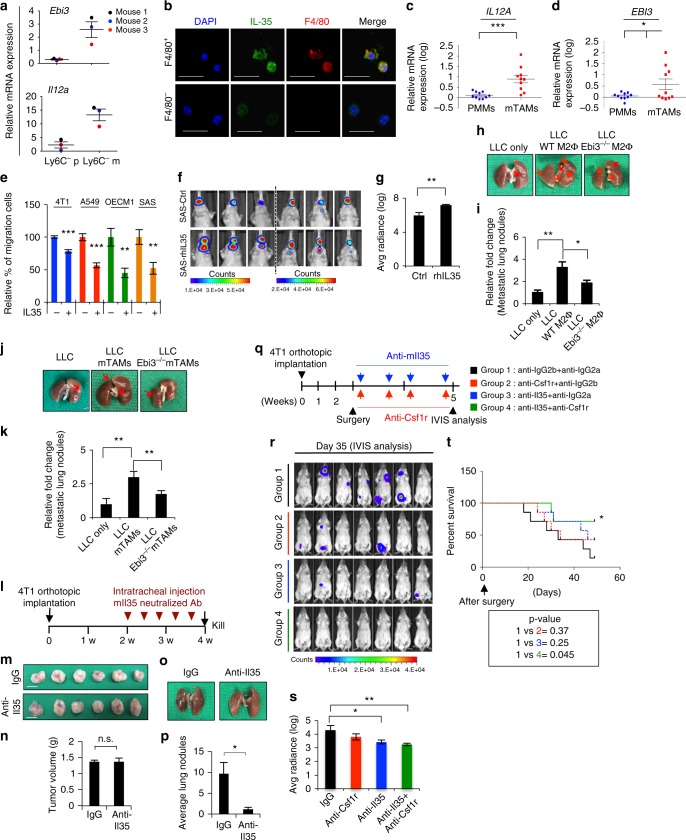
mTAMs-secreted IL-35 facilitates metastatic colonization. **a** RT-qPCR for *Il12a* and *Ebi3* in Ly6C^−^ TAMs from the matched primary tumors and lungs of 4T1-BALB/c syngeneic orthotopic mice 5 weeks after 4T1 cells inoculation (*n* = 3). The data were normalized to BMDM from healthy mice (*n* = 3). **b** Immunofluorescent staining of Il-35 (green) and F4/80 (red) in Ly6C^−^F4/80^+^ and Ly6C^−^F4/80^−^ cells from lungs of 4T1-BALB/c syngeneic orthotopic mice. Blue, nuclei. Scale bar, 50 μm. **c**, **d** RT-qPCR for analyzing the expression of *IL12A* and *EBI3* in CD14^+^ TAMs from metastatic human tumors (*n* = 10) versus peripheral blood monocyte-derived macrophages (PMMs) (*n* = 10). **e** Transwell migration assay. Recombinant human/murine IL-35 (50 ng ml^−1^) treatment duration: 48 h. *n* = 3 independent experiments (the data of each experiment was the mean value of quantification of at least five randomly selected fields). **f** Orthotopic xenograft experiment. SAS cells were pretreated with rhIL-35 (50 ng ml^−1^) or control for 48 h before inoculation. IVIS images were taken 14 days after tumor inoculation (*n* = 6 for each group). **g** Quantification of bioluminescent imaging. **h** Representative photos of lungs 2 weeks after injection of the LLC cells with/without co-injection of the WT or *Ebi3*^−*/*−^ M2-like macrophages. **i** Quantification of metastatic lung nodules. *n* = 5 for each group. **j** Representative photos of lungs 2 weeks after injection of the LLC cells with/without co-injection of mTAMs from WT or *Ebi3*^−*/*−^ mice. **k** Quantification of metastatic lung nodules. *n* = 4 for each group**. l** Schema for animal experiment. **m**, **n** Photos (**m**) and quantification (**n**) of primary tumor. Scale bar, 1 cm. **o**, **p** Photos (**o**) and quantification (**p**) of lung nodules. *n* = 6. **q** Schema for presenting the antibody therapy experiment. *n* = 7 for each group. **r**, **s** Bioluminescence signal (**r**) and quantification (**s**). **t** Kaplan–Meier analysis of the survival of mice after antibody administration. Data represent mean ± S.E.M. *p < 0.05, **p < 0.01, ***p < 0.001. Statistical analysis: Student’s *t*-test (**c**, **d**, **e**, **g**, **i**, **k**, **n**, **p**, **s**) and log-rank test for survival curve (**t**). See also Supplementary Figure 6, Supplementary Data 4

To elucidate the factor(s) involved in mTAM-mediated cancer colonization, a cDNA microarray was performed in pTAMs/mTAMs from the 4T1-BALB/c syngeneic model. We narrowed down the candidate factors from mTAMs to those located upstream of the JAK/STAT signaling pathway. IL-35 (composed of IL-12α and IL-27β) and IL-4 were the most likely factors mediating mTAM-induced metastatic colonization (Supplementary Figure 6a, b, Supplementary Data 4). A high level of Il-4 was detected in mTAM CM (Supplementary Figure 6c), which is in agreement with a previous work^32^. Although increased expression of *Epstein-Barr virus-induced gene 3* (*EBI3*), which encodes the IL-35 subunit IL-27β, has been noted in M2 macrophages^33^, the role of IL-35 is relatively unclear in comparison to knowledge of IL-4 in cancer metastasis^34^. Since mTAMs display the M2-like phenotype and understanding of IL-35 in cancer metastasis is limited, we focused on investigating the role of IL-35 in subsequent experiments. In the 4T1-BALB/c syngeneic tumor model, an upregulation of *Ebi3* and *Il12a* was confirmed in mTAMs compared with pTAMs (Fig. 8a). In the lungs of these mice, expression of Il35 was noted in F4/80^+^ TAMs but not F4/80^−^ cells (Fig. 8b). The mTAMs expressed and secreted higher levels of Il-35 compared with pTAMs/BMDMs (Supplementary Figure 6d–e). The in vitro-polarized human M2 macrophages also secreted high levels of IL-35 (Supplementary Figure 6f). In the metastatic human cancer samples, mTAMs expressed higher levels of *EBI3* and *IL12A* compared with peripheral blood monocyte-derived macrophages (PMMs) (Fig. 8c, d). Treatment with IL-35 activated JAK–STAT6 signaling, induced MET, and decreased migration (Fig. 8e, Supplementary Figure 6g–j). Inoculation of IL35-pretreated SAS cells into the tongues of nude mice increased metastatic colonization (Fig. 8f, g). To confirm the effect of macrophage-secreted Il35 in cancer metastasis, we used *Ebi3*^−/−^ mice to conduct the following experiments. First, we purified bone marrow-derived macrophages (BMDM) from WT and *Ebi3*^−/−^ mice and polarized them as M2-like macrophages. The depletion of Il35 in macrophages from *Ebi3*^−/−^ mice was confirmed (Supplementary Figure 6k). Next, we injected the macrophages together with LLC cells in C57BL/6 mice via the tail vein. Co-injection of *Ebi3*^−/−^ macrophages significantly reduced the pulmonary colonization of LLC cells compared with the effect of WT mouse-derived macrophages (Fig. 8h, i). We further generated mTAMs from WT and *Ebi3*^−/−^ mice by inoculating LLC cells into the subcutaneous region of WT and *Ebi3*^−/−^ mice, and mTAMs were obtained from metastatic lung tumors. The depletion of Il35 in mTAMs was validated (Supplementary Figure 6l). We co-injected WT- or *Ebi3*^−/−^-mTAMs with LLC cells into C57BL/6 mice via the tail vein. Consistently, significantly reduced colonization, but not complete abrogation, of lung tumors was observed in the mice that received *Ebi3*^−/−^ mTAMs compared with WT mTAMs (Fig. 8j, k). The expression levels of both nuclear Stat6 and Gata3 were noted in metastatic lung tumors, confirming the essential role of Stat6–Gata3 signaling in the establishment of metastasis. However, the level of Stat6/Gata3 did not differ significantly among the tumors generated from the group co-injected with tumor only, WT macrophages, or *Ebi3*^−*/*−^ macrophages (Supplementary Figure 6m, n), raising the possibility of another source of Il35 in addition to TAMs.

We next tested the therapeutic efficacy of the neutralization of Il-35. The anti-Il35 antibody did not affect the proliferation of 4T1 cells (Supplementary Figure 6o). Intratracheal injection of anti-Il35 antibodies in 4T1-tumor-bearing mice significantly reduced lung metastasis without affecting primary tumor growth (Fig. 8l–p). To validate the role of IL-35 in metastatic colonization, we removed the 4T1 xenograft tumors from the BALB/c mice 3 weeks after implantation, and the mice were administered antibodies against Il-35, colony-stimulating factor 1 receptor (Csf1r), or both, or control IgG (Fig. 8q, Supplementary Figure 6p–t). Administration of either anti-Il35 or anti-Csf1r antibody reduced the development of metastasis, and the combination of anti-Il35 and anti-Csf1r antibodies yielded the best effect on preventing metastasis (Fig. 8r, s). The mice that received combination treatment also displayed better survival than those in the other groups (Fig. 8t). Collectively, these results indicate that macrophage-secreted IL-35 promotes metastatic colonization through the JAK–STAT6 pathway.

### Expression of IL12Rβ2 promotes cancer colonization

**Fig. 9:**
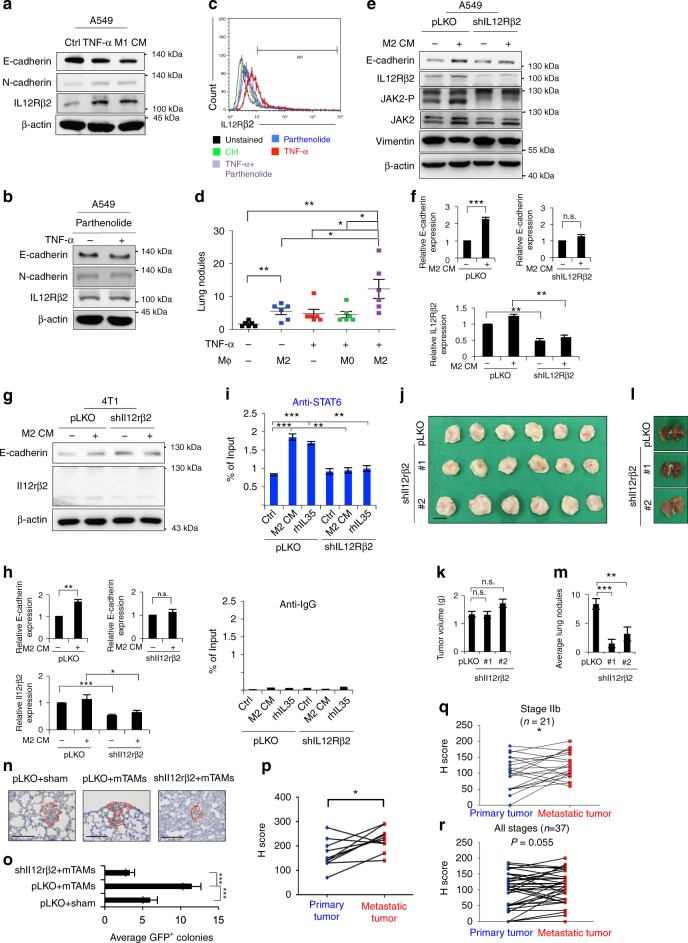
TNFα-primed cancer cells express IL12Rβ2 for metastasis. **a** IL12Rβ2 and EMT markers in A549 treated with TNFα (20 ng ml^−1^), M1 CM, or control for 24 h. **b** IL12Rβ2 and EMT markers in A549 upon TNFα treatment with/without parnetholide for 24 h. **c** Flow cytometry for detecting IL12Rβ2 in A549 upon TNFα (20 ng ml^−1^) treatment with/without parnetholide for 24 h. **d** Quantification of the metastatic lung nodules in mice receiving 1 × 10^6^ A549 with/without TNFα (20 ng ml^−1^) pretreatment for 48 h and 5 × 10^5^ resting (M0)/M2 macrophages. Mice were killed 2 months after injection. *n* = 6. **e**, **f** The representative result (**e**) and quantification (**f**) of IL12Rβ2 and E-cadherin in A549 infected with shIL12RB2 or control (pLKO) with/without M2 CM for 48 h. *n* = 3. **g**, **h** The representative result (**g**) and quantification (**h**) of Il12rβ2 and E-cadherin in 4T1 infected shIl12rb2 or control with/without M2 CM for 48 h. *n* = 3. **i** ChIP in A549 infected with shIL12RB2/control and treated with M2 CM, rhIL-35, or a control media for 24 h. *n* = 3 independent experiments (each experiment contains two technical replicates). **j**, **k** The 4T1-BALB/c orthotopic tumor experiment. The tumors were harvested 4 weeks after tumor implantation. Photos (**j**) and quantification (**k**) of primary tumor. Scale bar, 1 cm. **l**, **m** Photos (**l**) and quantification (**m**) of lung nodules. *n* = 6. **n** Metastatic colonization assay. The GFP-labeled 4T1 with/without Il12rb2 knockdown were co-injected with Ly6C^−^F4/80^+^mTAMs from 4T1-BALB/c syngeneic tumor model, and the lungs were harvested 5 days after injection. *n* = 6. IHC for staining GFP in representative sections of lungs. Scale bar, 100 μm. **o** Quantification of IHC results by counting the average GFP^+^ colonies from five tissue sections. **p** IHC of IL12Rβ2 in paired primary-metastatic head and neck cancers. Quantification of IHC by H score. *n* = 10. **q**, **r** IHC of IL12Rβ2 in 37 paired primary-metastatic breast cancer samples. Quantification of IHC by H score. **q** Stage IIb cases; **r** all-stage cases. Data represent mean ± S.E.M. *p < 0.05, **p < 0.01, ***p < 0.001. Statistical analysis: Student’s *t*-test (**d**, **f**, **h**, **i**, **k**, **m**, **o**, **p**, **q**, **r**). See also Supplementary Figure 7, Supplementary Table 2 and 3

We next investigated the expression of the IL-35 receptor on cancer cells in terms of recognizing signals from TAMs. Since TNFα is the major cytokine responsible for M1-type macrophage-induced EMT (Supplementary Figure 4), we examined whether TNFα-primed cancer cells harbored IL-35 receptor to receive signals at metastatic sites. The IL-35 receptor is a heterodimer comprising IL12Rβ2 and gp130^35^. Treatment of A549 cells with M1 CM or TNFα induced EMT as expected. Interestingly, both M1 CM and TNFα upregulated IL12Rβ2 (Fig. 9a). TNFα upregulated *IL12RB2* expression in different cancer cell lines (Supplementary Figure 7a). Inhibition of NF-κB activity by parthenolide attenuated the expression of TNFα-induced IL12Rβ2 (Fig. 9b, c, Supplementary Figure 7b, c). Next, we elucidated the role of TNFα-primed cancer cells in metastatic colonization through the IL35-mediated signal. Co-injection of M2 macrophages with A549 increased the pulmonary colonization of cancer cells, and the effect was more significant in TNFα-primed cancer cells (Fig. 9d). Knockdown of *IL12RB2* in cancer cells abrogated M2 CM-induced JAK activation and MET (Fig. 9e–h, Supplementary Figure 7d–e). Treatment with M2 CM or IL-35 enhanced the binding of STAT6 to the promoter of *GATA3*, and knockdown of *IL12RB2* abrogated this effect (Fig. 9i). In the 4T1-BALB/c syngeneic tumor model, suppression of *IL12RB2* reduced metastasis without affecting the growth of the primary tumor (Fig. 9j–m, Supplementary Figure 7e). Knockdown of Il12rb2 also abrogated mTAM-induced metastatic colonization (Fig. 9n, o).

Finally, we confirmed the finding in human cancer samples. Analyses of public databases revealed that high levels of *IL12RB2* in cancer samples were associated with worse survival of lung cancer patients (Supplementary Figure 7f). IHC examination for head and neck cancers showed that high levels of IL12Rβ2 correlated with a higher probability of subsequent development of metastasis (Supplementary Figure 7g–i, Supplementary Tables 2 and 3). Moreover, we performed IHC to analyze the expression of IL12Rβ2 in paired primary-metastatic tumor samples, including 10 paired head and neck cancers and 37 paired breast cancers. The expression of IL12Rβ2 was higher in the metastatic tumors in head and neck cancers as well as breast cancers, and the effect was more prominent in early breast cancers (Fig. 9p–r, Supplementary Figure 7j–k). Collectively, these data indicate that inflammation-induced EMT upregulates the expression of IL12Rβ2 in cancer cells, which is critical for cancer cells to respond to IL-35 from mTAMs to complete metastatic colonization.

**Fig. 10:**
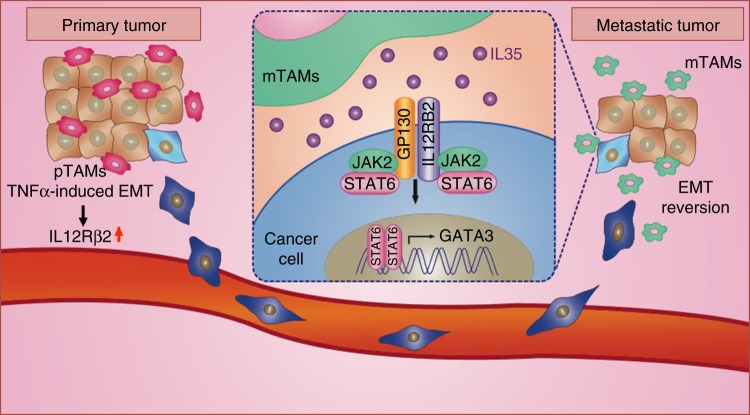
A proposed model of macrophages-regulated epithelial plasticity during metastasis. In primary tumor, TNFα induces EMT and expression of IL12Rβ2 in cancer cells, which promotes tumor invasion and migration. In metastatic sites, mTAMs secrete IL-35 to activate JAK2–STAT6 axis in cancer cells, which promotes MET through activating GATA3, MET of cancer cells at metastatic sites facilitates colonization

We summarize our finding in a schematic in Fig. 10. In the primary tumor, TNFα induces EMT and expression of IL12Rβ2 to promote tumor invasion and migration. At metastatic sites, mTAMs secrete IL-35, which activates the JAK–STAT6 axis to induce GATA3 activation, which leads to MET and metastatic colonization.

## Discussion

In this study, we demonstrated that IL-35 is crucial for mediating the colonization of mesenchymal-like cancer cells in metastatic organs. IL-35 signaling is transduced through the STAT1-STAT4 heterodimer to activate the expression of the IL-35 subunits *IL12A* and *EBI3* in the form of a positive feedback loop^35,36^. The most well-recognized function of IL-35 is its secretion by Tregs and activity as an immunosuppressive cytokine to suppress the proliferation and functions of effector T cells^37,38^. Recently, increasing evidence indicates the involvement of IL-35 in human cancers. In primary tumors, IL-35 suppresses antitumor immunity^39^, and the expression of IL-35 in cancer cells promotes tumor progression and metastasis^40,41^. However, the exact role of IL-35 in the metastatic process is elusive. Here, we showed that at the metastatic sites, macrophages secrete IL-35 to revert the EMT of cancer cells to facilitate metastatic colonization, the rate-limiting process of metastasis. These results illuminate the direct effect of macrophage-secreted IL-35 on cancer colonization, which greatly extend our understanding of IL-35 beyond immunosuppression.

The epithelial plasticity of cancer cells through the dynamic control of EMT and MET is considered an essential property for completing metastasis^23^. Here, we suggest that two cytokines are responsible for the regulation of epithelial plasticity of cancer cells in primary and metastatic tumors: TNFα is responsible for inducing EMT in primary tumors, whereas IL-35 promotes MET in metastatic tumors. It is known that TNFα-mediated inflammatory EMT is important for the initiation of metastasis^42,43^, and our results support this notion. For IL-35 secretion in metastatic tumors, Tregs should be considered in addition to mTAMs since knockout of Il35 in mTAMs herein significantly reduced but did not completely abrogate metastatic colonization, and IL-35-mediated JAK–STAT6–GATA3 signals formed in metastatic tumors even with *Ebi3*^−*/*−^ mTAMs. Although we still consider mTAMs as the major source of IL-35 in metastatic tumors according to their abundance and significant effect when depleting Il35 in mTAMs, the role of Treg-secreted IL-35 should also be considered. Blockade of Il-35 is thereby more effective than depletion of macrophages by anti-Csf1r as shown in our study.

Regarding IL-35-activated signals in metastatic tumor cells, GATA3 activated by STAT6 is the main pathway induced by IL-35. GATA3 has been shown to maintain epithelial cell differentiation through suppression of the EMT factor Zeb2^44^. Low levels of GATA3 in primary breast cancers correlate with a worse prognosis^29,44^. Here, we found that metastatic tumors express higher levels of GATA3 compared with primary tumors. However, the prognostic impact of GATA3 may exist only in primary tumors and not in metastatic lesions. A large-scale screen for the expression of GATA3 in metastatic lesions from different types of primary tumors will be helpful for elucidating the role of GATA3 in metastasis.

In summary, our study demonstrates a mechanism of inflammation-induced epithelial plasticity for regulating metastasis and highlights the role of macrophages in the colonization of metastatic cancer cells through IL-35-mediated signaling. These results provide a potential resolution for controversies regarding the necessity of EMT in metastasis since reversible changes between epithelial and mesenchymal states rather than EMT alone are required to complete the metastatic process. Furthermore, these findings offer several valuable targets for intercepting the metastatic process to prevent the dissemination or recurrence of cancer.

## Methods

### Cell lines, plasmids, and reagents

The human head and neck cancer cell line SAS, human embryonic kidney cell line 293T, human lung cancer cell line A549, human breast cancer cell line MDA-MB-231 and MCF-7, human bronchial epithelial cell line NL20, BALB/c mouse breast carcinoma cell line 4T1, and C57BL/6 mouse lung carcinoma cell line LLC1 were purchased from the Bioresource Collection and Research Center of Taiwan. The human head and neck cancer cell line OECM1 was provided by Dr. Kuo-Wei Chang (National Yang-Ming University of Taiwan). All the human cell lines used in our studies have been tested for Mycoplasma contamination and authenticated by STR method. The pLKO.1-control (ASN0000000004; TRCN0000231722), STAT1#1 (TRCN0000280021), STAT1#2 (TRCN0000004265), STAT2#1 (TRCN0000007460), STAT2#2 (TRCN0000364399), STAT3#1 (TRCN0000020842), STAT3#2 (TRCN0000020843), STAT4#1 (TRCN0000020895), STAT4#2 (TRCN0000020898), STAT6#1 shRNA (TRCN0000019413), STAT6#2 shRNA (TRCN0000274156), mStat6#1 shRNA (TRCN0000218520), mStat6#2 shRNA (TRCN0000226180), IL12RB2#1 shRNA (TRCN0000436750), IL12RB2#2 shRNA (TRCN0000058158), mIl12rb2#1 shRNA (TRCN0000067721), and mIl12rb2#2 shRNA (TRCN0000067722) were purchased from the National RNAi Core Facility of Taiwan for gene silencing. The pCMV-STAT6-IRES-Neo was purchased from Addgene (Cambridge, MA). The pUNO1-hIL35 plasmid was purchased from InvivoGen (San Diego, CA). Recombinant human interferon-γ, interleukin-4, macrophage colony-stimulating factor (M-CSF), granulocyte-macrophage colony-stimulating factor (GM-CSF), Noggin, and recombinant murine interleukin-4 were purchased from PeproTech (Rocky Hill, NJ). Recombinant human TNFα was purchased from Abbiotec (cat. no. 600173, Abbiotec, Inc., San Diego, CA). Recombinant human IL-35 was purchased from BioLegend (cat. no. 578502, BioLegend, Inc., San Diego, CA). Recombinant murine Il-35 (cat. no. SRP8053) was purchased from Sigma- Aldrich (St. Louis, MO). The NF-κB inhibitor parthenolide was purchased from BioVision (cat. no. 1868-10, BioVision Inc., Milpitas, CA). The MET inhibitor PHA 665752 (cat. no. 2693), EGFR inhibitor JNJ 28871063 (cat. no. 3352), Wnt signaling inhibitor IWP2 (cat. no. 3533), and TGF-β receptor inhibitor LY364947 (cat. no. 2718) were purchased from R&D Systems, Inc. (Minneapolis, MN). Lipopolysaccharide, dexamethasone, Wnt signaling inhibitor XAV939 (cat. no. X3004), Notch signaling inhibitor DAPT (cat. no. D5942), and sonic hedgehog signaling inhibitor SANT1 (cat. no. S4572) were purchased from Sigma-Aldrich (St. Louis, MO). JAK inhibitor I (cat. no. 420099) was purchased from EMD Millipore (Billerica, MA). The JAK1/2 inhibitor, ruxolitinib, was provided by Novartis International AG (Basel, Swizerland). The working concentrations for inhibitors used in this study are detailed in Supplementary Table 4.

### Animal experiments

The animal experiment was approved by the Institutional Animal Care and Utilization Committee of Taipei Veterans General Hospital (IACUC 2016-115). *Ebi3*^−/−^ C57BL/6 mice were purchased from The Jackson Laboratory (Bar Harbor, ME). We used three models to investigate the development of metastasis. For the syngeneic and orthotopic tumor models of mice, 1.5 × 10^5^ 4T1 cells were inoculated into the fat pad of 5- to 6-week-old BALB/c mice. Another syngeneic model developed by subcutaneously inoculating 1.5 × 10^5^ LLC cells into C57BL/6 or *Ebi3*^−/−^ C57BL/6 mice was also used in this study. For the orthotopic xenotransplantation model, 1 × 10^5^ SAS cells were implanted into the tongue of 6-week-old nude mice. After 4–5 weeks, metastatic lung nodules were examined in the 4T1 and LLC models, and metastatic lymph nodes in the xenograft SAS model were examined with a Xenogen IVIS spectrum system. To assay the metastatic colonization ability, cancer cells carrying luciferase vectors were suspended and injected into tail vein of mice. Lung metastases were measured by lung surface nodules, GFP-staining on lung paraffin sections, or ex vivo imaging with the IVIS system. Liposomal clodronate was intraperitoneally injected for systemic depletion of macrophages or intratracheally injected for depletion of pulmonary macrophages. Antibodies for intercepting the metastatic signals were also delivered intraperitoneally or intratracheally. In Fig. 8l, 50 mg mIl35 neutralizing antibodies (V1.4C4.22) or control IgG2b were delivered intratracheally since 2 weeks after tumor implantation, and total five doses of antibodies were given. Mice were killed at the end of 4th week. The primary tumors and lungs were harvested for analysis. To investigate the effect on colonization of the micrometastases, primary breast tumors of the 4T1 orthotopic model were surgically removed 3 weeks after inoculation, and the IVIS spectrum imaging was used to confirm the complete removal of tumors. After surgery, the mice were treated with antibodies, inhibitors, or control as indicated in each figure. Briefly, in Fig. 5f, 30 mg kg^−1^ ruxolitinib or normal saline was delivered through intraperitoneal injection every 2 days after surgery for 2 weeks. In Fig. 8q, the mice were injected intraperitoneally (i.p.) with 100 mg anti-IL-35 (V1.4C4.22), control mouse IgG2b, anti-Csf-1r, and control mouse IgG2a after surgery, then 50 mg every 3 days thereafter and a total of 4 doses were given. IVIS examination was performed at the end of 5th week. The recurrent/metastatic tumors were visualized by IVIS imaging, and the survival of mice was estimated using the Kaplan–Meier method.

### Isolation of TAMs from mouse and human tumors

TAMs were isolated from fresh primary and metastatic tumor samples. Briefly, the tissues were minced into small pieces and digested in Dulbecco’s modified Eagle’s medium containing 1.5 mg ml^−1^ collagenase IV (no. 9001-12-1, Thermo Fisher Scientific Inc., Waltham, MA) and 1.5 mg ml^−1^ hyaluronidase (no. H6254, Sigma-Aldrich, St. Louis, MO) at 37 °C for 1 h. The cells were subsequently filtered through a 200 mm cell strainer. The cells were then centrifuged at 700×*g* for 20 min, and Percoll (no. 17-5445-02, Sigma-Aldrich, St. Louis, MO) was used to separate the different layers of cells. Human TAMs were isolated by magnetic-activated cell sorting (MACS) using CD14 microbeads (no. 130-050-201, Miltenyi Biotec GmbH, Bergisch Gladbach, Germany), and mouse TAMs were sorted with the indicated markers shown in the figures using a BD FACSAria cell sorter (BD Biosciences, San Jose, CA). The sorting strategies were descried in Supplementary Figure 8 by using FlowJo (FlowJo, LLC, Ashland, OR)

### Patient samples

The study was approved by the Institutional Review Board (2016-07-001CC) of Taipei Veterans General Hospital. Four sets of patient samples were used in this study. The first set comprised paraffin-embedded samples of 10 matched pairs of primary-metastatic tumors of head and neck cancers. These samples were used for IHC analysis for GATA3, IL12Rβ2, STAT6, CD68, CD86, CD163, E-cadherin, and the proximity ligation assay for the detection of phosphorylated STAT6. The second set comprised 11 freshly isolated primary tumors (6 colon cancer, 4 head and neck cancer, and 1 gastric cancer) and 12 freshly isolated metastatic tumors (7 colon cancer, 4 head and neck cancer, and 1 gastric cancer). The samples were digested in Dulbecco’s modified Eagle’s medium containing 1.5 mg ml^−1^ collagenase IV and 1.5 mg ml^−1^ hyaluronidase immediately after harvesting from surgery, and MACS was used to sort the CD14^+^ TAMs for subsequent analysis. Human peripheral blood monocyte-derived macrophages (PMMs) were polarized from peripheral blood mononuclear cells (PBMCs) isolated from 10 healthy donors as a control for the study. The third set comprised 91 primary tumors of head and neck cancer patients (Supplementary Table 2). These samples were used for IHC analysis for IL12Rβ2 and to examine the correlation between the IL12Rβ2 expression level and cancer metastasis. The fourth set comprised paraffin-embedded samples of 37 paired primary-metastatic breast cancer samples. Among them, 4 pairs were from Taipei Veterans General Hospital, and 33 paired were purchased breast cancer tissue array (US Biomax, Inc. Rockville, MD). These samples were used for IHC analysis for IL12Rβ2 and to examine the correlation between IL12Rβ2 expression level and cancer metastasis.

### Quantitative RT-PCR

Quantitative RT-PCR (RT-qPCR) was performed using the StepOnePlus real-time PCR system (Applied Biosystems Inc., Foster City, CA). The primer sequences used for real-time PCR are listed in Supplementary Data 5.

### Western blot

Cells were lysed in RIPA (50 mM Tris HCl, pH 7.4; 150 mM NaCl; 1 mM EDTA; 1 % NP-40; 0.1 % SDS; 0.5% sodium deoxycholate) buffer with 1× proteinase inhibitor from Roche (Mannheim, Germany) and incubated on ice for 20 min. Next, cell lysate were transferred into new eppendorf and vortex for 1 minute. After vortex, the lysate were centrifuged at 20,000×*g* for 10 min then collected the supernatants. The quantity was determined with an infinite M200 (Tecan, Swizerland) using BCA protein assay (Thermo Scientific Pierce™ BCA protein assay, Waltham, MA). All samples were diluted into equal protein concentration by adding proper volume of RIPA buffer. In order to break protein structure, 6× sample buffer was added into each sample and mixed. Mixtures were heated at 95 °C for 5 min. Denatured proteins were loaded in 6–12% SDS-PAGE gels for separating proteins with running buffer. The PVDF membrane from Millipore (Billerica, MA) was used to transfer protein sample from gels to membrane. The transfer system was used at 300 mA on ice for 2 h. Membranes containing denatured proteins were blocked in TBST with 5% skim milk at room temperature for 1 h. After blocking by milk, all membranes were washed for three times for 10 min each with TBST. Then, all membranes were incubated with particular primary antibodies at 4 °C overnight. The membrane was washed in TBST and incubated with secondary antibodies in 5% skim milk for 1 h at room temperature. The membrane was washed in TBST again, then incubated with ECL from Millipore (Billerica, MA). The results were measured using a GE LAS-4000 (GE Healthcare Inc., Marlborough, MA). The antibodies used in the experiments are detailed in Supplementary Data 6. The blots were presented as cropped with at least one marker for indicating the molecular weight position. The uncropped scans of all blots are shown in Supplementary Figure 9.

### Macrophage depletion

Clodronate and phosphate buffer solution liposomes were purchased from ClodronateLiposomes.org (Haarlem, Netherlands). The concentration of clodronate in the liposome formulation was 5 mg ml^−1^. A single dose of liposomal clodronate was administered via intraperitoneal (1 mg per mouse) or intratracheal (0.5 mg per mouse) injection at the indicated times.

### T cell-suppression assay

For the suppression of T cells, 1 × 10^5^ CD4 cells from splenocytes of BALB/c mice isolated by magnetic cell sorting (no. 130-045-101, Miltenyi Biotec GmbH, Bergisch Gladbach, Germany) were seeded and stimulated with anti-CD3/28 beads (no. 11131D, Thermo Fisher Scientific Inc., Waltham, MA) and added to 5 × 10^4^ BMDMs or sorted TAMs per well in a 96-well plate. After 24 h, the proliferation of T cells was analyzed by the cell proliferation assay.

### Endothelial cell capillary formation assay

Conditioned medium (obtained from BMDMs, sorted TAMs, PMDM, M1 macrophages, or M2 macrophages) was used to resuspend 5 × 10^4^ HUVECs, which were then seeded directly on Matrigel. After 12 h, capillary formation was analyzed and quantified by measuring the number of branches.

### Chromatin immunoprecipitation

For the chromatin immunoprecipitation (ChIP) assays, chromosomal DNA fragments were prepared according to the manufacturer’s instructions (Thermo Scientific Pierce™ Magnetic ChIP Kit, Waltham, MA). Briefly, the lysates were incubated with IgG (2 μg) or STAT6-specific antibodies (5 μg). Quantitative PCR (qPCR) assay was performed to detect the relative enrichment. The primers for amplifying the interested region used in the experiments are listed in Supplementary Data 5, and the antibodies used in the experiments are detailed in Supplementary Data 6.

### Reporter assay

The T-Pro transfection reagent (cat. no. JT98-T001, Taiwan) was used for transient transfections. The constructs containing the wild-type (WT) or STAT6 binding-site mutated (MUT) proximal promoter region of *GATA3* were used in the study (Fig. 6i). The STAT6 binding motif (TTC(N)2-4GAA) is located at −383 upstream to the transcription start site. For the reporter assay, 0.2 μg of the full-length or mutated reporter constructs, 0.2 μg of the pCMV-β-gal plasmid, and 1 μg of STAT6 expression vector/control vector plasmid were cotransfected into HEK-293T cells (1.5 × 10^5^ cells) and incubated for 48 h before the luciferase activity was measured. The result is presented as the fold change of luciferase activity/β-galactosidase activity.

### Proximity ligation assay

The proximity ligation assay was used to investigate the proximity of the epitopes recognized by the anti-STAT6 and anti-Tyrosine phosphorylation-specific antibodies, which represent the expression of phosphorylated STAT6 in cancer cells. The experiment was performed according to the manufacturer’s instructions. Briefly, after incubation with primary antibodies, the corresponding DuoLink® In Situ PLA probes (OLINK Bioscience, Uppsala, Sweden) were applied for 1 h at 37 °C as recommended. Subsequent ligations and detections using the DuoLink® In Situ Detection Reagents Red Kit (OLINK Bioscience, Uppsala, Sweden) were performed. Blocking, antibody hybridization, proximity ligation, and detection were performed according to the manufacturer’s recommendations. The fluorescence images were captured using an Olympus FluoView FV10i Laser confocal microscope (Olympus Corporation, Tokyo, Japan). Images were collected sequentially on a confocal laser scanning microscope (Olympus UPLSAPO 60XO, NA 1.35) and analyzed by Olympus FV10-ASW Version 3.0 Software. The results were quantified using MetaMorph® Microscopy Automation & Image Analysis Software.

### Ingenuity pathway analysis

Pathway and global functional analyses were performed using Ingenuity Pathway Analysis (IPA; Ingenuity® Systems, www.ingenuity.com). The dataset containing gene identifiers and corresponding expression values was uploaded, and each gene was mapped using the Ingenuity Pathways Knowledge Base (IPKB). Genes from the datasets that are associated with biological functions in the IPKB and that met the *p*-value cutoff of 0.05 were used to build the interactome.

### Analyses of public databases and GSEA

Survival curves of gene expression in lung cancer patients were obtained from the website (http://kmplot.com/analysis/). GSEA was performed using the JAVA program (http://www.broadinstitute.org/gsea). The core EMT gene signatures^45^ were used to integrate the transcriptome changes in M1- and M2-CM-treated A549 cells.

### Preparation of human monocytes

Peripheral mononuclear cells were isolated from the blood of healthy donors by standard density gradient centrifugation with Ficoll-Paque (Amersham Biosciences, Inc., Piscataway, NJ). CD14^+^ cells were subsequently purified from peripheral mononuclear cells by high-gradient magnetic sorting using anti-CD14 microbeads (No. 130-050-201, Miltenyi Biotec GmbH, Bergisch Gladbach, Germany). The CD14^+^ monocytes were cultured in RPMI-1640 medium (Life Technologies, Inc., Gaithersburg, MD) supplemented with hM-CSF for 5 days for the polarization of M0 macrophages. Fresh medium supplemented with hM-CSF (20 ng ml^−1^) (No. 30025, PeproTech, Inc., Rocky Hill, NJ) was added on day 3.

### Macrophage polarization and conditioned medium collection

The M0 macrophages were polarized into M1 or M2 macrophages by adding 1 µg ml^−1^ lipopolysaccharide (LPS) plus 20 ng ml^−1^ interferon-γ (IFN-γ) or 20 ng ml^−1^ interleukin-4 (IL-4) plus 0.1 µM dexamethasone in 5% FBS RPMI-1460 medium, respectively^46–48^. After 48 h, the media of the polarized macrophages were changed into fresh media for another 48 h, which served as the different M1- and M2-conditioned media.

### Flow cytometry

Cells were harvested and washed twice with PBS. The cells were then incubated with primary antibodies (listed in Supplementary Data 6) for 1 h at 4 °C and then with secondary antibodies for 30 min at 4 °C. The stained cells were analyzed on a Cytomics^TM^ FC500 Flow Cytometry apparatus (Beckman Coulter, Inc., Brea, CA) and BD FACSCalibur (BD Biosciences, San Jose, CA) using Cytomics CXP Analysis software (Beckman Coulter, Inc., Brea, CA) and BD CellQuest™ Pro Software (BD Biosciences, San Jose, CA), respectively.

### Enzyme-linked immunosorbent assay (ELISA)

Conditioned media were assayed using IL-35 ELISA kits (cat. no. 440508 and 439508 BioLegend, Inc.). Sorted TAMs or polarized macrophages were seeded and cultured for 24 h. Supernatants were collected after centrifugation, and IL-35 was measured by ELISA.

### Immunofluorescence

The cells were seeded on poly-l-lysine-coated slides, fixed with 4% paraformaldehyde, and permeabilized with 0.5% Triton X-100. DAPI was used for nuclear staining. The images were captured using an Olympus FluoView FV10i laser scanning confocal microscope (Olympus Corporation, Tokyo, Japan) equipped with a ×60 oil objective (Olympus UPLSAPO 60XO, NA 1.35). Images were collected sequentially using the confocal laser scanning microscope and analyzed using Olympus FV10-ASW Version 3.0 Software. The antibodies used in the staining are listed in Supplementary Data 6.

### Immunohistochemistry

The samples were following the steps of deparaffinization, rehydration, antigen retrieval (10 mM sodium citrate buffer, pH 6.0), permeabilization, antibody hybridization and visualization using Novocastra Reagents (Leica Biosystems, Germany). For immunohistochemical grading, the intensity of GATA3 and IL12RB2 were defined as 0, 1+, 2+, or 3+. The immunoscore (H score) was defined by the intensity (0–3+) multiplied by the expression percentage (0–100) for each sample. The slides were independently scored by two individuals. The antibodies used in the staining are listed in Supplementary Data 6.

### Cell viability and proliferation assay

For the cell viability assay, 1 × 10^4^ cells were seeded per well in a 96-well plate and incubated overnight and then treated with various concentrations of reagents. After 24 h, the growth medium was discarded, and MTT assay solution was added for 1 h at 37 °C. Newly formed mitochondrial MTT crystals were dissolved with dimethyl sulfoxide, and the absorbance was read using a microplate reader. For the cell proliferation assay, a BrdU ELISA assay (Roche Applied Science) was used to measure the rate of DNA synthesis.

### Cell migration assays

Cell migration was evaluated using Transwells with 8-μm-filter-membrane-containing upper chambers (Greiner Bio-One). Cells (5 × 10^4^) suspended in 100 μl of 0.5% FBS culture medium were applied to the upper chamber, and 600 μl of 10% FBS medium was added to the lower chamber. After 24 h, the membranes were fixed with 4% PFA and then stained for visualization.

### Endothelial permeability assay

Endothelial permeability was evaluated using Transwells with 8-μm-filter-membrane-containing upper chambers (Greiner Bio-One). On the first day, human umbilical vein endothelial cells (HUVEC) were plated at a density of 2 × 10^5^ cells per Transwell for 24 h. On the second day, the conditioned media were treated with HUVECs for 24 h. Endothelial permeability was determined by measuring the passage of FITC_−_labeled dextran (1 mg ml^−1^ of fluorescein isothiocyanate-dextran, molecular mass: 40 kDa; Sigma-Aldrich) through the HUVEC monolayer. After 30 min of treatment, 100 μl was collected from the lower compartment, and the fluorescence was measured.

### Cell–cell adhesion assay

Human bronchial epithelial cells (NL20) were plated at a density of 1.5 × 10^5^ cells per well in the Nunc™ Lab-Tek™ II Chamber Slide™ System (Cat No. 155384, Thermo Fisher Scientific Inc., Waltham, MA) for 24 h. On the second day, conditioned medium-treated A549 cells were plated in a chamber slide for 10 min, and then the chamber slide was washed with PBS. Fluorescence-labeled A549 cells were measured using an Olympus FluoView FV10i laser scanning confocal microscope (Olympus Corporation, Tokyo, Japan).

### Statistical analysis

A two-tailed Student’s *t*-test was used to compare continuous variables between two groups. The *χ*^2^ test was applied to compare nondichotomous variables. The Kolmogorov–Smirnov test was used for GSEA. Kaplan–Meier estimation and the log-rank test were used to compare survival between patient groups. All statistical data were derived from at least two independent biological replicates, and each experiment contained at least two technical replicates. *p*-Values < 0.05 were considered significant.

## Electronic supplementary material

Supplementary Information

Description of additional supplementary files

Supplementary Data 1

Supplementary Data 2

Supplementary Data 3

Supplementary Data 4

Supplementary Data 5

Supplementary Data 6

## Data Availability

All relevant data are available from the corresponding author upon reasonable request. The datasets for the cDNA microarray for the conditioned medium-treated A549 cells were deposited at the Gene Expression Omnibus (GEO) under accession number GSE96943. The datasets for the cDNA microarray for the pTAMs vs. mTAMs (BMDM as a control) from the 4T1 mouse model were deposited at the Gene Expression Omnibus (GEO) under accession number GSE96944. The URL for Supplementary Figure 7f that analyzed the survival of *IL12RB2* expression in lung cancer patient samples from a publically available dataset is [http://www.kmplot.com/analysis/index.php?p=service&start=1].
