# Supplementary material for: Macrophage-secreted interleukin-35 regulates cancer cell plasticity to facilitate metastatic colonization
Source: Nat Commun. 2018 Sep 14;9:3763. doi: 10.1038/s41467-018-06268-0 (PMC6138674; doi:10.1038/s41467-018-06268-0)
Supplement: Supplementary file 1 — Supplementary Information [file 41467_2018_6268_MOESM1_ESM.pdf]

Supplementary information

Macrophage-secreted Interleukin-35 Regulates Cancer Cell Plasticity to Facilitate Metastatic Colonization

Lee et al.

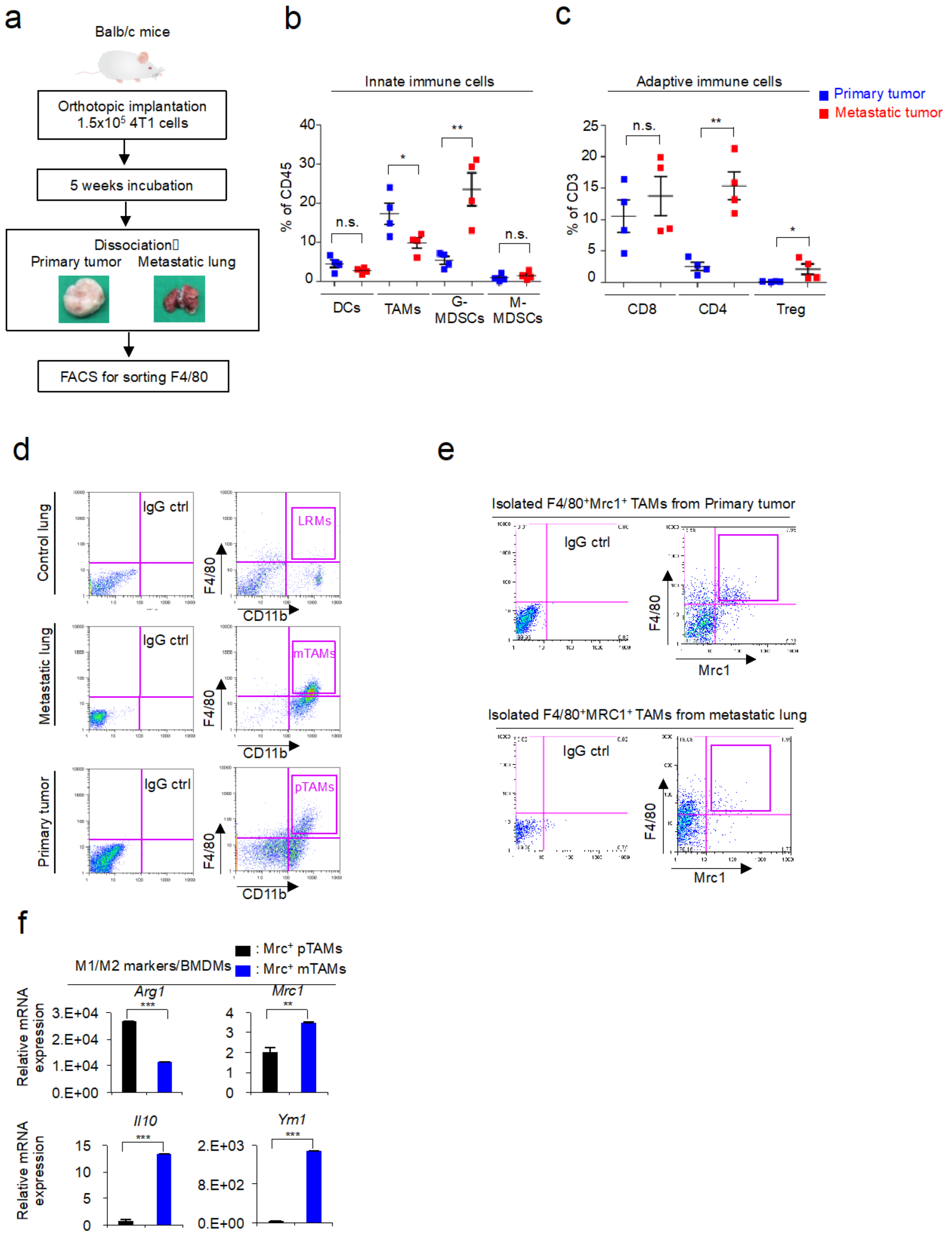

Supplementary Fig 1. Different phenotypes of macrophages in primary and metastatic tumors. **a** Schema for presenting the procedure of isolation of CD11b$^+$ F4/80$^+$ macrophages from primary tumors and lungs of the 4T1 orthotopic tumor model. **b-c** Relative amount of adaptive immune cells (CD11b$^+$F4/80$^+$

macrophages, $CD11b^+Gr1^+$ MDSCs, and $CD11c^+MHCII^+$ DCs) and innate immune cells ($CD4^+$ T cells $CD8^+$ T cells, and $CD4^+Foxp3^+$ Treg) in primary and metastatic tumor site from 4 different animals (n=4). Granulocytic MDSCs, G-MDSCs; monocytic MDSCs, M-MDSCs. n.s., non-significance. **d** Representative result of the flow cytometry for sorting $CD11b^+F4/80^+$ macrophages from the lungs of normal mice, lungs of metastatic tumor-bearing mice, and primary tumors. IgG was a control for flow cytometry. LRMs, lung-resident macrophages. **e** Representative result of the flow cytometry for sorting $CD11b^+F4/80^+Mrc1^+$ macrophages from the lungs bearing metastatic tumors and from primary tumors. IgG was a control for flow cytometry. **f** RT-qPCR for analyzing the expression of M2 markers (*Arg1*, *Mrc1*, *Il10*, and *Ym1*) of $CD11b^+F4/80^+Mrc1^+$ cells from the lungs bearing metastatic tumors and primary tumors. The data were normalized to BMDM (n=3) from healthy mice. n=3 (triplicated RT-qPCR from the same mouse). For the panels in supplementary Fig. 1, Data represent mean ± S.E.M. $^{*}p < 0.05$, $^{**}p < 0.01$, $^{***}p < 0.001$. Statistical analysis: Student's *t*-test (b, c, f).

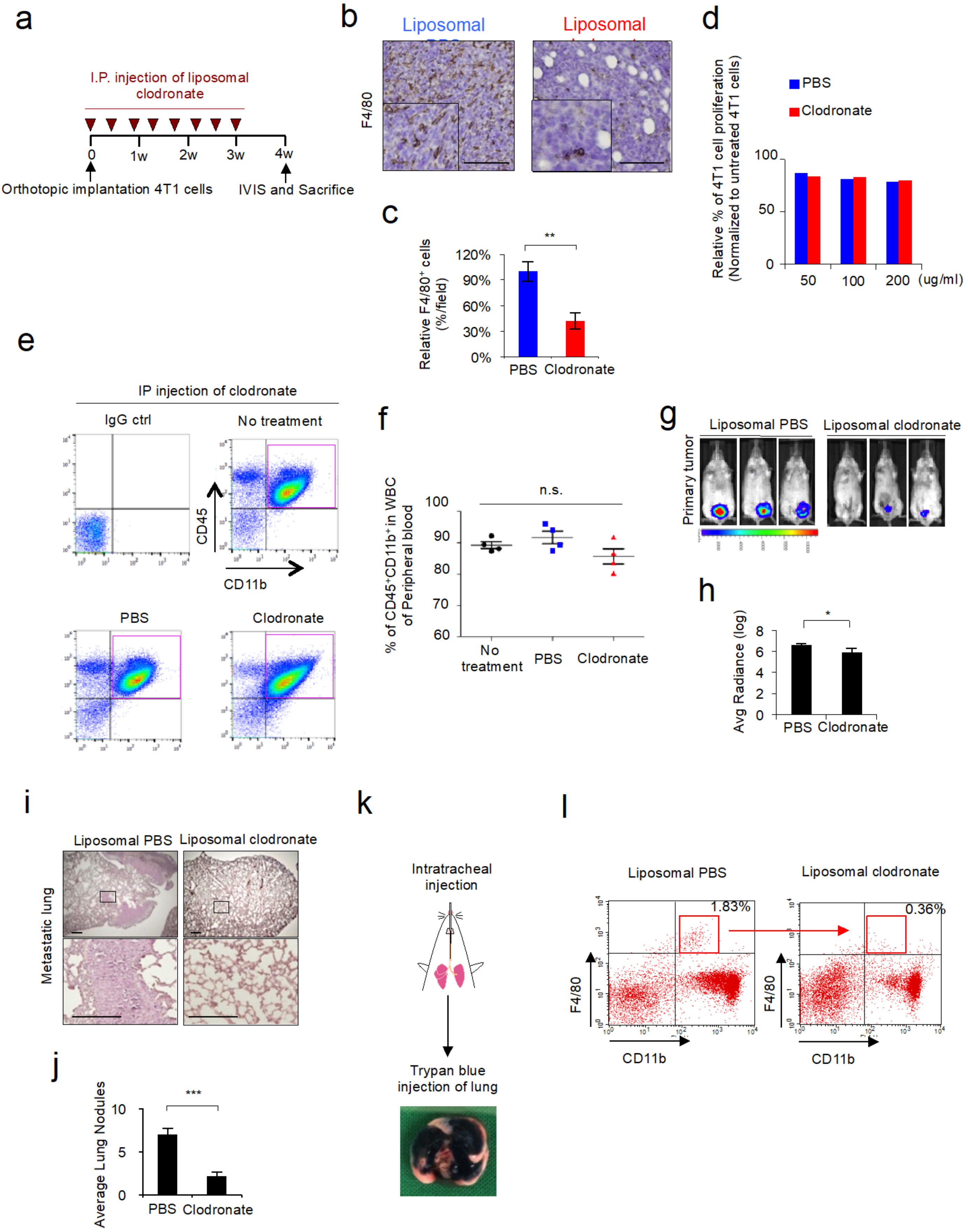
a
I.P. injection of liposomal clodronate
0
1w
2w
3w
4w
Orthotopic implantation 4T1 cells
IVIS and Sacrifice
b
Liposomal
Liposomal
F4/80
c
Relative F4/80+ cells (%/field)
120%
90%
60%
30%
0%
**
PBS
Clodronate
d
Relative % of 4T1 cell proliferation (Normalized to untreated 4T1 cells)
PBS
Clodronate
100
50
0
50
100
200
(ug/ml)
e
IP injection of clodronate
IgG ctrl
No treatment
CD45
CD11b
PBS
Clodronate
f
% of CD45+CD11b+ in WBC of Peripheral blood
n.s.
100
90
80
70
60
No treatment
PBS
Clodronate
g
Liposomal PBS
Liposomal clodronate
Primary tumor
h
Avg Radiance (log)
*
8
6
4
2
0
PBS
Clodronate
i
Liposomal PBS
Liposomal clodronate
Metastatic lung
j
Average Lung Nodules
***
10
5
0
PBS
Clodronate
k
Intratracheal injection
Trypan blue injection of lung
l
Liposomal PBS
Liposomal clodronate
1.83%
0.36%
F4/80
CD11b

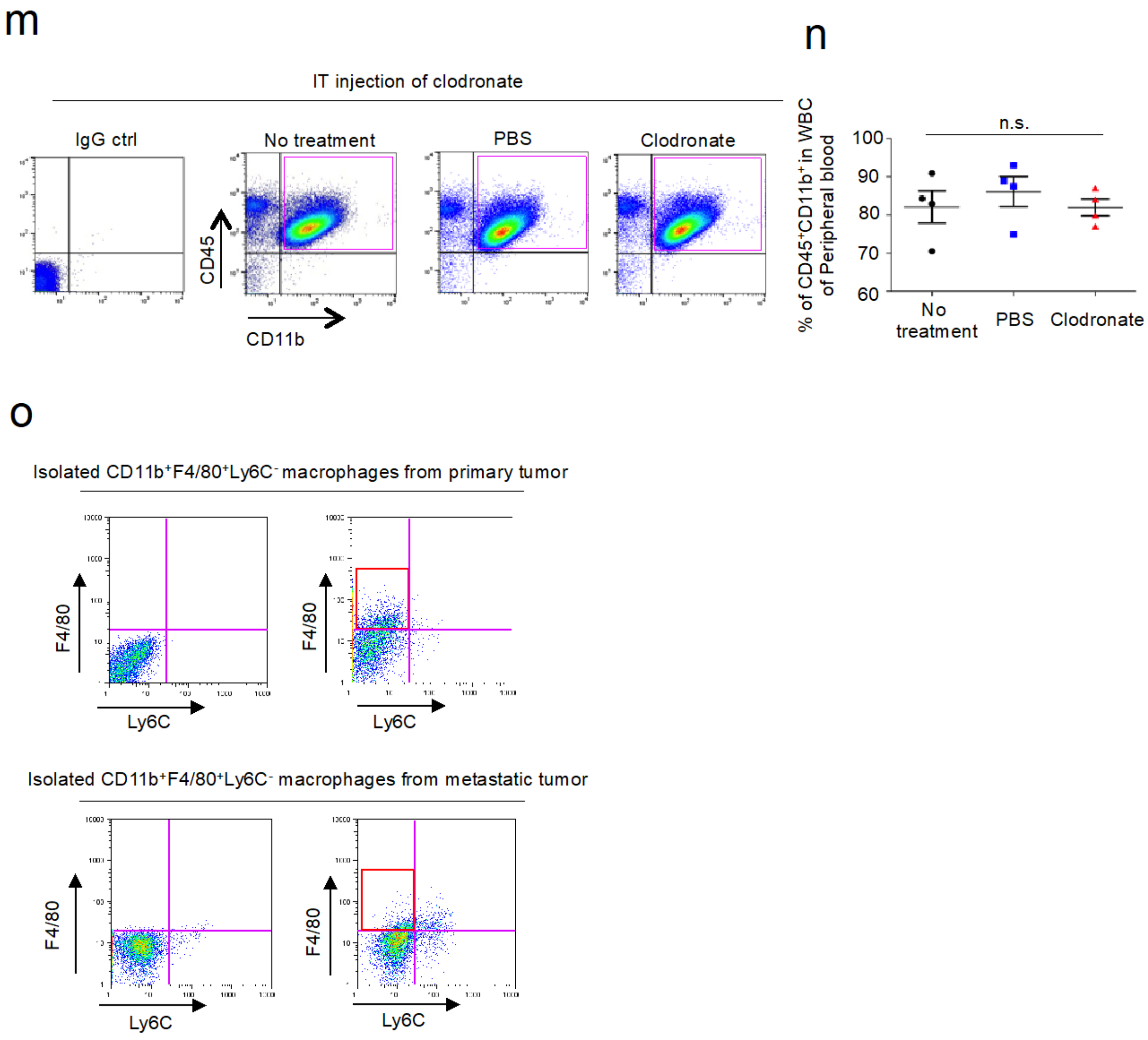

Supplementary Fig 2. Significance of TAMs in metastatic colonization. **a** Schema of orthotopic tumor experiments in mice receiving systemic depletion of macrophages by intraperitoneal injection of liposomal chlodronate. **b** IHC of F4/80 for showing the recruitments of macrophages in orthotopic tumors after liposomal clodronate or PBS treatment. Scale bar, 100 μm (low power field). **c** Quantification of F4/80$^+$ macrophage in orthotopic tumors after liposomal clodronate or control PBS treatment. n=6 for each group **d** MTT assay for analyzing the viability of 4T1 cells under PBS or liposomal clodronate treatment (50, 100, and 200 μg/ml for 24 hr). n=2 independent experiments (each experiment contains 4 technical replicates). **e** Representative result of the flow cytometry for sorting CD45$^+$CD11b$^+$ circulating monocyte/macrophages in white blood cells under liposomal clodronate treatment via intraperitoneal injection. IgG was a control for flow cytometry. **f** Quantification of CD45$^+$CD11b$^+$ circulating monocyte/macrophages in white blood cells under liposomal clodronate treatment via intraperitoneal injection. n=4. **g** Images for bioluminescent signals after 4 weeks of 4T1 inoculation. **h** Quantification of bioluminescent imaging. **i** Representative result of the hematoxylin & eosin stain of the lungs of mice. Scale bar, 200 μm. **j** Quantification of metastatic lung nodules 4 weeks after 4T1 inoculation with/without systemic depletion of macrophages. n=6 for each group. **k** Schema of intratracheal injection. Trypan blue was an indicator for demonstrating the successful delivery of the cargo into lungs. **l** Flow cytometric analysis of CD11b and F4/80 for confirming the successful depletion of pulmonary macrophages by intratracheal injection of liposomal chlodronate. **m** Representative result of the flow cytometry for sorting CD45$^+$CD11b$^+$ circulating monocyte/macrophages in white blood cells under liposomal clodronate treatment via intratracheal injection. IgG was a control for flow cytometry. **n** Quantification of CD45$^+$CD11b$^+$ circulating monocyte/macrophages in white blood cells under liposomal

clodronate treatment via intratracheal injection. n=4. **o** Representative result of flow cytometry for detecting $CD11b^+F4/80^+Ly6C^-$ macrophages in the primary tumors and lungs of the metastatic tumor-bearing mice. For the panels in supplementary Fig. 2, Data represent mean ± S.E.M. $^*p < 0.05$, $^{**}p < 0.01$, $^{***}p < 0.001$. Statistical analysis: Student's *t*-test (c, f, h, j, n).

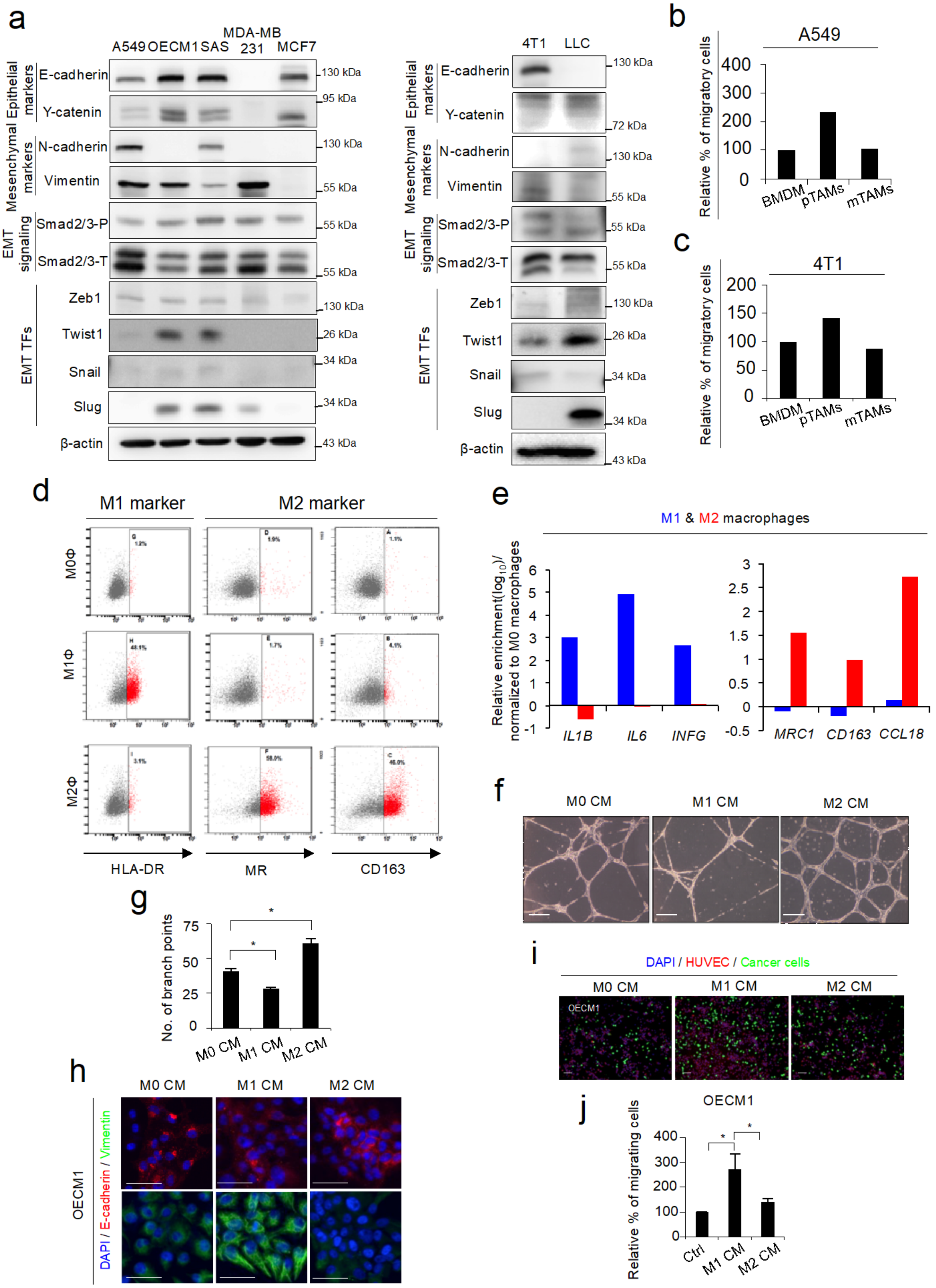
a
A549
OECM1
SAS
MDA-MB 231
MCF7
Epithelial markers
E-cadherin
Y-catenin
Mesenchymal markers
N-cadherin
Vimentin
EMT signaling
Smad2/3-P
Smad2/3-T
EMT TFs
Zeb1
Twist1
Snail
Slug
β-actin
130 kDa
95 kDa
130 kDa
55 kDa
55 kDa
55 kDa
130 kDa
26 kDa
34 kDa
34 kDa
43 kDa
4T1
LLC
72 kDa
b
A549
Relative % of migratory cells
400
300
200
100
0
BMDM
pTAMs
mTAMs
c
4T1
200
150
100
50
0
d
M1 marker
M2 marker
M0Φ
M1Φ
M2Φ
HLA-DR
MR
CD163
e
M1 & M2 macrophages
Relative enrichment(log10)/ normalized to M0 macrophages
IL1B
IL6
INFG
MRC1
CD163
CCL18
f
M0 CM
M1 CM
M2 CM
g
No. of branch points
75
50
25
0
*
M0 CM
M1 CM
M2 CM
h
OECM1
DAPI / E-cadherin / Vimentin
i
DAPI / HUVEC / Cancer cells
OECM1
j
OECM1
Relative % of migrating cells
400
300
200
100
0
Ctrl
M1 CM
M2 CM

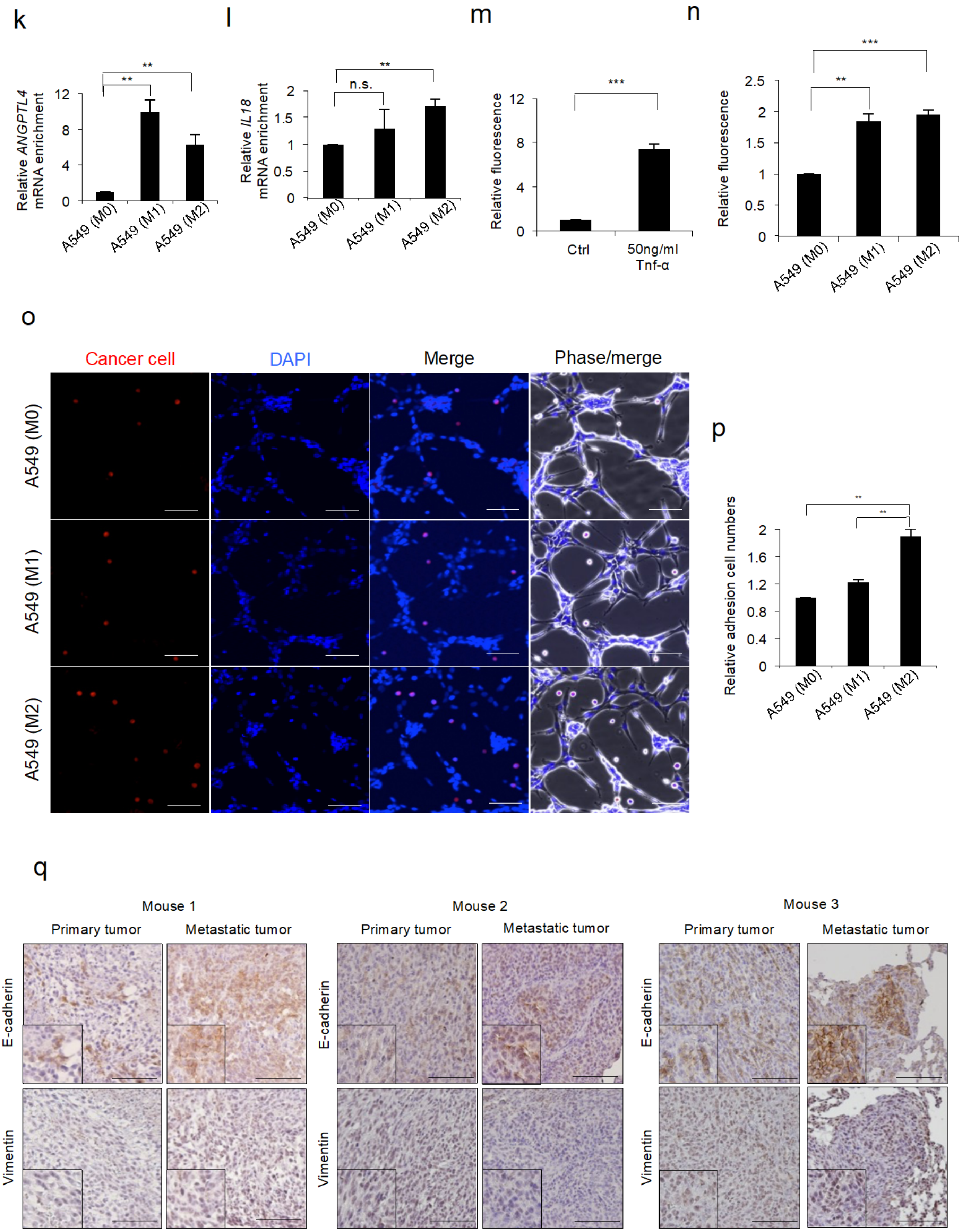
k
Relative ANGPTL4 mRNA enrichment
A549 (M0)
A549 (M1)
A549 (M2)
l
Relative IL18 mRNA enrichment
n.s.
m
Relative fluorescence
Ctrl
50ng/ml Tnf-α
n
o
Cancer cell
DAPI
Merge
Phase/merge
p
Relative adhesion cell numbers
q
Mouse 1
Mouse 2
Mouse 3
Primary tumor
Metastatic tumor
E-cadherin
Vimentin

r

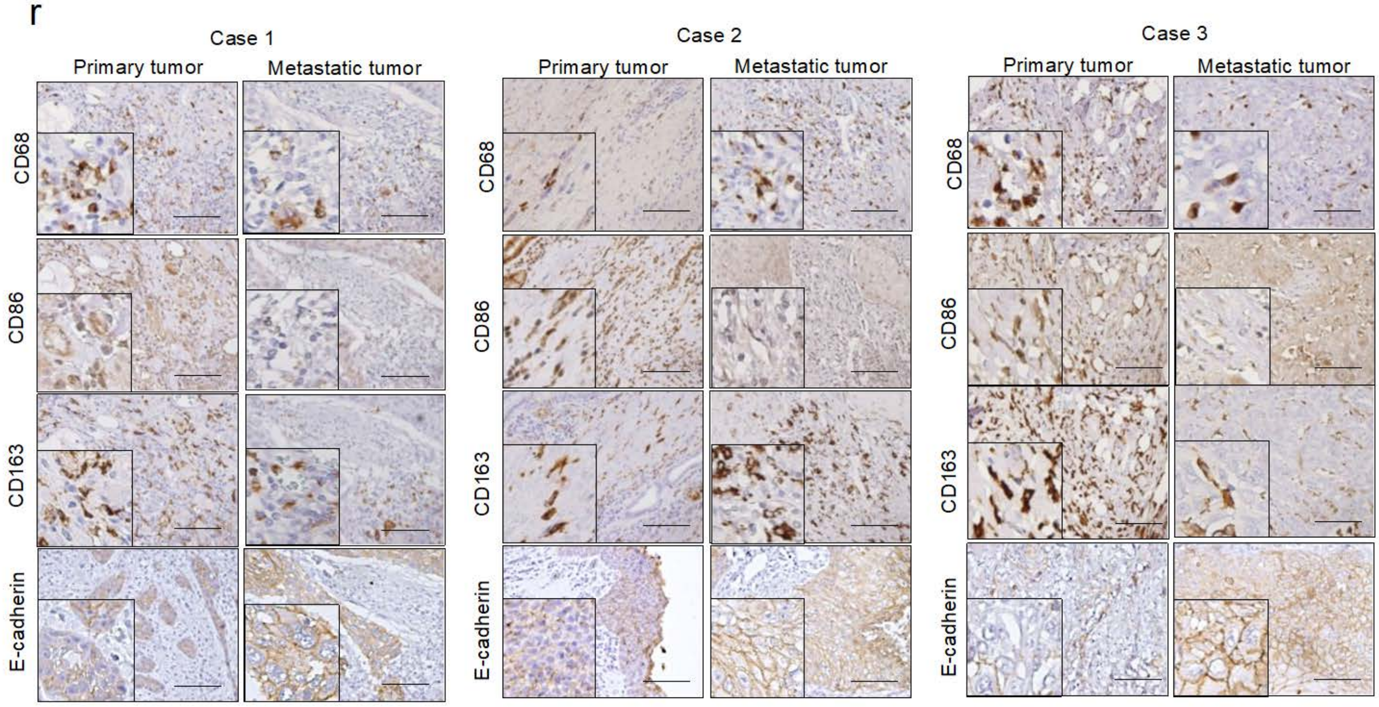

Supplementary Fig 3. The impacts of macrophages on cancer epithelial plasticity. **a** Expressions of the EMT-related molecules in cell lines used in this study. **b-c** Transwell migration assay of A549 and 4T1 incubated with different conditioned media (CM) for 48 hr. n=2 independent experiments (the data of each experiment was the mean value of quantification of at least three randomly-selected fields). BMDM, bone marrow-derived macrophages; pTAM and mTAM: $CD11b^{+}F4/80^{+}$ macrophages from primary and metastatic tumors of 4T1-BALB/c orthotopic model, respectively. **d** Flow cytometry for M1 (HLA-DR) and M2 (MR and CD163) markers in polarized macrophages from human $CD14^{+}$ monocytes. **e** RT-qPCR of M1 and M2 markers in polarized macrophages from human $CD14^{+}$ monocyte. n=2 independent experiments (each contains 2 technical replicates). **f** Representative images of tube formation assay of HUVEC co-cultured with M0 (resting macrophages), M1, and M2 CM for 12 hr. Scale bar, 50 μm. **g** Quantification of (f). n=3 independent experiments (the data of each experiment was the mean value of quantification of three randomly-selected fields). Scale bar, 50 μm. **h** Immunofluorescent staining of E-cadherin (red) and vimentin (green) in OECM1 incubated with the indicated CM for 48 hr. Blue, nuclei. Scale bar, 100 μm. **i** Transendothelial migration assay of OECM1 upon indicated CM treatment. Scale bar, 100 μm. **j** Quantification of cancer cell. n=3 independent experiments (the data of each experiment was the mean value of quantification of at least three randomly-selected fields). **k-l** RT-qPCR of *ANGPTL4* and *IL18* upon indicated CM treatment for 24 hr. n=3 independent experiments (each contains 2 technical replicates). **m-n** Endothelial permeability assay for HUVEC upon Tnf-α or indicated CM treatment for 24 hr. Tnf-α, a positive control for HUVEC permeability. n=3 independent experiments (each contains 2 technical replicates). **o** Cell adhesion assay for the adhesive ability of A549 upon treatment with indicated CM for 48 hr to NL20 cells. Red, A549; blue, nuclei. Scale bar, 50 μm. **p** Quantification of adhesive A549 cells. n=3 independent experiments (the data of each experiment was the mean value of quantification of ten randomly-selected fields). **q** IHC of E-cadherin and vimentin in paired primary-metastatic tumor from 3 4T1-BALB/c orthotopic mice. Magnification in the inset. Scale bar, 100 μm. **r** IHC of CD68, CD86, CD163, and E-cadherin in paired primary-metastatic tumor from three head and neck cancer patients. Scale bar, 100 μm. Data represent mean ± S.E.M. $^{*}p < 0.05$, $^{**}p < 0.01$, $^{***}p < 0.001$. Statistical analysis: Student's *t*-test (g, j, k, l, m, n, p).

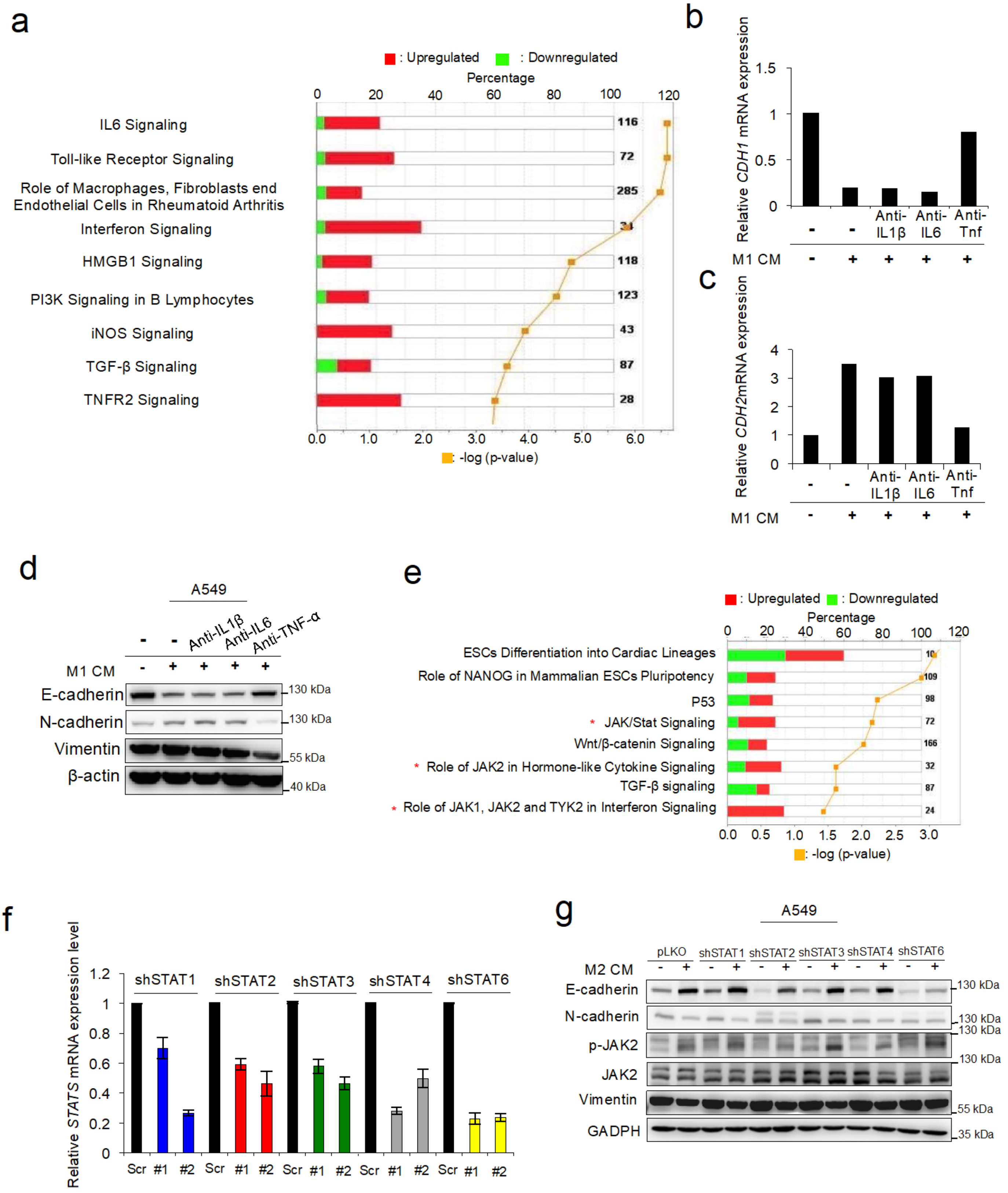
a
: Upregulated : Downregulated
Percentage
IL6 Signaling
Toll-like Receptor Signaling
Role of Macrophages, Fibroblasts end Endothelial Cells in Rheumatoid Arthritis
Interferon Signaling
HMGB1 Signaling
PI3K Signaling in B Lymphocytes
iNOS Signaling
TGF-β Signaling
TNFR2 Signaling
116
72
285
118
123
43
87
28
: -log (p-value)
b
Relative CDH1 mRNA expression
Anti-IL1β
Anti-IL6
Anti-Tnf
M1 CM
c
Relative CDH2mRNA expression
Anti-IL1β
Anti-IL6
Anti-Tnf
M1 CM
d
A549
Anti-IL1β
Anti-IL6
Anti-TNF-α
M1 CM
E-cadherin
N-cadherin
Vimentin
β-actin
130 kDa
130 kDa
55 kDa
40 kDa
e
: Upregulated : Downregulated
Percentage
ESCs Differentiation into Cardiac Lineages
Role of NANOG in Mammalian ESCs Pluripotency
P53
* JAK/Stat Signaling
Wnt/β-catenin Signaling
* Role of JAK2 in Hormone-like Cytokine Signaling
TGF-β signaling
* Role of JAK1, JAK2 and TYK2 in Interferon Signaling
109
98
72
166
32
87
24
: -log (p-value)
f
Relative STATS mRNA expression level
shSTAT1
shSTAT2
shSTAT3
shSTAT4
shSTAT6
Scr #1 #2 Scr #1 #2 Scr #1 #2 Scr #1 #2 Scr #1 #2
g
A549
pLKO shSTAT1 shSTAT2 shSTAT3 shSTAT4 shSTAT6
M2 CM
E-cadherin
N-cadherin
p-JAK2
JAK2
Vimentin
GADPH
130 kDa
130 kDa
130 kDa
130 kDa
55 kDa
35 kDa

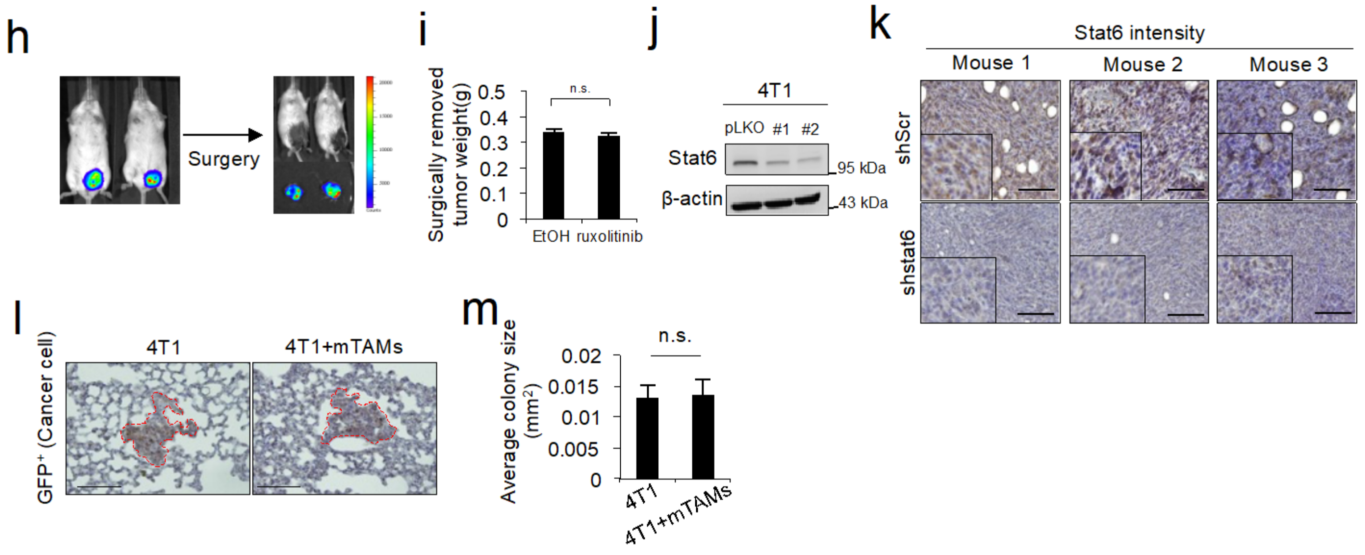

Supplementary Fig 4. Mechanisms of macrophages-regulated cancer epithelial plasticity. **a** Ingenuity Pathway Analysis (IPA) of the cDNA microarray data from A549 treated with M1 conditioned media (CM) for 48 hr. The histogram shows the majorly involved signal pathways in M1 CM-treated A549 cells. **b-c** RT-qPCR of analyzing the expression of *CDH1* and *CDH2* in M1 CM-treated A549 under the indicated neutralizing antibody treatment. n=2 independent experiments (each experiment contains 2 technical replicates). **d** Western blots of the EMT markers in M1 CM-treated A549 under the indicated neutralizing antibody treatment. **e** IPA of the cDNA microarray data obtained from A549 treated with M2 CM for 48 hr. The histogram shows the majorly involved signaling pathways in A549. **f** RT-qPCR for confirming the knockdown efficiency of the *STAT1, 2, 3, 4,* and *6* in A549 receiving shRNA against different STAT family members or a control sequence (pLKO). n=3 independent experiments (each experiment contains 2 technical replicates). **g** Western blots for total and phosphorylated JAK2 and EMT markers (E-cadherin, N-cadherin, vimentin) in A549 receiving shRNA against different family members of STAT (clone #1) under M2 CM treatment for 48 hr. **h** Representative images of surgically removed primary tumors in 4T1-BALB/c orthotopic model 3 weeks after tumor cells inoculation. **i** Quantification of tumor weight. n.s., non-significance. n=6 for each group. **j** Western blots for confirming the Stat6 knockdown efficiency by two independent sequences in 4T1 cells. **k** IHC of Stat6 in primary tumors of 4T1-BALB/c orthotopic model. The samples were harvested 4 weeks after tumor inoculation. The magnified images were shown as inset of each panel. Scale bar, 100 μm (low power field). **l** Representative images of IHC for GFP of lungs from BALB/c mice receiving intravenous injection of 4T1 cells with/without mTAMs (harvested from 4T1-BALB/c syngeneic orthotopic model). Scale bar, 100 μm. **m** Quantification of average GPF-positive colonies from 5 paraffin-embedded lung section. n.s., non-significance. n=5 for each group. For the panels in supplementary Fig. 4, Data represent mean ± S.E.M. $^{*}p < 0.05$, $^{**}p < 0.01$, $^{***}p < 0.001$. Statistical analysis: Student's *t*-test (i, m).

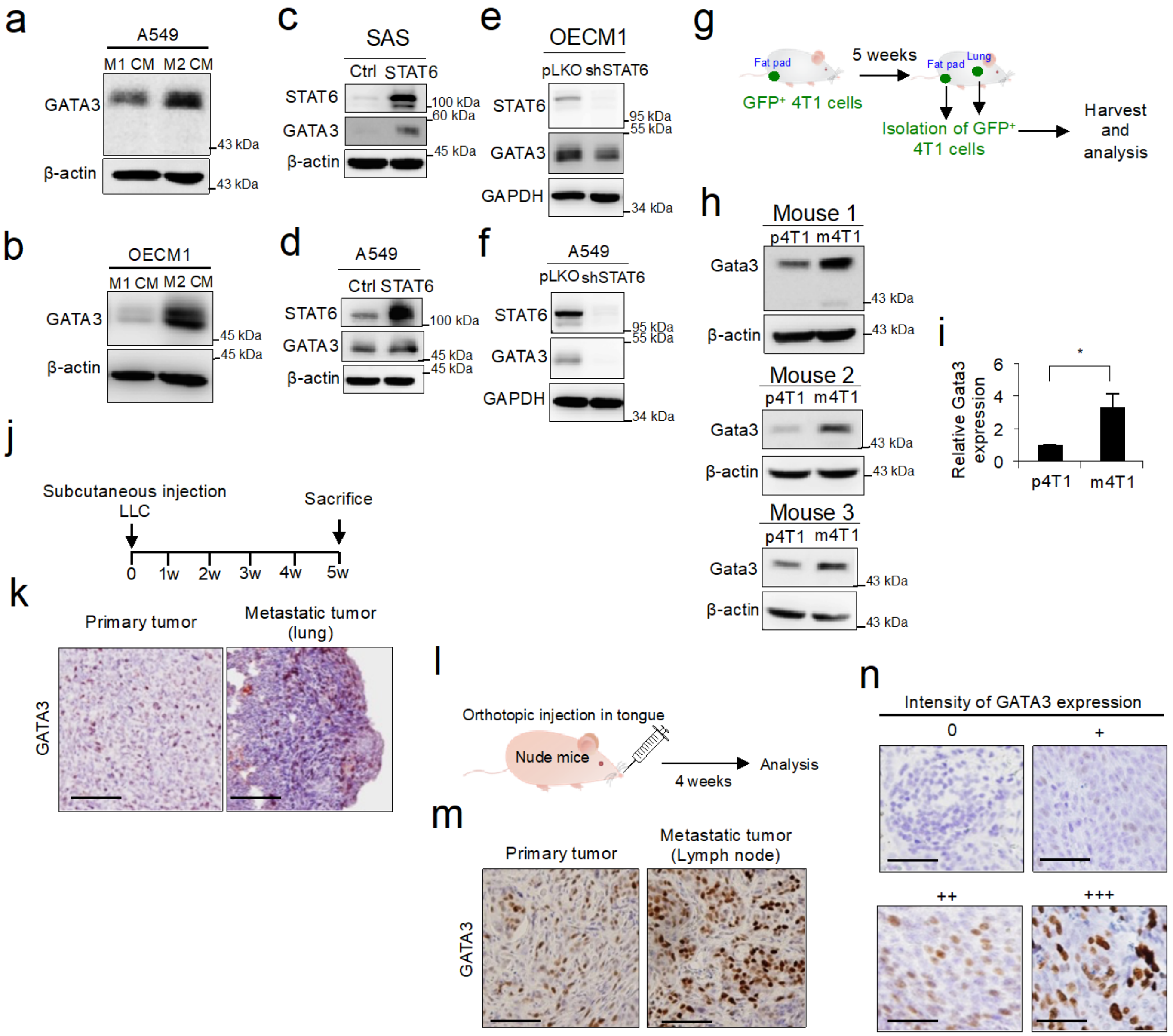

Supplementary Fig 5. Expression of GATA3 in metastatic tumors. **a-b** Western blots of GATA3 in A549 and OECM-1 cells treated with M1 and M2 conditioned media for 48 hr to induce epithelia and mesenchymal phenotype. **c-d** Western blots of STAT6 and GATA3 in SAS and A549 cells infected with a STAT6 expression vector or a control vector. **e-f** Western blots of STAT6 and GATA3 in OECM1 and A549 cells infected with a shRNA against STAT6 or a control sequence (pLKO). **g** Schema for representing the procedure of the animal experiment. The GFP-labelled 4T1 cells were orthotopically implanted to BALB/C mice. The primary and metastatic tumors were harvested 5 weeks after tumor implantation. n=6 for each group. **h** Western blots of Gata3 in primary-metastatic paired sample from 3 mice. **i** Quantification of expression of Gata3 protein. n=3. **j** Schema for representing the procedure of the animal experiment. The LLC cells were inoculated subcutaneously to the C57BL/6 mice and they were sacrificed at the end of 5th week. **k** Representative IHC of Gata3 in a pair of matched primary-metastatic tumors. Scale bar, 100 μm. **l** Schema for representing orthotopic SAS cells xenograft experiment. **m** Representative IHC of GATA3 in a matched primary-metastatic tumors 4 weeks after tumor inoculation. Scale bar, 100 μm. **n** Representative images for indicating the grading of intensity of GATA3 IHC results in human cancer samples. Scale bar, 50 μm. For the panels in supplementary Fig. 5, Data represent mean ± S.E.M. $^{*}p < 0.05$, $^{**}p < 0.01$, $^{***}p < 0.001$. Statistical analysis: Student's *t*-test (i).

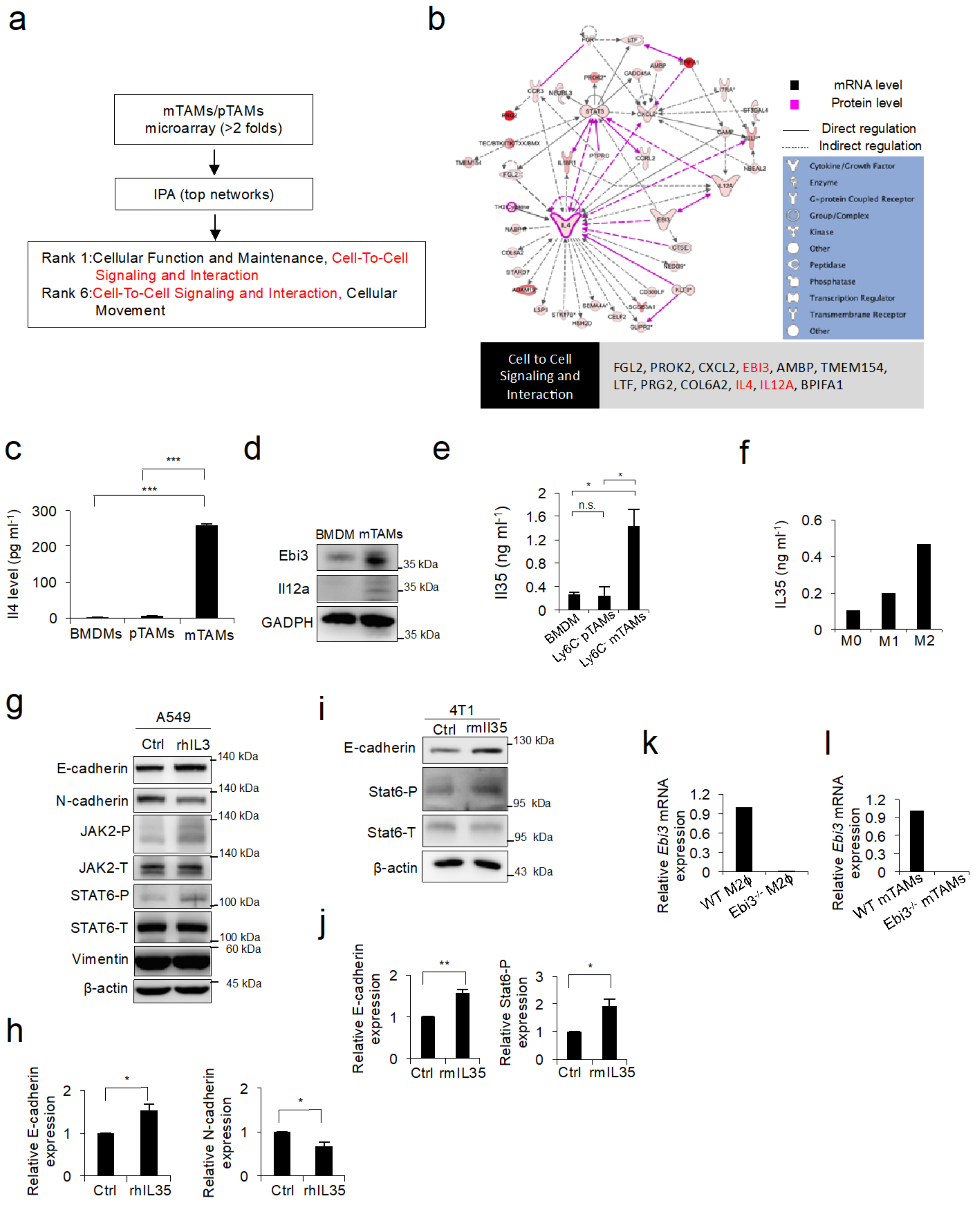

a
mTAMs/pTAMs microarray (>2 folds)
IPA (top networks)
Rank 1:Cellular Function and Maintenance, Cell-To-Cell Signaling and Interaction
Rank 6:Cell-To-Cell Signaling and Interaction, Cellular Movement
b
mRNA level
Protein level
Direct regulation
Indirect regulation
Cytokine/Growth Factor
Enzyme
G-protein Coupled Receptor
Group/Complex
Kinase
Other
Peptidase
Phosphatase
Transcription Regulator
Transmembrane Receptor
Other
Cell to Cell Signaling and Interaction
FGL2, PROK2, CXCL2, EBI3, AMBP, TMEM154, LTF, PRG2, COL6A2, IL4, IL12A, BPIFA1
c
Il4 level (pg ml-1)
300
200
100
0
BMDMs
pTAMs
mTAMs
***
***
d
BMDM mTAMs
Ebi3
Il12a
GADPH
35 kDa
35 kDa
35 kDa
e
Il35 (ng ml-1)
2
1.6
1.2
0.8
0.4
0
BMDM
Ly6C- pTAMs
Ly6C- mTAMs
n.s.
*
*
f
IL35 (ng ml-1)
0.6
0.4
0.2
0
M0
M1
M2
g
A549
Ctrl rhIL3
E-cadherin
N-cadherin
JAK2-P
JAK2-T
STAT6-P
STAT6-T
Vimentin
β-actin
140 kDa
140 kDa
140 kDa
140 kDa
100 kDa
100 kDa
60 kDa
45 kDa
h
Relative E-cadherin expression
Relative N-cadherin expression
2
1
0
Ctrl rhIL35
*
i
4T1
Ctrl rmIl35
E-cadherin
Stat6-P
Stat6-T
β-actin
130 kDa
95 kDa
95 kDa
43 kDa
j
Relative E-cadherin expression
Relative Stat6-P expression
Ctrl rmIL35
**
*
k
Relative Ebi3 mRNA expression
1.2
0.9
0.6
0.3
0
WT M2ϕ
Ebi3-/- M2ϕ
l
Relative Ebi3 mRNA expression
WT mTAMs
Ebi3-/- mTAMs

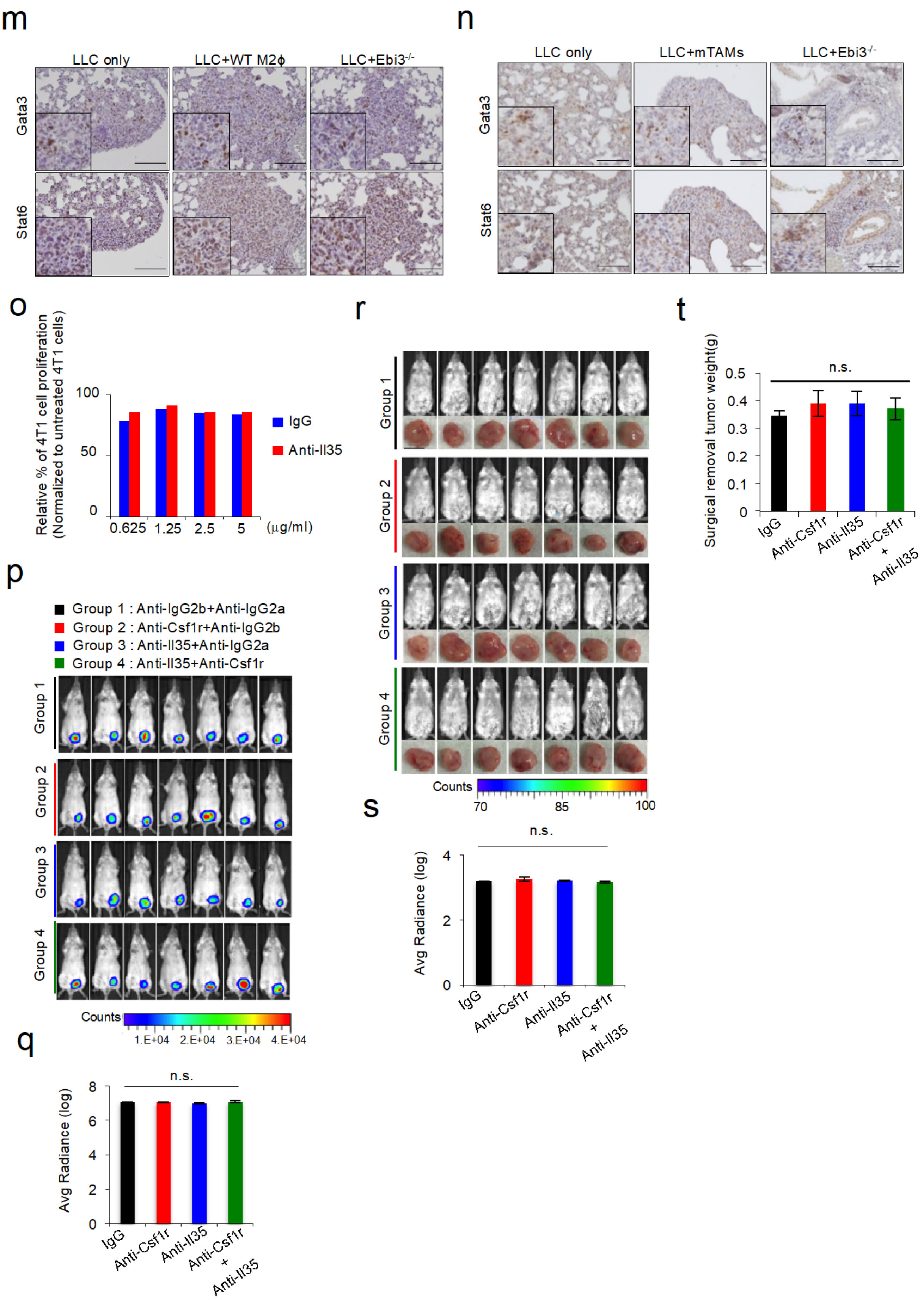
m
LLC only
LLC+WT M2ϕ
LLC+Ebi3-/-
Gata3
Stat6
n
LLC only
LLC+mTAMs
LLC+Ebi3-/-
Gata3
Stat6
o
Relative % of 4T1 cell proliferation (Normalized to untreated 4T1 cells)
100
50
0
0.625
1.25
2.5
5
(μg/ml)
IgG
Anti-Il35
r
Group 1
Group 2
Group 3
Group 4
Counts
70
85
100
t
Surgical removal tumor weight(g)
n.s.
0.5
0.4
0.3
0.2
0.1
0
IgG
Anti-Csf1r
Anti-Il35
Anti-Csf1r + Anti-Il35
p
Group 1 : Anti-IgG2b+Anti-IgG2a
Group 2 : Anti-Csf1r+Anti-IgG2b
Group 3 : Anti-Il35+Anti-IgG2a
Group 4 : Anti-Il35+Anti-Csf1r
Group 1
Group 2
Group 3
Group 4
Counts
1.E+04
2.E+04
3.E+04
4.E+04
s
n.s.
Avg Radiance (log)
4
2
0
IgG
Anti-Csf1r
Anti-Il35
Anti-Csf1r + Anti-Il35
q
n.s.
Avg Radiance (log)
8
6
4
2
0
IgG
Anti-Csf1r
Anti-Il35
Anti-Csf1r + Anti-Il35

Supplementary Fig 6. TAM-secreted IL-35 in metastatic colonization. **a** Flowchart for mining the pathways involved in mTAMs-mediated metastasis. **b** IPA of the upregulated genes in mTAMs. **C** ELISA of secreted Il-4 24 hr after cultivation. n=3 independent experiments (each contains 2 technical replicates). **d** Western blots of Ebi3 and Il12α in Ly6C^{-} mTAMs and BMDM. **E** ELISA of secreted Il-35 from the indicated TAMs/BMDM 24 hr after cultivation. n=3 independent experiments (each contains 2 technical replicates). **f** ELISA of secreted IL-35 24 hr after cultivation. n=2 independent experiments (each contains 2 technical replicates). **g** Representative western blots of total JAK2, phosphorylated JAK2, phosphorylated STAT6, and EMT markers (E-cadherin, N-cadherin, Vimentin) in A549 upon recombinant human IL-35 (rhIL-35) treatment (50 ng ml^{-1}) for 48 hr. **h** Quantification of E-cadherin and N-cadherin proteins. n=3. **i** Representative western blots of total, phosphorylated STAT6, and E-cadherin in 4T1 upon recombinant murine IL-35 (rmIL-35) treatment (50 ng ml^{-1}) for 48 hr. **j** Quantification of E-cadherin and phosphorylated STAT6 proteins. n=3. **k-l** RT-qPCR of *Ebi3* in WT/*Ebi3*$^{-/-}$ M2-like macrophages and WT/*Ebi3*$^{-/-}$ mTAMs. **m** IHC of Gata3 and Stat6 in metastatic tumor from the group co-injected with tumor only, WT M2-like macrophages, or *Ebi3*$^{-/-}$ M2-like macrophages. Magnification in the inset. Scale bar, 100 μm. **n** IHC of Gata3 and Stat6 in metastatic tumor from the group of co-injection with tumor only, WT mTAMs, or *Ebi3*$^{-/-}$ mTAMs. Magnification in the inset. Scale bar, 100 μm. **o** MTT assay for 4T1 treated with an anti-IL-35 antibody or IgG. n=2 independent experiments (each contains 4 technical replicates). **p** Bioluminescent signals for primary tumor from 4T1-BALB/c orthotopic model 3 weeks after tumor inoculation. **q** Quantification of bioluminescent imaging. **r** Photos for the surgically-removed tumors and the elimination of the bioluminescent signals in mice after surgery. Scale bar, 1 cm. **s** Quantification of bioluminescent imaging. **t** Quantification of the surgically-removed tumor weight of 4T1-BALB/c orthotopic model. n=7. For the panels in supplementary Fig. 6, Data represent mean ± S.E.M. $^{*}p < 0.05$, $^{**}p < 0.01$, $^{***}p < 0.001$. n.s., non-significance. Statistical analysis: Student's *t*-test (c, e, h, j, q, s, t).

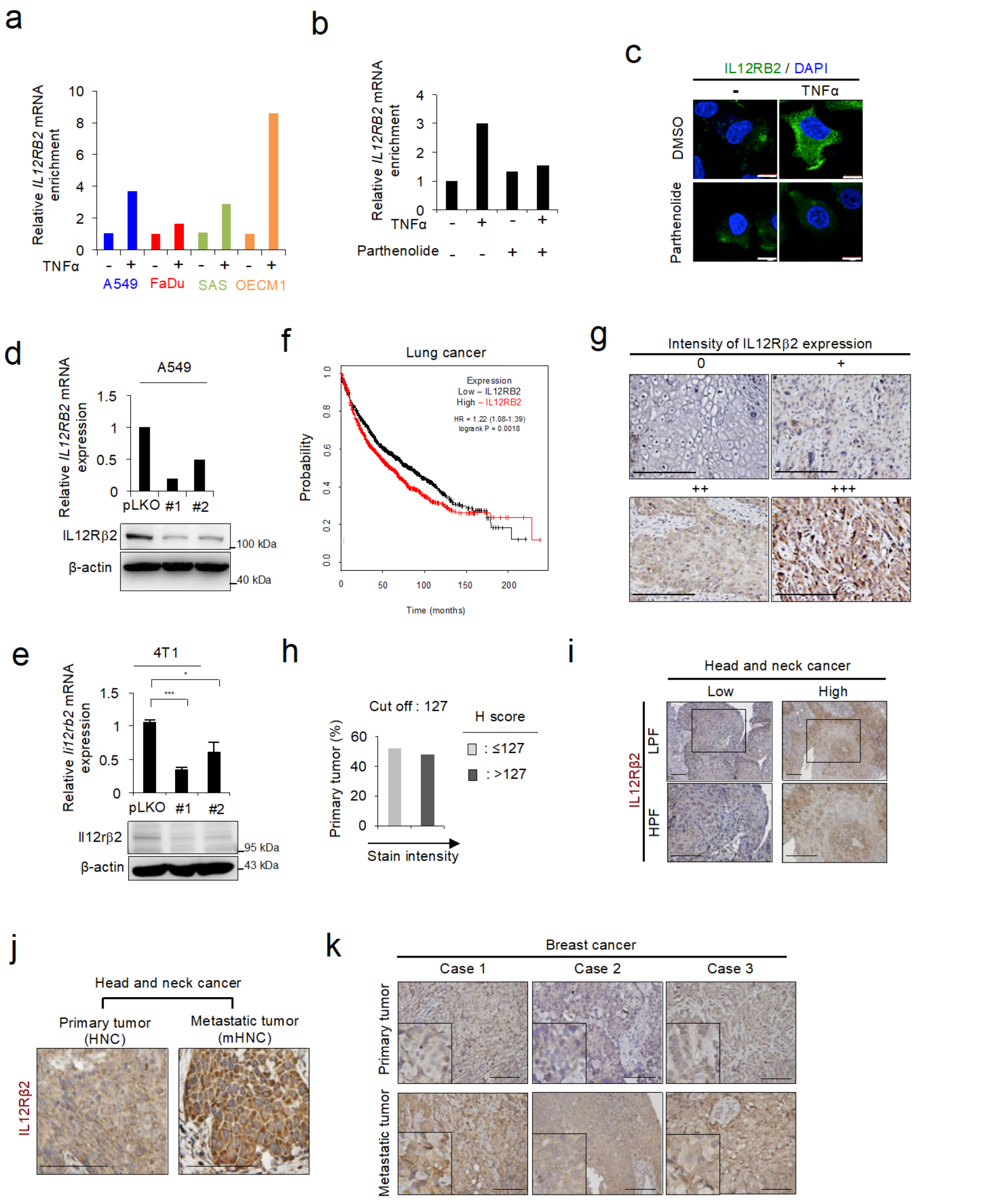
a
Relative IL12RB2 mRNA enrichment
TNFα
A549
FaDu
SAS
OECM1
b
Relative IL12RB2 mRNA enrichment
TNFα
Parthenolide
c
IL12RB2 / DAPI
TNFα
DMSO
Parthenolide
d
A549
Relative IL12RB2 mRNA expression
pLKO
#1
#2
IL12Rβ2
β-actin
100 kDa
40 kDa
f
Lung cancer
Expression
Low – IL12RB2
High – IL12RB2
HR = 1.22 (1.08-1.39)
logrank P = 0.0018
Probability
Time (months)
g
Intensity of IL12Rβ2 expression
0
+
++
+++
e
4T1
Relative Il12rb2 mRNA expression
pLKO
#1
#2
Il12rβ2
β-actin
95 kDa
43 kDa
h
Cut off : 127
H score
: ≤127
: >127
Primary tumor (%)
Stain intensity
i
Head and neck cancer
Low
High
LPF
HPF
IL12Rβ2
j
Head and neck cancer
Primary tumor (HNC)
Metastatic tumor (mHNC)
IL12Rβ2
k
Breast cancer
Case 1
Case 2
Case 3
Primary tumor
Metastatic tumor

Supplementary Fig 7. Expression of IL12Rβ2 correlates with cancer metastasis. **a** RT-qPCR for *IL12RB2* in different cancer cell lines upon TNFα (20 ng ml^{-1}) treatment for 24 hr. n=2 independent experiments (each contains 2 technical replicates). **b** RT-qPCR for *IL12RB2* in A549 treated as indicated reagents for 24 hr. Working concentration for TNFα: 20 ng/ml; parthenolide: 10 μm. n=2 independent experiments (each contains 2 technical replicates). **c** Immunofluorescent staining of IL12Rβ2 in A549 cells upon with/without TNFα (20 ng ml^{-1}) and parthenolide (10 μm) treatment for 24 hr. Scale bar, 10 μm. **d** RT-qPCR and western blots for confirming the knockdown efficiency of the IL12Rβ2 in A549 receiving the shRNA against *IL12RB2* or a control sequence (pLKO). The number indicates two independent sequences for shRNA experiments. n=2 independent experiments (each contains 2 technical replicates). **e** RT-qPCR and western blots for confirming the knockdown efficiency of the Il12rβ2 in 4T1 cells receiving the shRNA against *Il12rb2* or a control sequence (pLKO). The number indicates two independent sequences for shRNA experiments. n=3 independent experiments (each contains 2 technical replicates). **f** Kaplan-Meier survival analysis for showing the prognostic impact of *L12RB2* expression in lung cancer patient samples. The p-value was estimated by log-rank test. The data were obtained from Kaplan-Meier Plotter [http://kmplot.com/]. **g** Representative images for showing the intensity grading (0 ~ +++) of IL12Rβ2 in human head and neck cancer samples. Scale bar, 200 μm. **h** Histogram showing the percentages of primary tumors exhibiting low and high level of IL12Rβ2. **i** IHC result of IL12Rβ2 in 91 head and neck cancer samples. Scale bar, 100 μm. **j** Representative IHC of IL12Rβ2 in paired primary-metastatic tumor of head and neck cancer. Scale bar, 100 μm. HNC, head and neck cancers; mHNC, metastatic head and neck cancer. **k** Representative IHC of IL12Rβ2 in paired primary-metastatic tumors of breast cancer. Scale bar, 100 μm. For the panels in supplementary Fig. 7, Data represent mean ± S.E.M. $^{*}p < 0.05$, $^{**}p < 0.01$, $^{***}p < 0.001$. Statistical analysis: Student’s *t*-test (e) and log-rank test for Kaplan-Meier survival analysis (f).

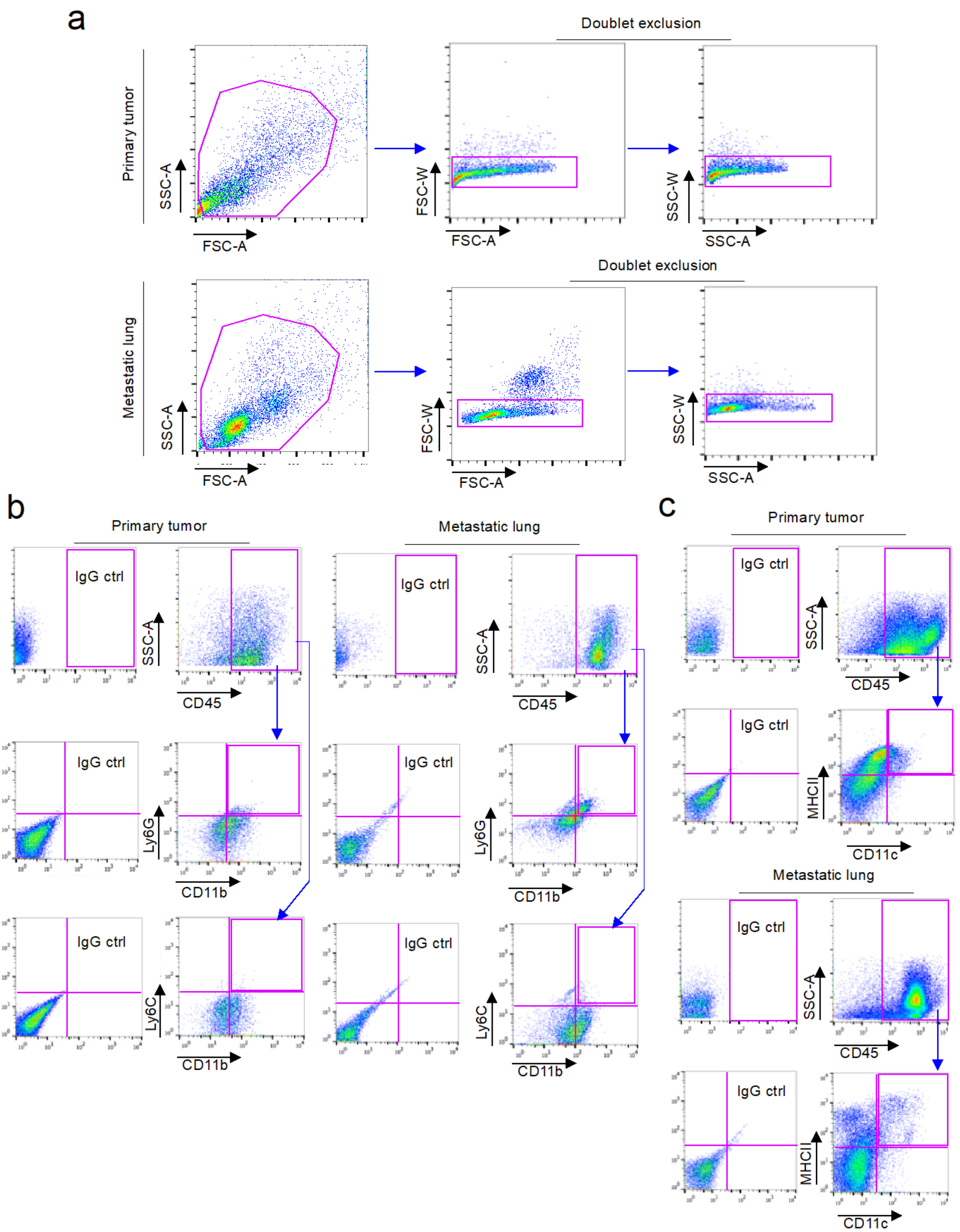
a
Doublet exclusion
Primary tumor
SSC-A
FSC-A
FSC-W
FSC-A
SSC-W
SSC-A
Doublet exclusion
Metastatic lung
SSC-A
FSC-A
FSC-W
FSC-A
SSC-W
SSC-A
b
Primary tumor
Metastatic lung
IgG ctrl
SSC-A
CD45
IgG ctrl
SSC-A
CD45
IgG ctrl
Ly6G
CD11b
IgG ctrl
Ly6G
CD11b
IgG ctrl
Ly6C
CD11b
IgG ctrl
Ly6C
CD11b
c
Primary tumor
IgG ctrl
SSC-A
CD45
IgG ctrl
MHCII
CD11c
Metastatic lung
IgG ctrl
SSC-A
CD45
IgG ctrl
MHCII
CD11c

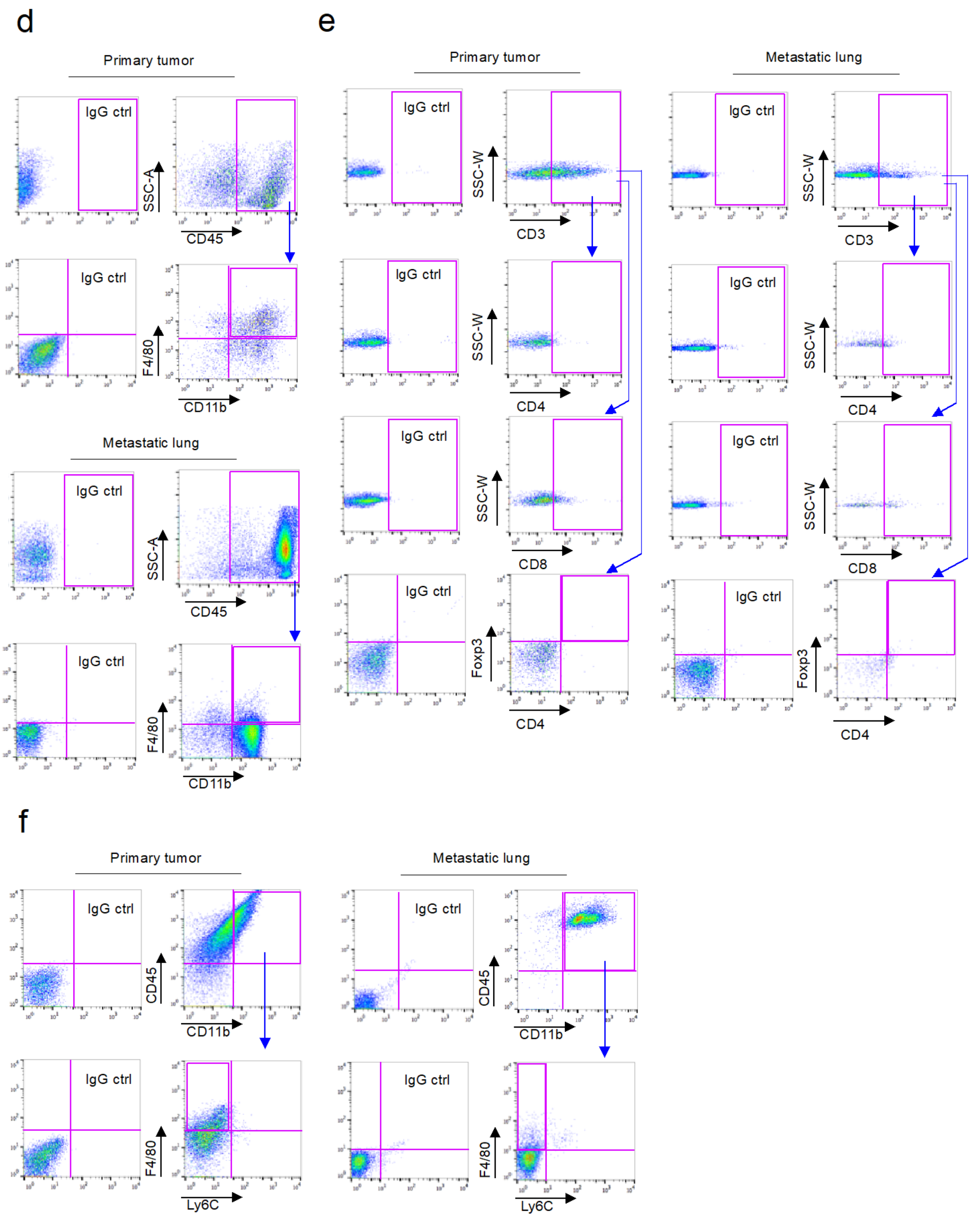
d
Primary tumor
IgG ctrl
SSC-A
CD45
IgG ctrl
F4/80
CD11b
Metastatic lung
IgG ctrl
SSC-A
CD45
IgG ctrl
F4/80
CD11b
e
Primary tumor
IgG ctrl
SSC-W
CD3
IgG ctrl
SSC-W
CD4
IgG ctrl
SSC-W
CD8
IgG ctrl
Foxp3
CD4
Metastatic lung
IgG ctrl
SSC-W
CD3
IgG ctrl
SSC-W
CD4
IgG ctrl
SSC-W
CD8
IgG ctrl
Foxp3
CD4
f
Primary tumor
IgG ctrl
CD45
CD11b
IgG ctrl
F4/80
Ly6C
Metastatic lung
IgG ctrl
CD45
CD11b
IgG ctrl
F4/80
Ly6C

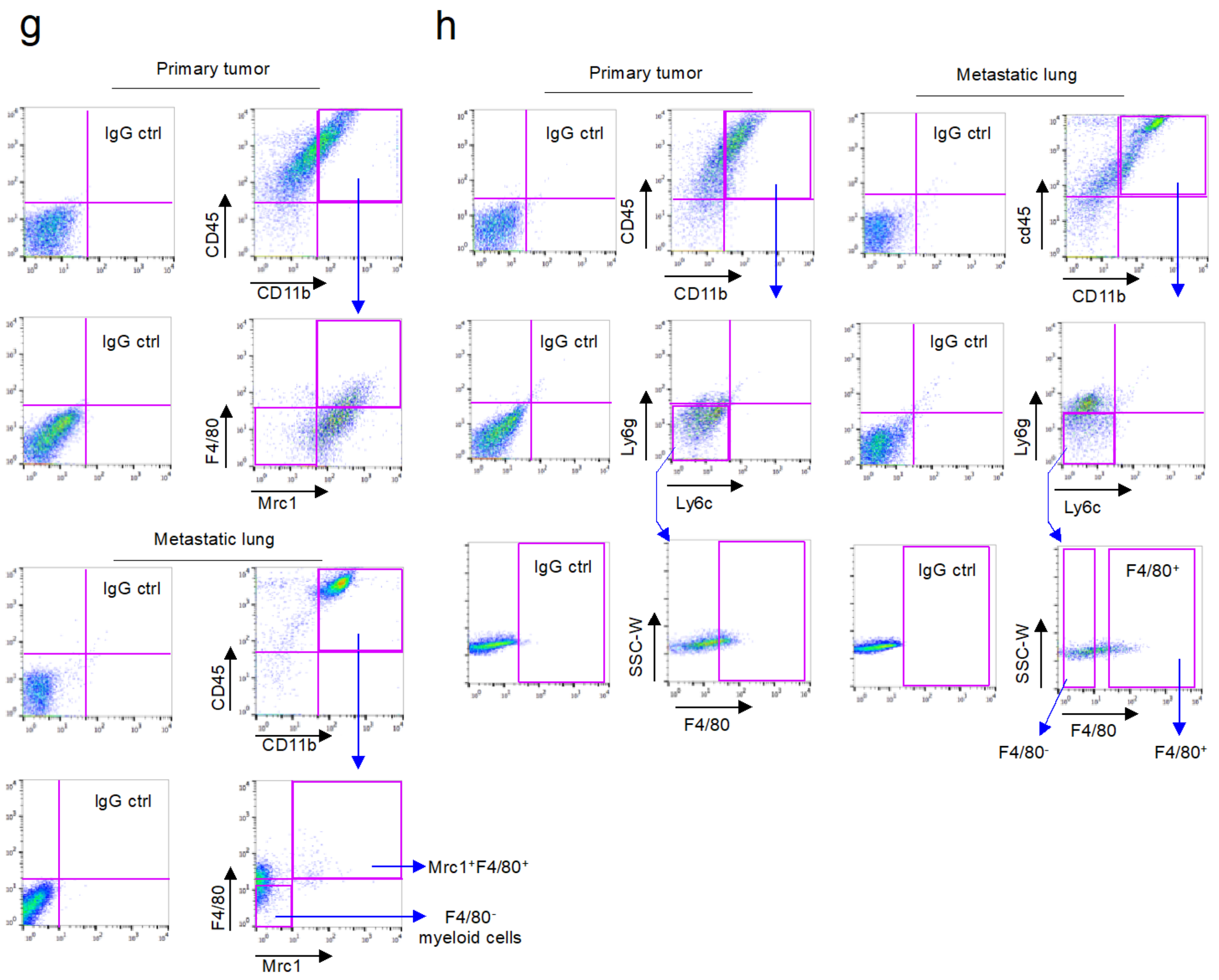

Supplementary Fig 8. Gating strategies of flow cytometry. **a** Representative data of the first-step gating scheme for all immune cells sorted from primary tumors and metastatic lungs of the 4T1 orthotopic tumor model. **b** Representative gating scheme of CD11b^{+}Ly6G^{+} granulocytic MDSCs (G-MDSCs) and CD11b^{+}Ly6C^{+} monocytic MDSCs (M-MDSCs) isolated from primary tumors and metastatic lungs used in supplementary Fig. 1b. **c** Representative gating scheme of CD11c^{+}MHCII^{+} DCs isolated from primary tumors and metastatic lungs used in supplementary Fig. 1b. **d** Representative gating scheme of CD11b^{+}F4/80^{+} macrophages isolated from primary tumors and metastatic lungs used in Fig. 1a, Fig. 1c, Fig. 1d, Fig. 3b, supplementary Fig. 1b, supplementary Fig. 3b, supplementary Fig. 3c, and supplementary Fig. 6a. **e** Representative gating scheme of innate immune cells (CD4^{+} T cells CD8^{+} T cells, and CD4^{+}Foxp3^{+} Treg) isolated from primary tumors and metastatic lungs used in supplementary Fig. 1c. **f** Representative gating scheme of CD11b^{+}F4/80^{+}Ly6C^{-} macrophages isolated from primary tumors and metastatic lungs used in Fig. 2h, Fig. 3b, Fig. 6a, supplementary Fig. 6c, supplementary Fig. 6d, and supplementary Fig. 6e. **g** Representative gating scheme of CD11b^{+}F4/80^{+}Mrc1^{+} macrophages isolated from primary tumors and metastatic lungs used in Fig. 2l and supplementary Fig. 1f. **h** Representative gating scheme of CD11b^{+}F4/80^{+}Ly6G^{-}Ly6C^{-} macrophages isolated from primary tumors and metastatic lungs used in Fig. 4w, Fig. 6b, Fig. 6j, and Fig. 7n.

Supplementary Fig. 9 Uncropped films of the experiments displayed in the figures and Supplementary figures

Fig. 3b

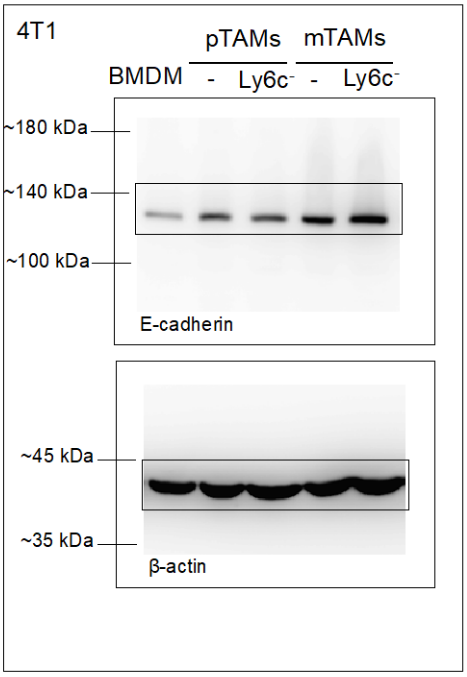

Fig. 4b

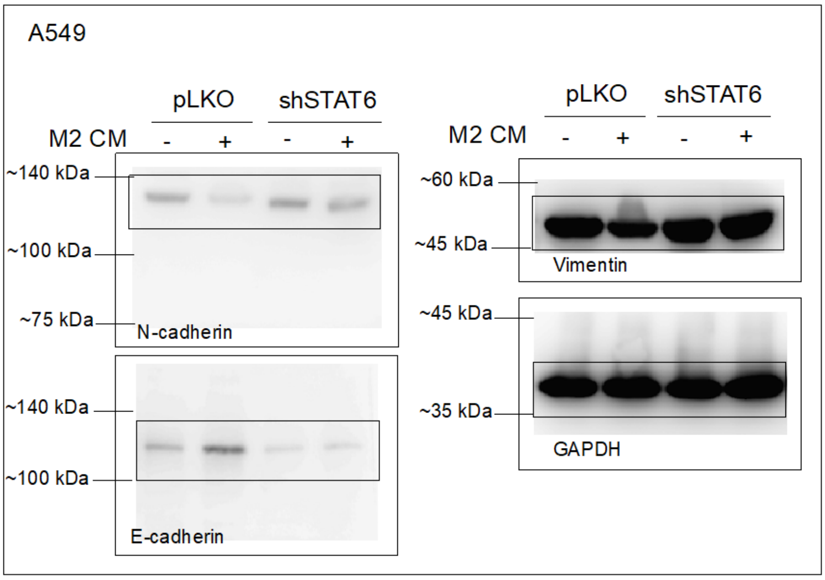

Fig. 4d

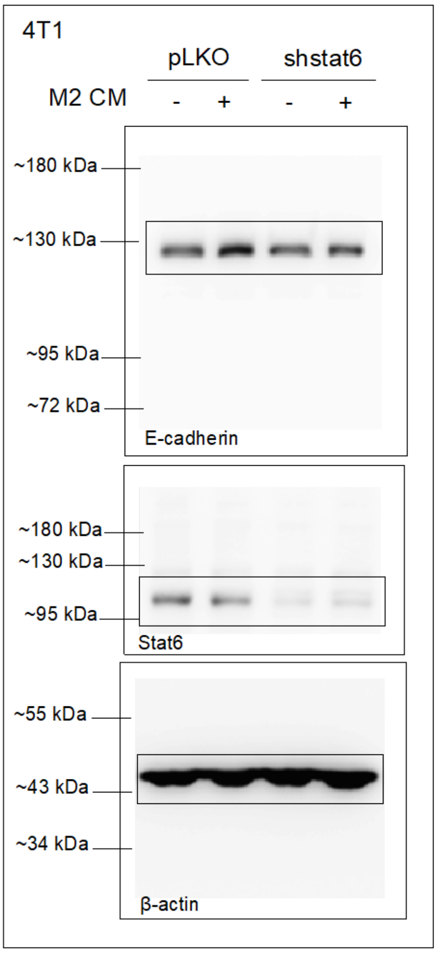

Fig. 3e and Fig. 3g

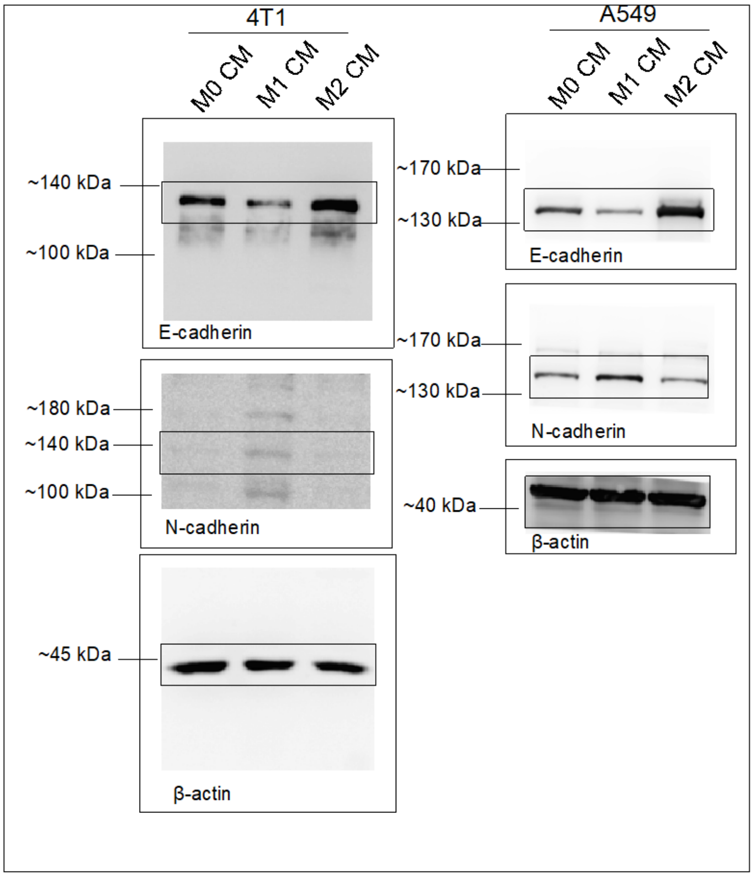

Fig. 4f

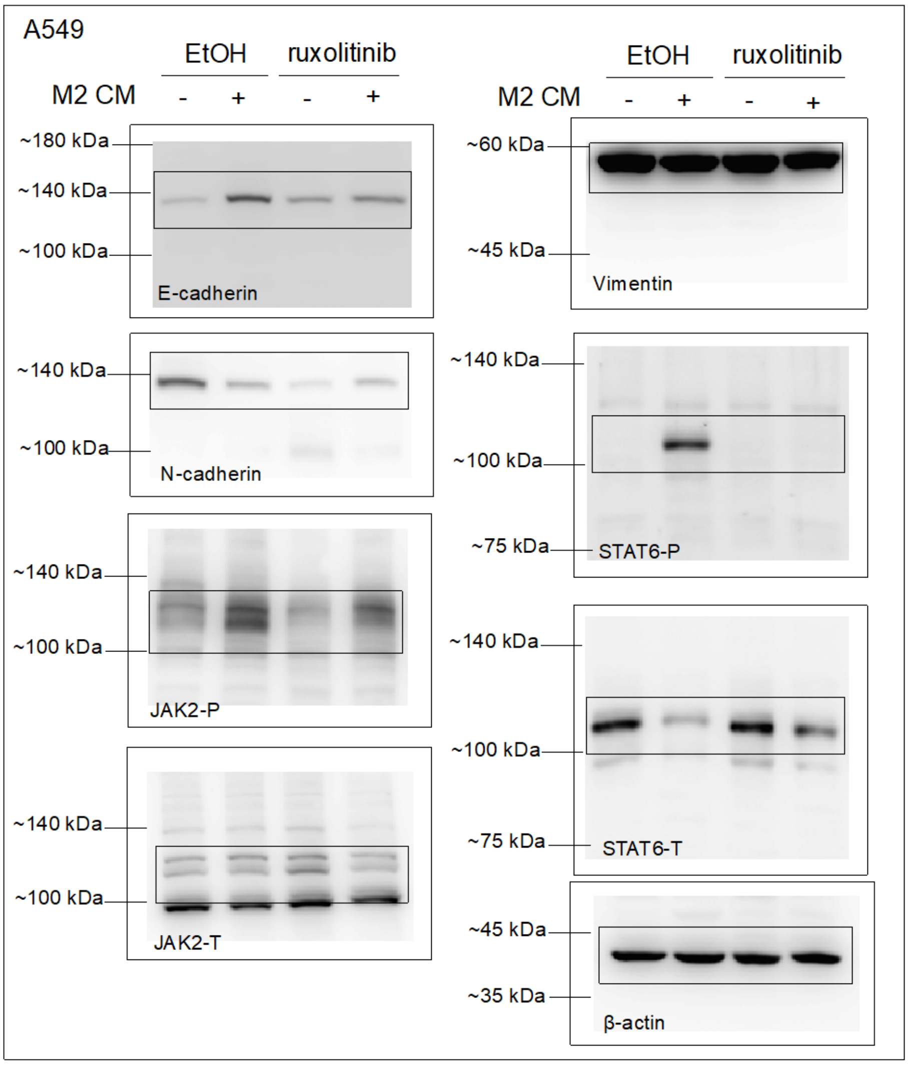

Fig. 5a

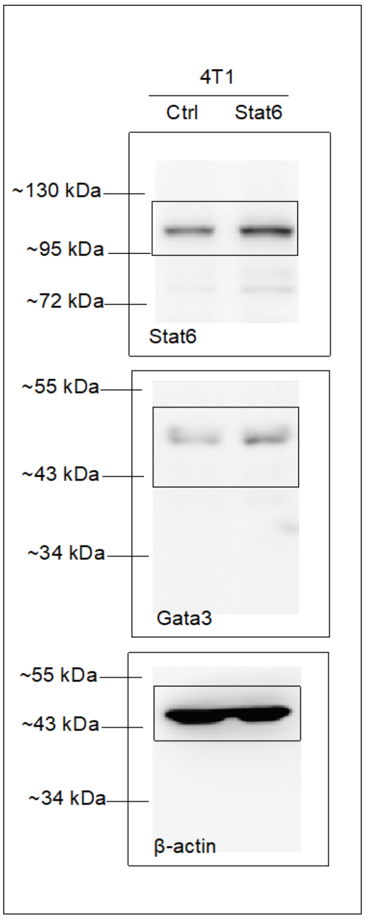

Fig. 4h

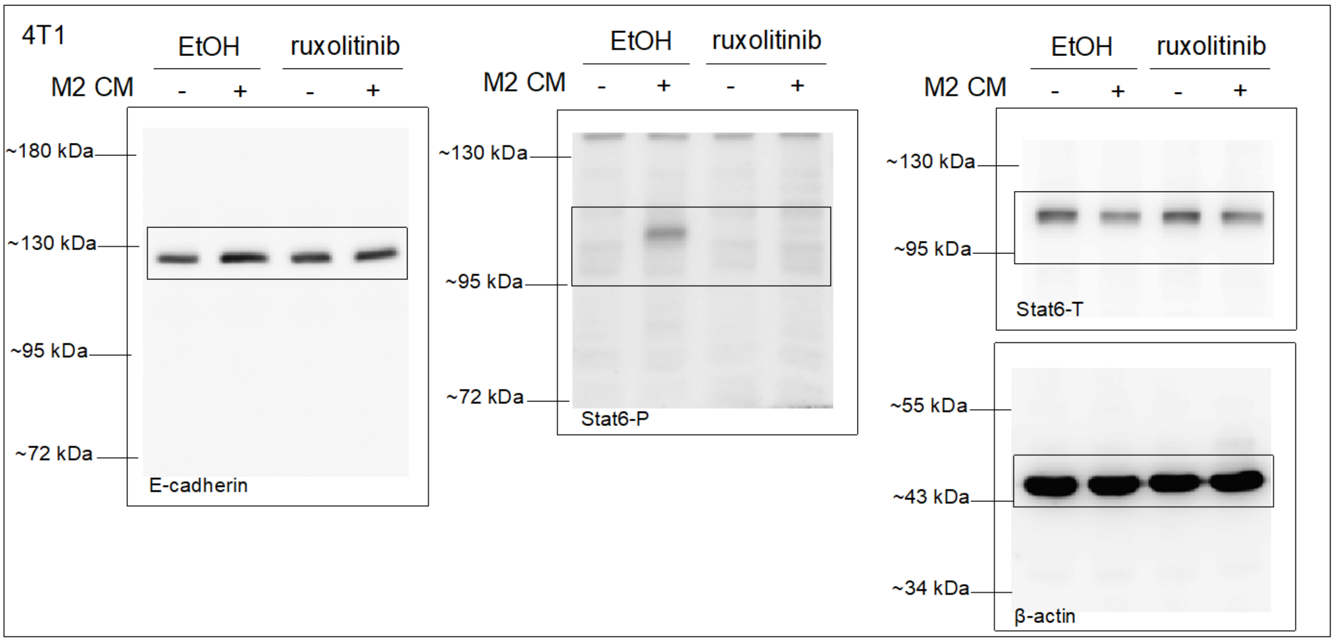

Fig. 5c

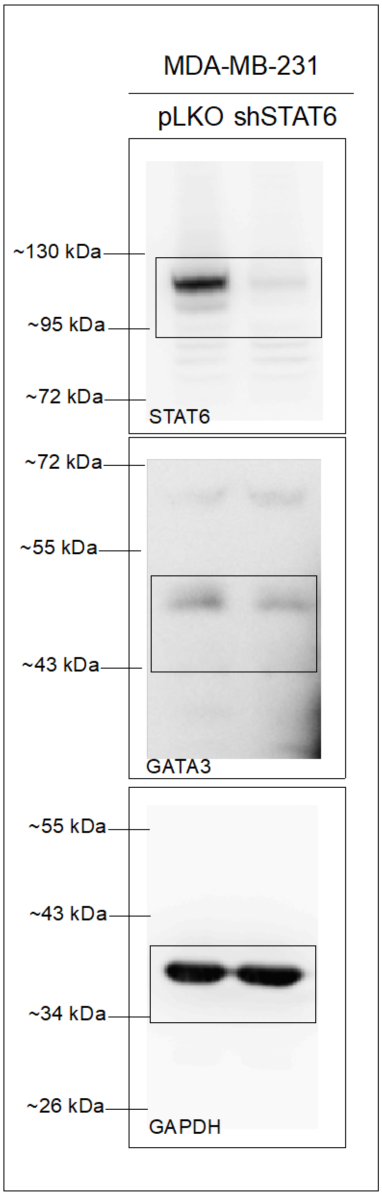

Fig. 5e and Fig. 5g

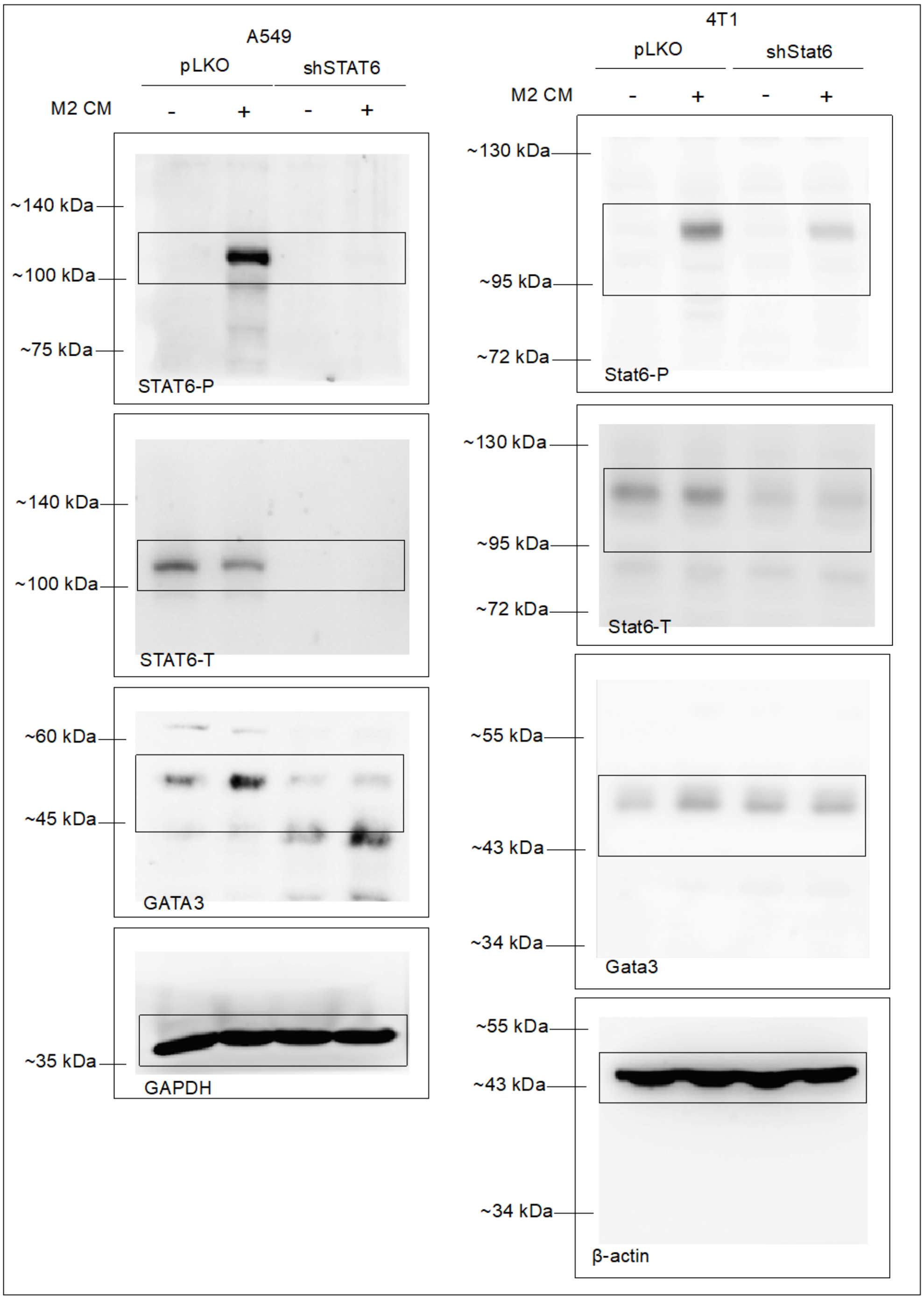

Fig. 7a

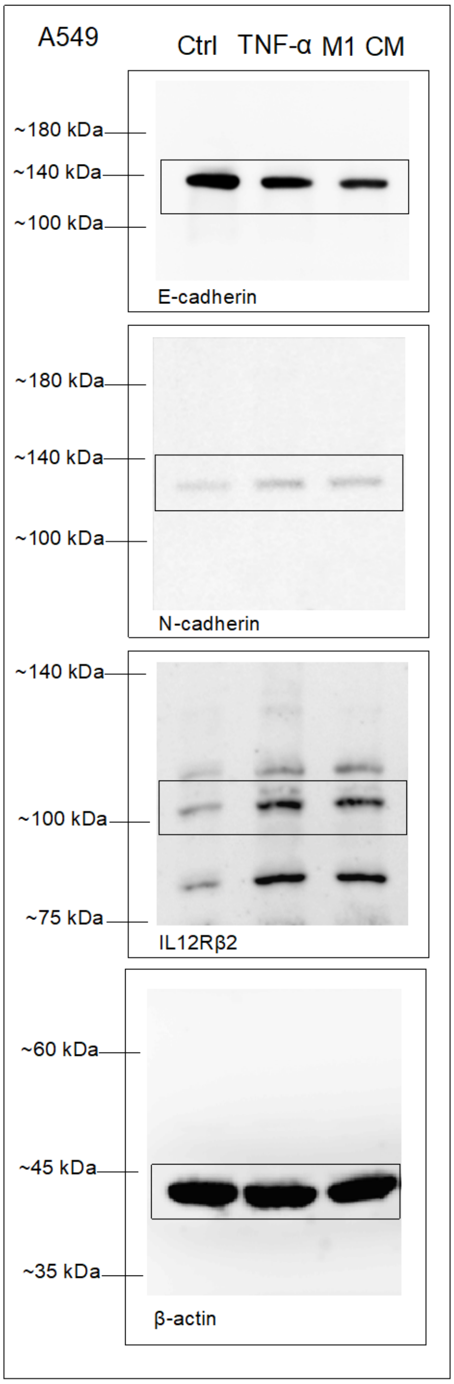

Fig. 7b

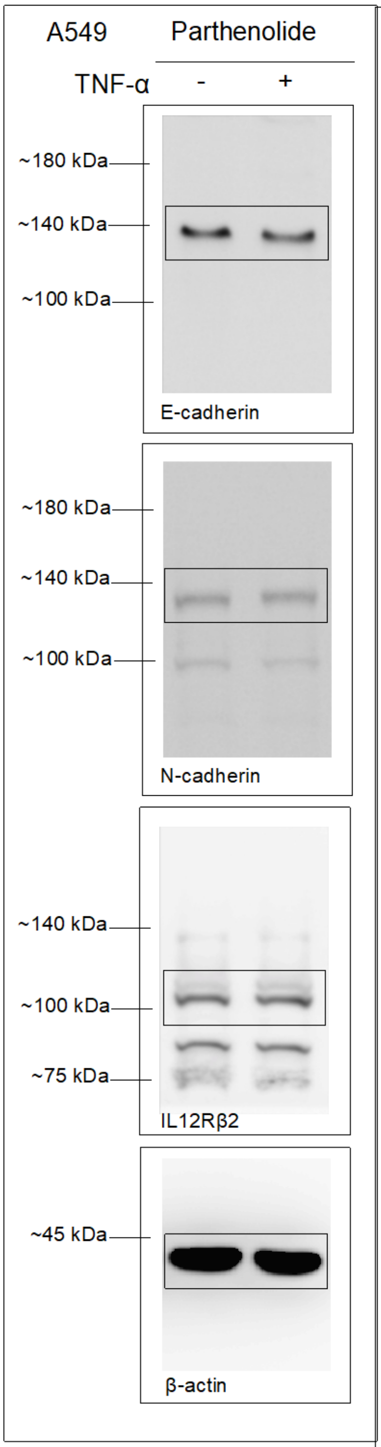

Fig. 7e

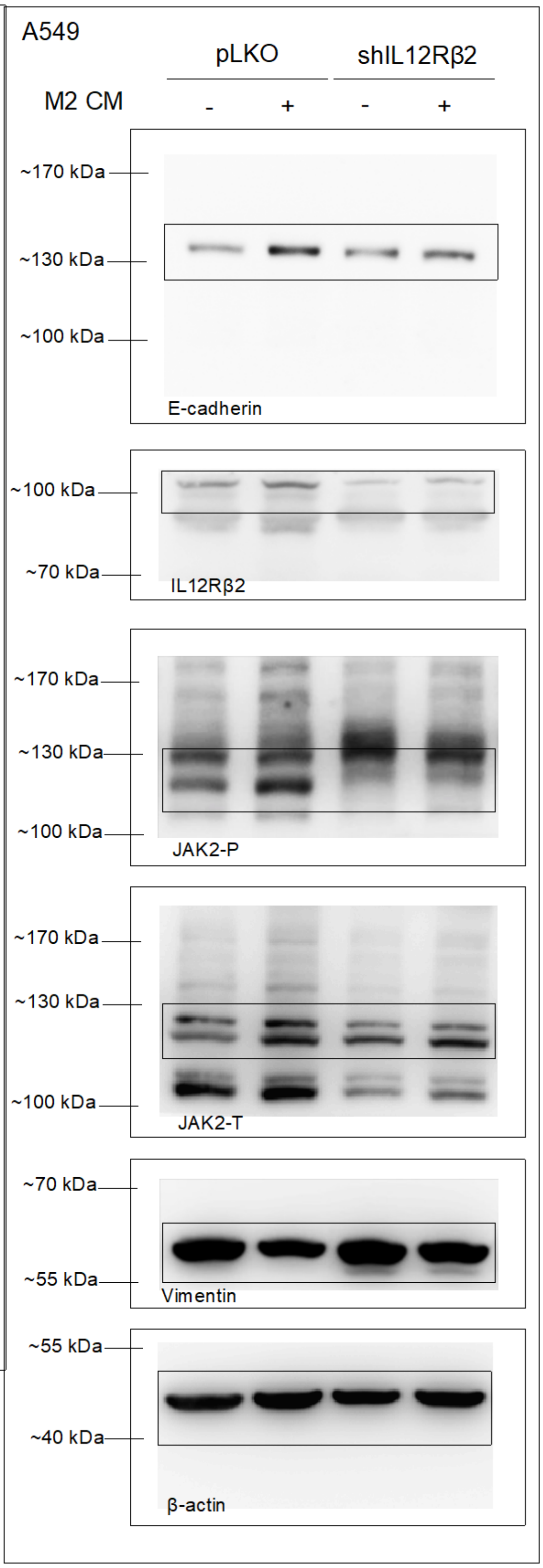

Fig. 7g

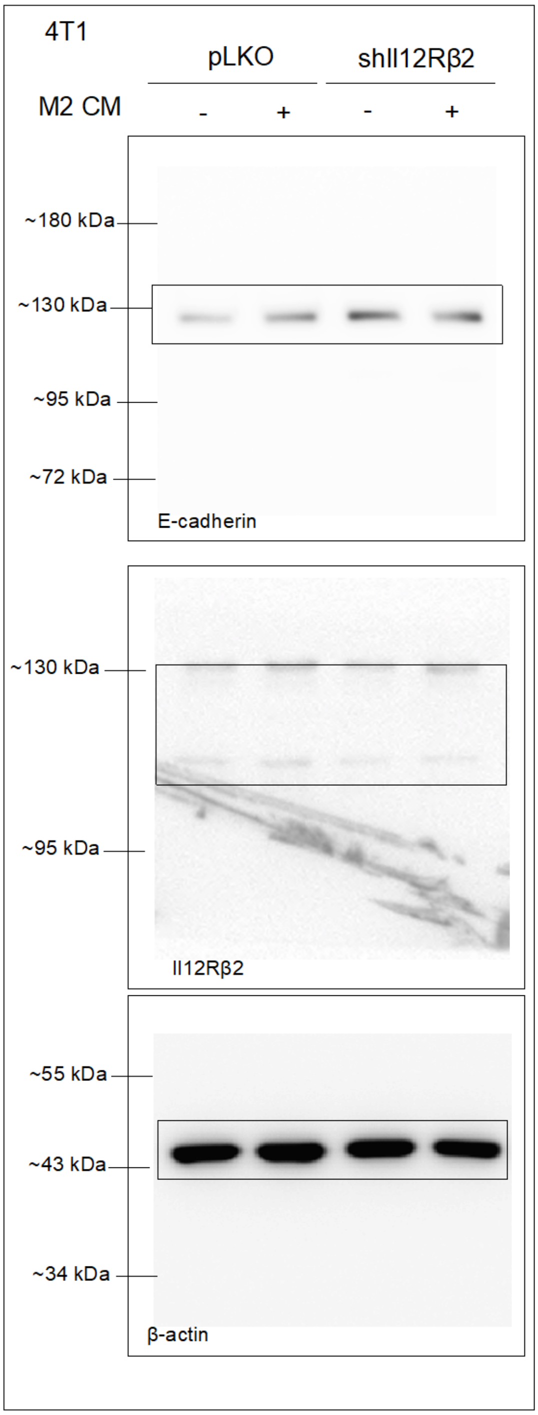
4T1
pLKO
shIl12Rβ2
M2 CM
-
+
-
+
~180 kDa
~130 kDa
~95 kDa
~72 kDa
E-cadherin
~130 kDa
~95 kDa
Il12Rβ2
~55 kDa
~43 kDa
~34 kDa
β-actin

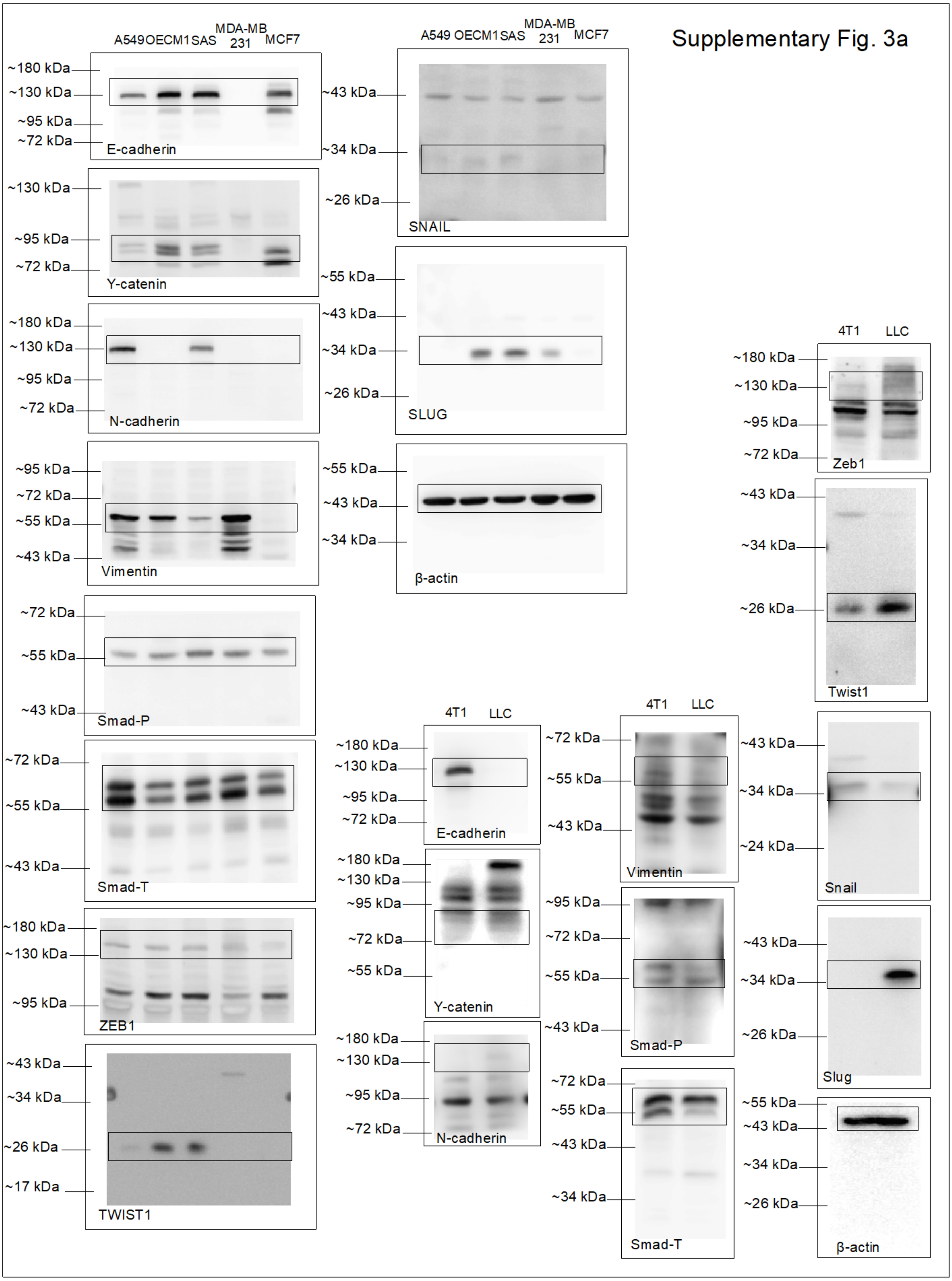

Supplementary Fig. 3a
A549 OECM1 SAS MDA-MB 231 MCF7
E-cadherin
Y-catenin
N-cadherin
Vimentin
Smad-P
Smad-T
ZEB1
TWIST1
SNAIL
SLUG
β-actin
4T1 LLC
Zeb1
Twist1
E-cadherin
Y-catenin
N-cadherin
Vimentin
Smad-P
Smad-T
Snail
Slug
β-actin
~180 kDa
~130 kDa
~95 kDa
~72 kDa
~55 kDa
~43 kDa
~34 kDa
~26 kDa
~24 kDa
~17 kDa

Supplementary Fig. 4d

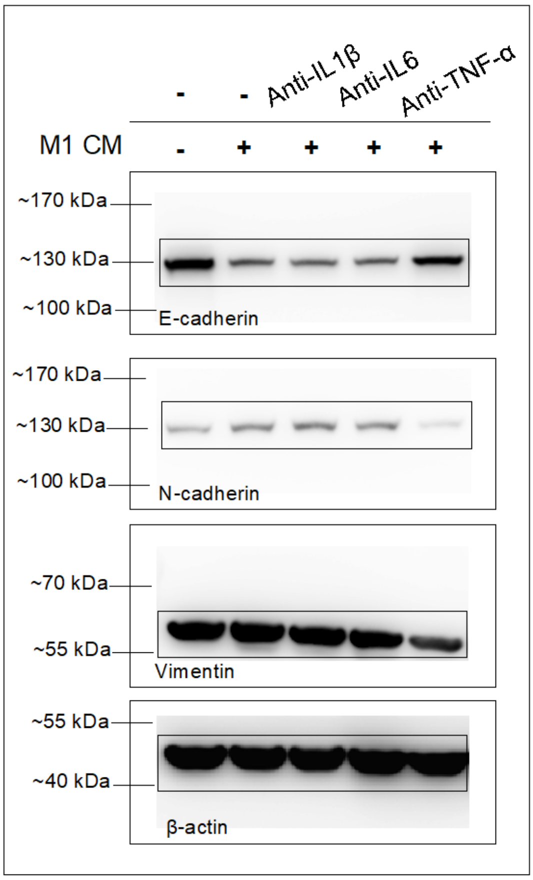

Supplementary Fig. 4g

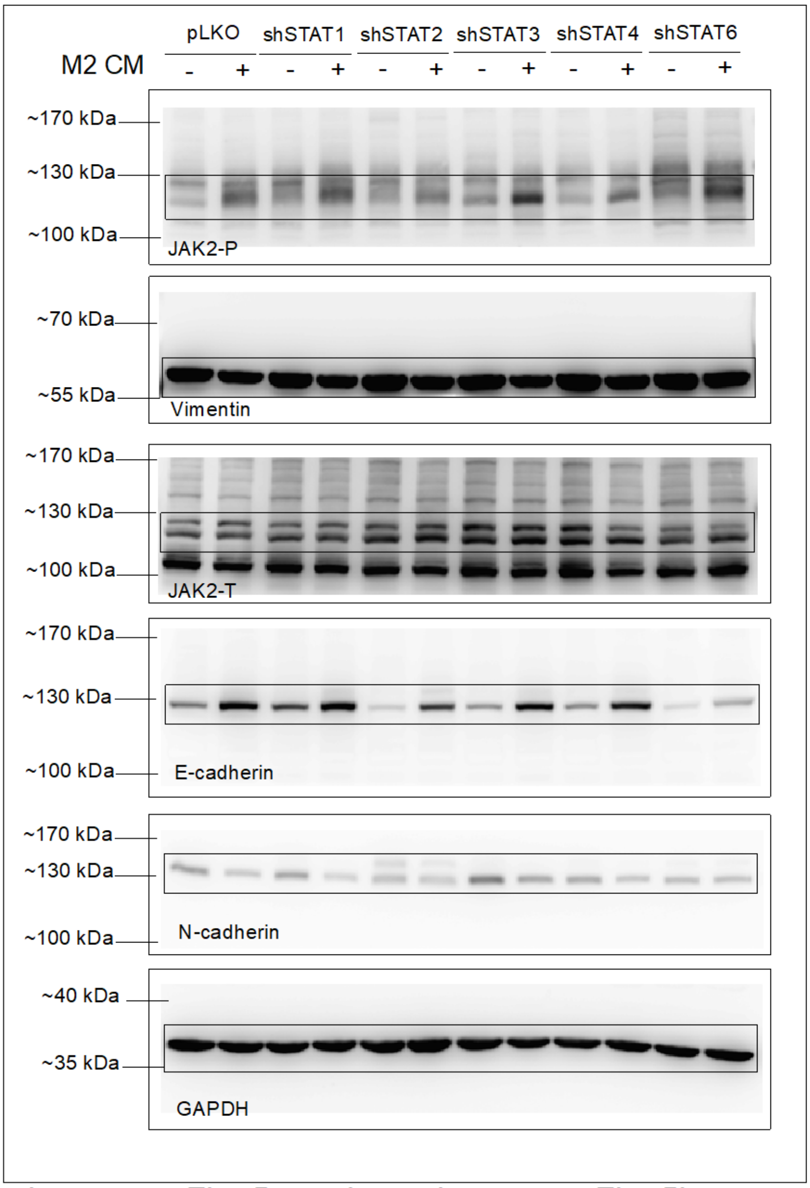

Supplementary Fig. 4j

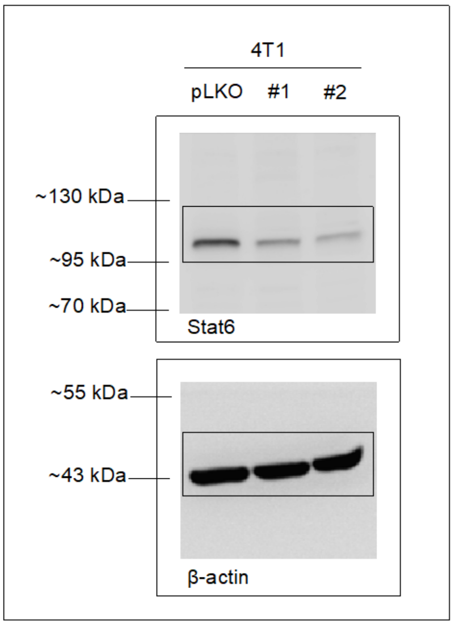

Supplementary Fig. 5a and supplementary Fig. 5b

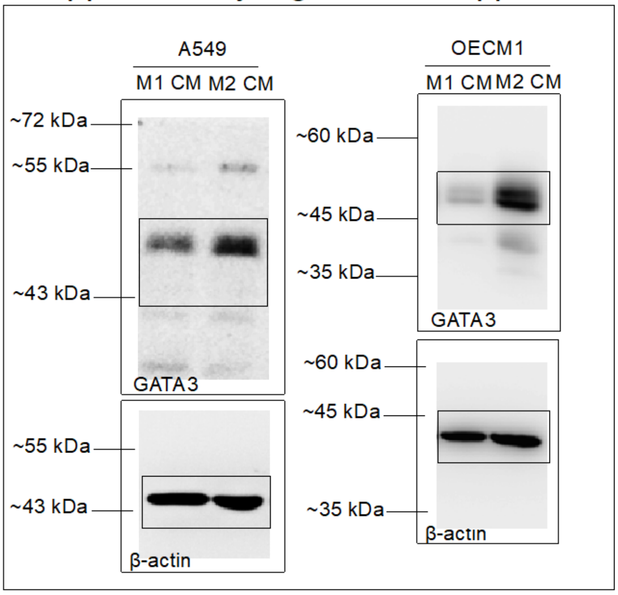

Supplementary Fig. 5c and supplementary Fig. 5d

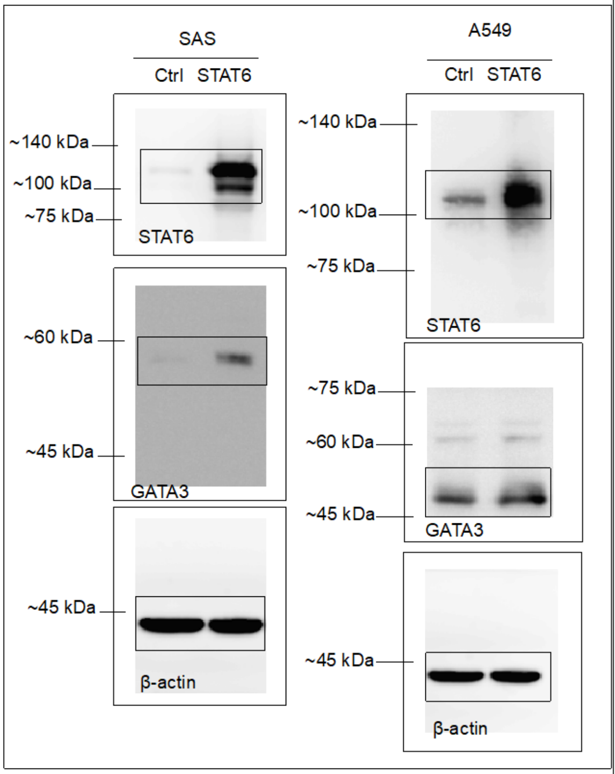

Supplementary Fig. 5e and supplementary Fig. 5f

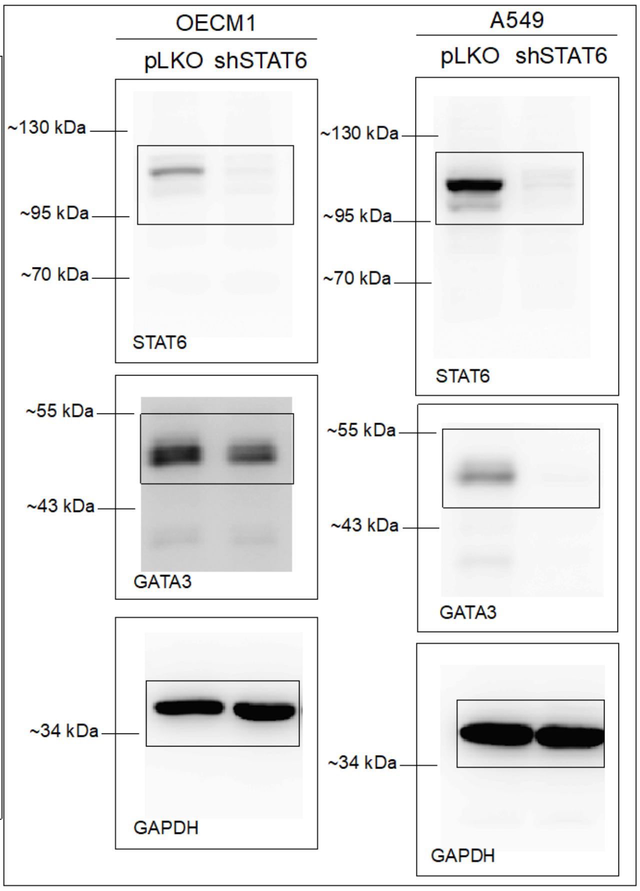

Supplementary Fig. 6d

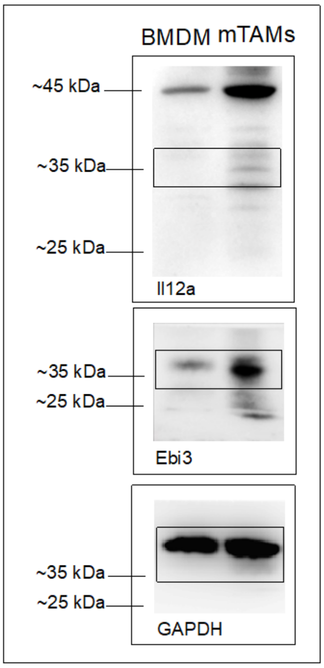

Supplementary Fig. 5h

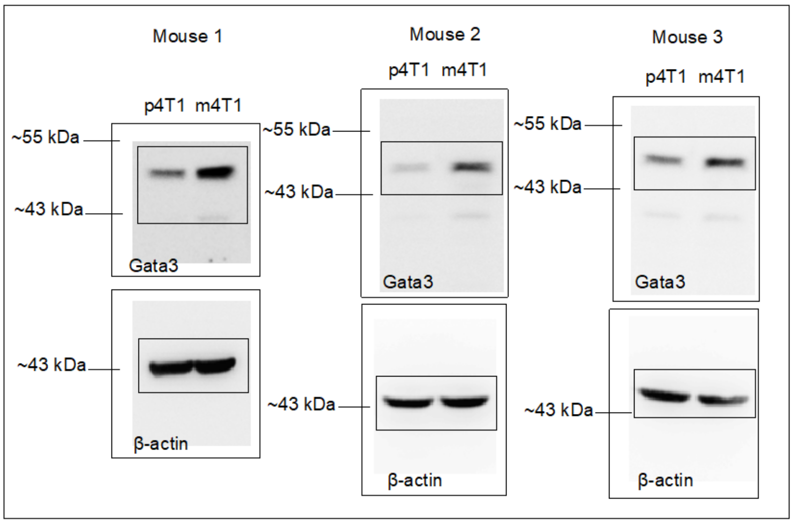

Supplementary Fig. 6g

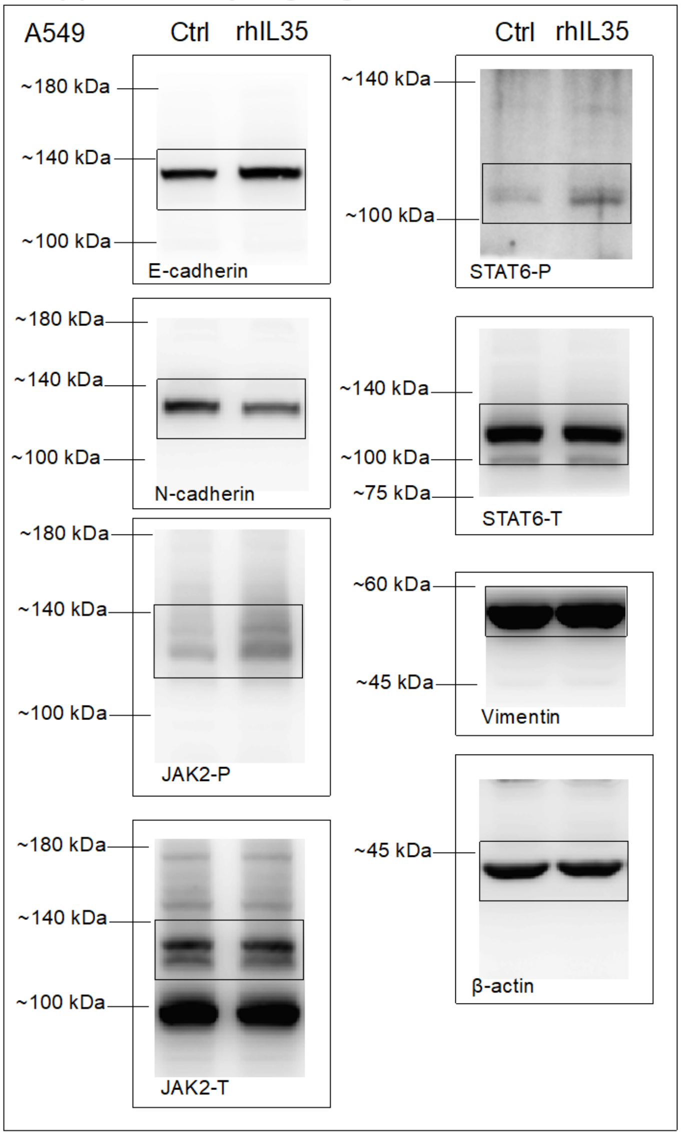

Supplementary Fig. 6i

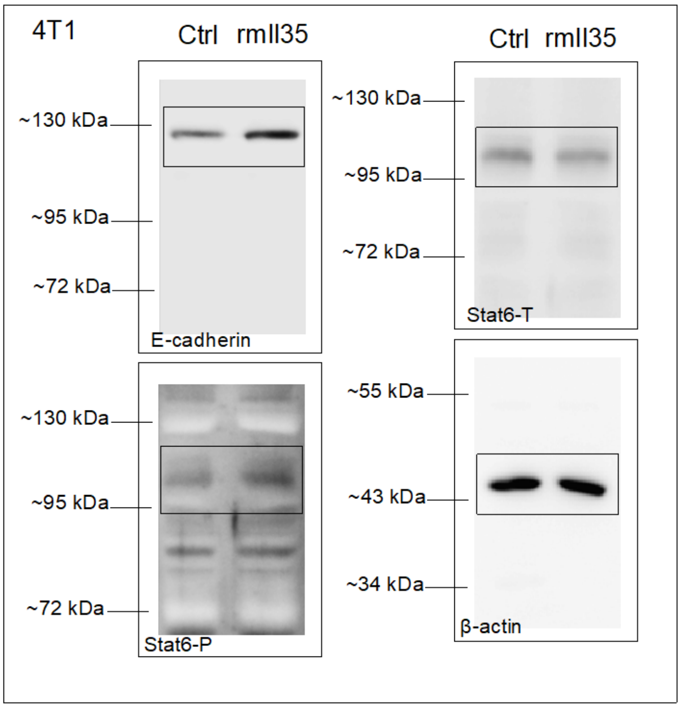

Supplementary Fig. 7d

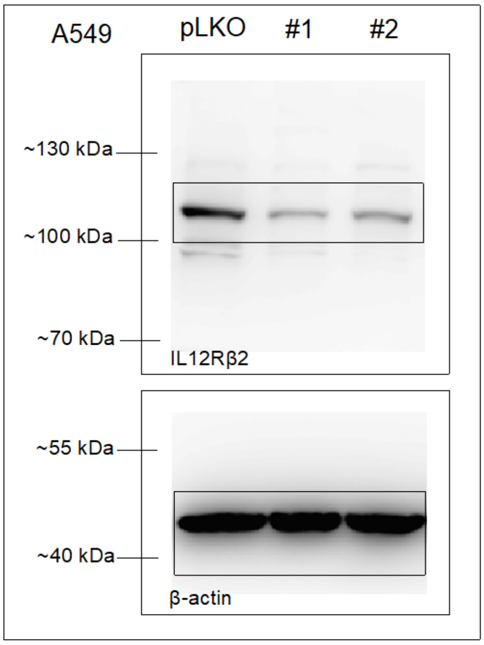

Supplementary Fig. 7e

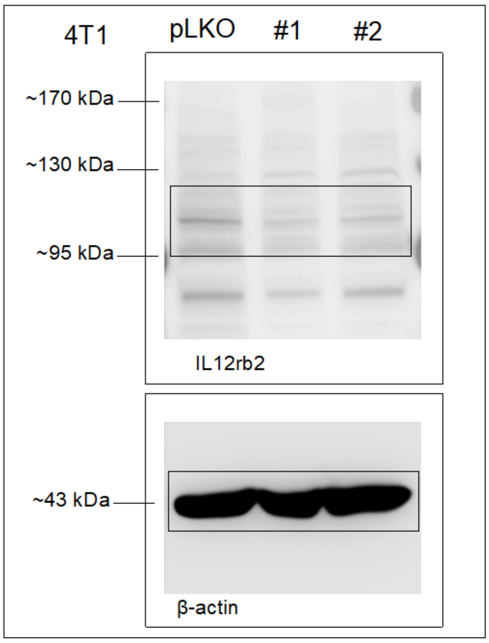

Supplementary Tables

Supplementary Table 1 EMT phenotypes in different cell lines used in this study

	A549	OECM1	SAS	MAD-MB-231	MCF7	4T1	LLC
Epithelial markers	E-cadherin γ-catenin	E-cadherin γ-catenin	E-cadherin γ-catenin	-	E-cadherin γ-catenin	E-cadherin	-
Mesenchymal markers	N-cadherin Vimentin	Vimentin	N-cadherin Vimentin	Vimentin	-	Vimentin	N-cadherin
EMT TFs	Zeb1	Zeb1 Twist1 Snail	Zeb1 Twist1 Snail	Zeb1 Slug	-	Twist1 Snail	Twist1 Slug
EMT phenotype	Hybrid E/M	Hybrid E/M	Hybrid E/M	M	E	Hybrid E/M	M

E, epithelial;
M, mesenchymal.

Supplementary Table 2 Characteristics of 91 head and neck cancer patients for immunohistochemical analysis of IL12Rβ2

Variables	Case No.
Age	
<50	16
≧50	75
Gender	
Male	84
Female	7
T stage	
1~2	43
3~4	48
N stage	
0	57
1~3	34
Site	
Hypopharynx	26
Larynx	8
Oropharynx	2
Oral cavity	55

Supplementary Table 3 A cross-table to show the correlation of IL12Rβ2 expression and the development of subsequent metastasis in patients

	Metastasis			
IL12Rβ2	No	Yes	Total	p value
Low	29	17	46	0.016
High	17	28	45	
Total	46	45	91	
Low H score, 0 ~ 127; high H score, 128 ~ 300.				

Supplementary Table 4 The working concentration for inhibitor used in this study

Inhibitor	Concentration
TGF-beta receptor inhibitor LY 364947	5 mM
Wnt signal inhibitor XAV939	10 mM
Noggin	50 ng per ml
Wnt signal inhibitor IWP2	10 mM
EGFR inhibitor JNJ 28871063	0.2 mM
Notch signal inhibitor DAPT	10 mM
Sonic hedgehog signal inhibitor SANT1	10 mM
MET Inhibitor PHA 665752	0.2 mM
JAK inhibitor I	1 mM
NF-kB inhibitor parnetholide	10 mM
Ruxolitinib	10 mM
