# Supplementary material for: Macrophage-secreted interleukin-35 regulates cancer cell plasticity to facilitate metastatic colonization
Source: Nat Commun. 2018 Sep 14;9:3763. doi: 10.1038/s41467-018-06268-0 (PMC6138674; doi:10.1038/s41467-018-06268-0)
Supplement: Supplementary file 2 — Description of additional supplementary files [file 41467_2018_6268_MOESM2_ESM.docx]

**Description of Additional Supplementary Files**

**File Name: Supplementary Data 1**

**Description: microarray data.** Upregulated and downregulated genes in cDNA microarray analysis of M2-treated A549 vs. M1-treated A549

**File Name: Supplementary Data 2**

**Description: microarray data.** Upregulated and downregulated genes in cDNA microarray analysis of M1-treated A549 vs. M0-treated A549

**File Name: Supplementary Data 3**

**Description: microarray data.** Upregulated and downregulated genes in cDNA microarray analysis M2-treated A549 vs. M0-treated A549

**File Name: Supplementary Data 4**

**Description: microarray data.** Upregulated genes in cDNA microarray analysis of mTAMs vs. pTAMs from 4T1-BALB/c syngeneic orthotopic model

**File Name: Supplementary Data 5**

**Description: primer sequence.** Information of RT-qPCR and qChIP primers used in this study

**File Name: Supplementary Data 6**

**Description: antibody information.** Information of antibodies used in this study
